# Food hazards on the European Union market: The data analysis of the Rapid Alert System for Food and Feed

**DOI:** 10.1002/fsn3.1448

**Published:** 2020-02-11

**Authors:** Marcin Pigłowski

**Affiliations:** ^1^ Department of Commodity and Quality Management Faculty of Entrepreneurship and Quality Science Gdynia Maritime University Gdynia Poland

**Keywords:** cluster analysis, hazards, notifications, pivot tables, Rapid Alert System for Food and Feed

## Abstract

The aim of the study was to examine similarities in notifications on main hazards within food reported in the Rapid Alert System for Food and Feed (RASFF) in 1979–2017. The main problems were mycotoxins in nuts, pathogenic microorganisms in poultry meat and fish, pesticide residues in fruits and vegetables, and heavy metals in fish. The increase in the number of notifications has been observed since 2002/2003. Products were notified mainly by Italy, Germany, and United Kingdom and originated from Asian and European Union countries. The notification basis was border control and official control, and the notification type was border rejections, information, and alerts. Notified products were not distributed and not placed on the market, distribution status could be also not specified, or distribution was possible, also to other countries. The risk decision on hazard was usually not made. Products were redispatched, withdrawn from the market, and destroyed, or import was not authorized. Remarks, which can be used to improve the RASFF database, were also presented. It was further pointed out that European law should significantly reduce the use of pesticides, drugs, and food additives, and European agriculture should be reoriented from an intensive farming to a more sustainable and ecological one.

## Graphical abstract

In my article, I have presented notifications regarding the eight most frequently reported hazards in the Rapid Alert System for Food and Feed, using cluster analysis. Analysis of notifications reported in the RASFF may be helpful in the activities of European and national government bodies and contribute to increasing food safety to the European consumer.

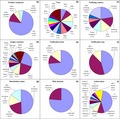

## 1 INTRODUCTION

Among various warning systems for food, the best known are the International Food Safety Authorities Network (INFOSAN) managed by the World Health Organization (WHO; Cheftel, 2011), the Reportable Food Registry (RFR) in the United States (Zach, Doyle, Bier, & Czuprynski, 2012), the Global Public Health Intelligence Network (GPHIN) in Canada (Van Asselt, Meuwissen, Van Asseldonk, Teeuw, & Van Der Fels‐Klerx, 2010; Van Der Spiegel, Van Der Fels‐Klerx, & Marvin, 2012), and the Rapid Alert System for Food and Feed (RASFF) in the European Union (EU; Cheftel, 2011; Van Asselt et al., 2010; Van Der Spiegel et al., 2012; Zach et al., 2012). The RASFF has been in operation since 1979, but its current legal basis was set out in the Regulation (EC) n. 178/2002 (general principles and requirements of food law; European Parliament & Council, 2002) and in the Regulation (EU) n. 16/2011 (laying down implementing measures for the RASFF; Commission, 2011). This system enables exchange of information on food safety risks between contact points of its members, that is, European Union countries national food safety authorities; European Commission; the European Food Safety Authority (EFTA); the European Free Trade Association Surveillance Authority (ESA); and Norway, Iceland, Liechtenstein, and Switzerland. All EU countries and also Norway, Iceland, and Liechtenstein form a common market with the free movement of people, capital, goods, and services (the European Economic Area, i.e., the EEA), inhabited by over half a billion people. Switzerland is not an EEA country, but is a member of the RASFF (European Commission, 2018a).

According to FTSE Russell (a company belonging to the London Stock Exchange Group and providing global benchmark indices), 15 out of 28 EU countries have developed markets (there are only 26 countries in the world in this group; FTSE Russell, 2018). To ensure food safety on such a large and important market, trust and close cooperation between EU institutions and authorities of individual member country are necessary. The RASFF is a good example of this collaboration, enabling the exchange of information on dangerous food products that come from EU producers as well as imported products, which may consequently contribute to the improvement of food quality.

There are four types of RASFF notifications: alerts, information (including information for attention and information for follow‐up), border rejections, and news. Alert notifications are reported when food or feed presenting a serious health risk is on the market and rapid action is required (e.g., product withdrawal). The purpose of the alert notification is to provide information to other RASFF members about risks that have occurred in the common market so that they can also take appropriate measures. Information notifications are sent when risk has been identified for food or feed placed on the market, but other RASFF members do not need to take rapid action. This is the case if the product has not yet reached their markets or because the nature of the risk rapid action is not needed. Border rejections relate to food or feed that has been examined and rejected at the EU's external border (in a broader sense also the EEA) if a health risk is found in the product. Notifications of this type are forwarded to all EEA border posts to prevent attempts to reenter this product through another border post. Any other information concerning the safety of food or feed that has not been classified as alert notification or information notification, but which can be interesting for the control authorities, is defined as news (European Commission, 2018b). The vast majority among 51,155 notifications in the RASFF to the end of 2017 concerned food (45,761 notifications, 89.5%), then feed (3,187, 6.2%), and food contact material (2,207, 4.3%; European Commission, 2018c).

Bánáti (2011) notes that the RASFF proved to be a useful tool, but it cannot prevent contaminated food entering the food chain on the market. However, Hargin and Shears (2013) believe that the RASFF can help identify potential problems before they become widespread. Similarly, Marvin, Kleter, Frewer, et al. (2009) claim that the RASFF is an early warning, protection system against certain hazards that may be disseminated from one member state. Trevisani and Rosmini (2008) believe that the RASFF alerts are useful for recalling foods or for rating risks of imported foods and products before they are placed on the market. Hirschauer and Zwoll (2008) notice that the RASFF aims at effective communication and after‐the‐fact responsiveness. Similarly, Marvin, Kleter, Prandini, Dekkers, and Bolton (2009) note that within the RASFF, hazards are detected only after they occurred; therefore, any intervention will be reactive. However, they add that trends in the RASFF data can provide a useful basis to identify hazards that are likely to increase in future. Banach Stratakou Van Der Fels‐Klerx Den Besten and Zwietering (2016) claim that the RASFF can be used in risk assessment. Hargin and Shears (2013) believe that the RASFF effectiveness relies on its simplicity and legal obligation of its members to notify serious health risk. Spichtinger and Astley (2009) note that the RASFF plays a vital role in ensuring food safety in Europe and demonstrates a proactive approach for consumers.

The main problems resulting from the RASFF notifications are signaled in annual reports. In the report for 2017 pathogenic microorganisms (including food poisoning), mycotoxins and heavy metals were indicated as the most frequently notified hazards (European Union, 2018). Although RASFF annual reports draw attention to the most important, current problems in notifications (and wider food safety), the data are often presented in a simplified and general way and related to one year or only several last years. Therefore, the aim of the study was to examine similarities in the RASFF notifications in 1979–2017 on main hazard categories within food taking into account: product category, year, notifying country, origin country, notification basis, notification type, distribution status, risk decision, and action taken, based on the data from the RASFF database.

## 2 MATERIAL AND METHODS

### 2.1 Data

The data were extracted from the RASFF database in the form of notification lists in Excel and covered notifications on food in the period from 1 January 1979 to 31 December 2017. The single notification list contained data in the following columns: product category, date, reference, product type, notification type, notification basis, notified by, countries concerned, subject, action taken, distribution status, and risk decision. Such a notification list did not contain data on hazard category, because one notification could relate to more than one hazard (however, this only applies to approx. 2% of all notifications; European Commission, 2018c). Therefore, notification list was obtained separately for each hazard category. In fact, the number of notifications in this period was smaller. All notification lists were next combined in Excel into one table of 45,761 notifications. The data on notifications were then ordered and finally the table with notifications contained following columns: hazard category (this column was added), product category, year (these data were obtained from column “reference”), notifying country (the name of column “notified by” was changed), origin country (these data were obtained from column “countries concerned”), notification basis, notification type, distribution status, risk decision, and action taken. When more than one origin country was indicated, it was necessary to adopt the first country among the mentioned. However, in the case of missing data on origin country, and also on notification basis, distribution status, and action taken, the phrase “not specified” was entered. All these actions were necessary for statistical calculations. Data on date and subject were omitted because they were too varied. Among the data in the column “product type,” only “food” was selected, as already mentioned before.

### 2.2 Methods

Similarities in notifications within all hazard categories were initially examined using a joining cluster analysis in Statistica 12 (Figures S1–S9). The nine pivot tables for this analysis were first prepared in Excel and then transferred to Statistica 12. They contained names of hazard categories in columns (in each of the tables) and, in the following tables, names of product category, year, notifying country, origin country, notification basis, notification type, distribution status, risk decision, and action taken in rows. In each table (except the table for years), data on the number of notifications have been ordered in descending order. In the joining cluster analysis, the Ward's method as a linkage rule was used. In this method, an analysis of variance to evaluate distances between clusters is applied, which aims at minimizing the sum of squares of any two (hypothetical) clusters that can be created. Ward's method is considered very efficient (the division into clusters is the most visible); however, it tends to form clusters of small size.

Hazard categories for which the number of notifications exceeded the arithmetic mean value (in relation to all notifications) were taken for further statistical research. This mean was computed in Excel and was exceeded in the case of eight hazard categories. The arithmetic value of numbers (scores) in the distribution is the sum of numbers (scores) divided by the number of these numbers, and it is the most often used measure of central tendency. Then, the standardized *z*‐score *Z* was also computed, where *S* was standard deviation. A standardized z‐score *Z* represented both the relative position of an individual score in a distribution as compared to the mean and variation of score in the distribution (Crewson, 2008). In the case of all hazard categories, in which the number of notifications exceeded the mean value, standardized z‐score *Z* was positive, what meant that the score was above the distribution mean. For all other 19 hazard categories, this score was negative.

The percentage shares of values within particular variables (for each examined hazard category) were presented in pie charts (Figures 1, 2, 3, 4, 5, 6, 7, 8) using abbreviations for.

- product categories: bivalve mollusks—bivalve mollusks and products thereof; cephalopods—cephalopods and products thereof; cereals—cereals and bakery products; cocoa, coffee, tea—cocoa and cocoa preparations, coffee, and tea; crustaceans—crustaceans and products thereof; dietetic foods—dietetic foods, food supplements, fortified foods; fats, oils—fats and oils; fish—fish and fish products; fruits, vegetables—fruits and vegetables; herbs, spices—herbs and spices; honey—honey and royal jelly; meat—meat and meat products (other than poultry); mollusks—mollusks and products thereof (obsolete); nuts, seeds—nuts, nut products, and seeds; poultry meat—poultry meat and poultry meat products; prepared dishes—prepared dishes and snacks; soups, broths—soups, broths, sauces, and condiments,
- notification basis: border control‐detained—border control‐consignment detained; border control‐released—border control‐consignment released; official control—official control on the market,
- distribution status: distr. to m. countries—distribution to other member countries; distribution (possible)—distribution on the market (possible); distribution restricted—distribution restricted to notifying country; information not available—information on distribution not (yet) available; no distr. from n. country—no distribution from notifying country; no longer on market—product (presumably) no longer on the market; not placed on market—product not (yet) placed on the market; product consumed—product already consumed,
- action taken: customs seals—placed under customs seals; recall—recall from consumers; recall/withdrawal—product recall or withdrawal; redispatch/destruction—redispatch or destruction; withdrawal—withdrawal from the market.

**Figure 1:**
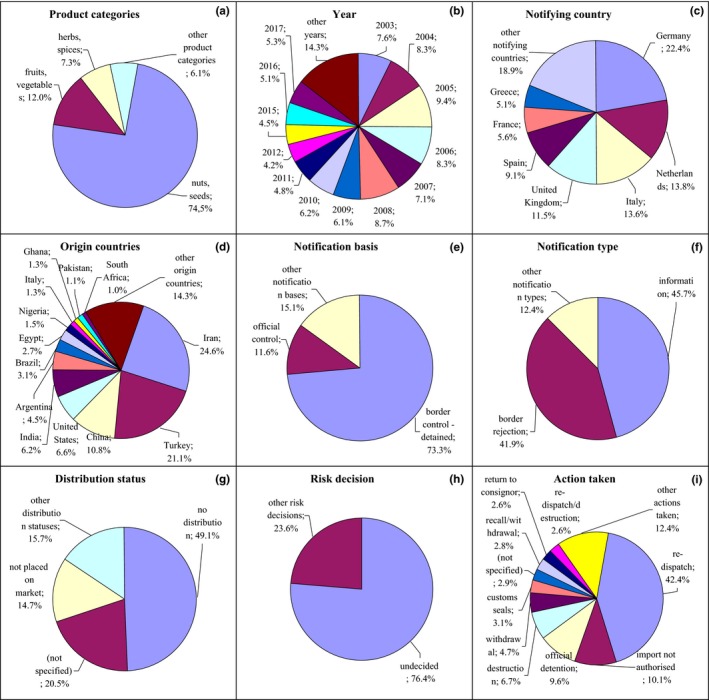
Notifications on mycotoxins in the RASFF. (a) Product category: nuts, seeds—nuts, nut products, and seeds; fruits, vegetables—fruits and vegetables; herbs, spices—herbs and spices; (e) notification basis: border control‐detained—border control‐consignment detained; official control—official control on the market; (g) distribution status: not placed on market—product not (yet) placed on the market; (i) action taken: withdrawal—withdrawal from the market; customs seals—placed under customs seals; recall/withdrawal—product recall or withdrawal; redispatch/destruction—redispatch or destruction

**Figure 2:**
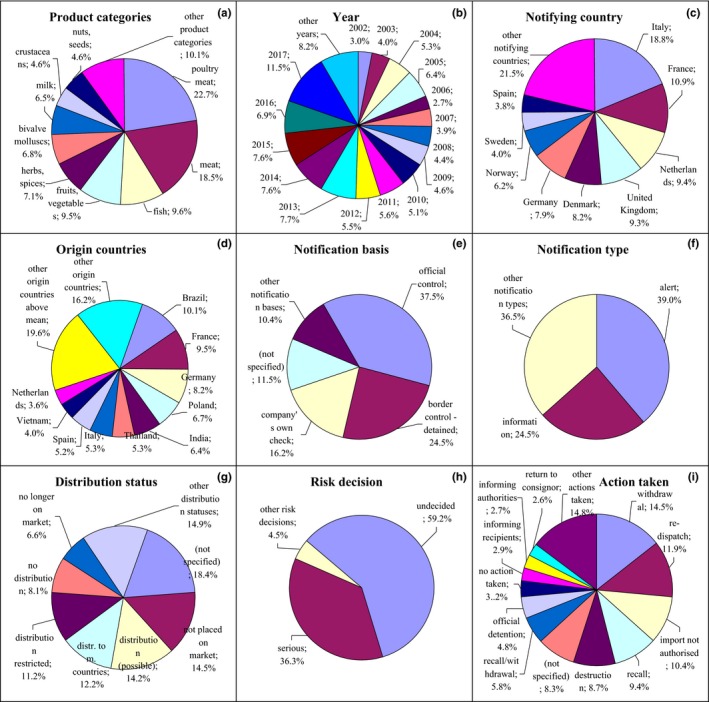
Notifications on pathogenic microorganisms in the RASFF. (a) product category: poultry meat—poultry meat and poultry meat products; meat—meat and meat products (other than poultry); fish—fish and fish products; fruits, vegetables—fruits and vegetables; herbs, spices—herbs and spices; bivalve mollusks—bivalve mollusks and products thereof; milk—milk and milk products; crustaceans—crustaceans and products thereof; nuts, seeds—nuts, nut products, and seeds; (e) notification basis: official control—official control on the market; border control‐detained—border control‐consignment detained; (g) distribution status: not placed on market—product not (yet) placed on the market; distribution (possible)—distribution on the market (possible); distr. to m. countries—distribution to other member countries; distribution restricted—distribution restricted to notifying country; no longer on market—product (presumably) no longer on the market; (i) action taken: recall—recall from consumers; recall/withdrawal—product recall or withdrawal

**Figure 3:**
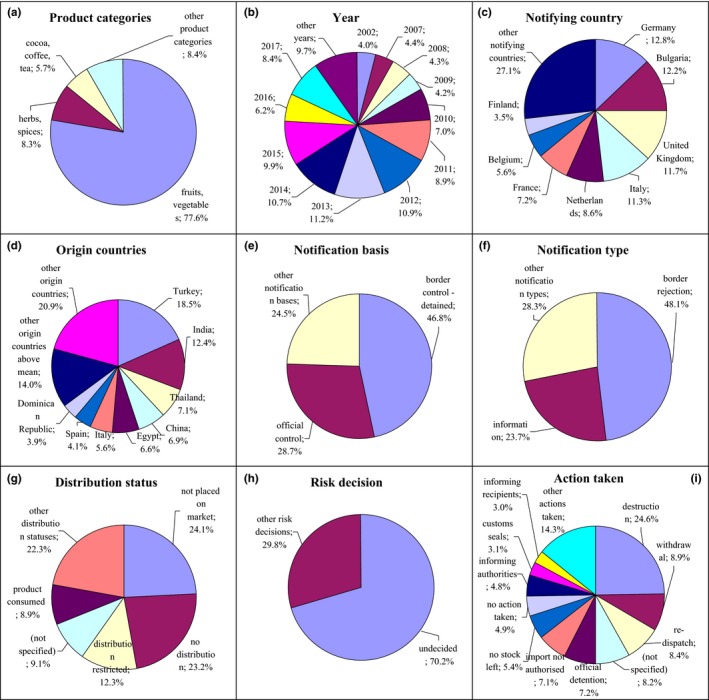
Notifications on pesticide residues in the RASFF. (a) product category: fruits, vegetables—fruits and vegetables; herbs, spices—herbs and spices; cocoa, coffee, tea—cocoa and cocoa preparations, coffee, and tea; (e) notification basis: border control‐detained—border control‐consignment detained; official control—official control on the market; (g) distribution status: not placed on market—product not (yet) placed on the market; distribution restricted—distribution restricted to notifying country; product consumed—product already consumed; (i) action taken: withdrawal—withdrawal from the market; customs seals—placed under customs seals

**Figure 4:**
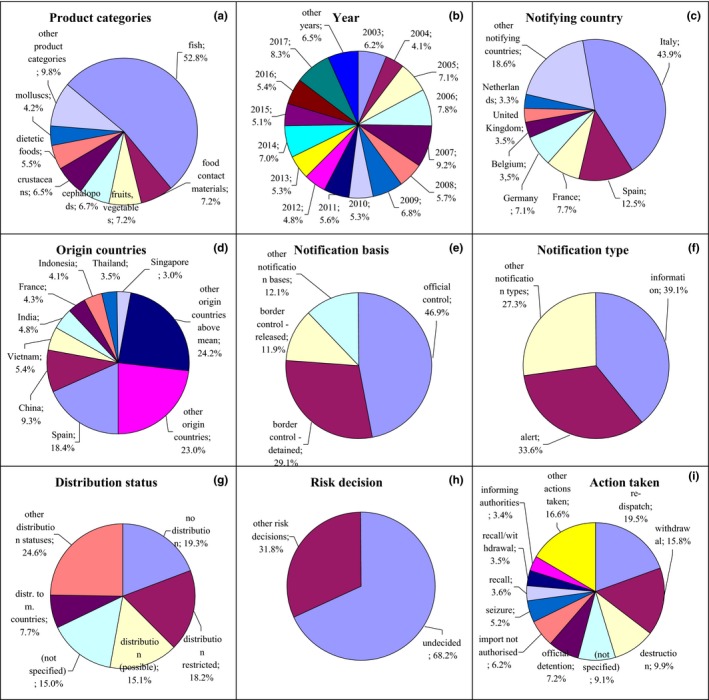
Notifications on heavy metals in the RASFF. (a) product category: fish—fish and fish products; fruits, vegetables—fruits and vegetables; cephalopods—cephalopods and products thereof; crustaceans—crustaceans and products thereof; dietetic foods—dietetic foods, food supplements, fortified foods; mollusks—mollusks and products thereof (obsolete); (e) notification basis: official control—official control on the market; border control‐detained—border control‐consignment detained; border control‐released—border control‐consignment released; (g) distribution status: distribution restricted—distribution restricted to notifying country; distribution (possible)—distribution on the market (possible); distr. to m. countries—distribution to other member countries; (i) action taken: withdrawal—withdrawal from the market; recall—recall from consumers; recall/withdrawal—product recall or withdrawal

**Figure 5:**
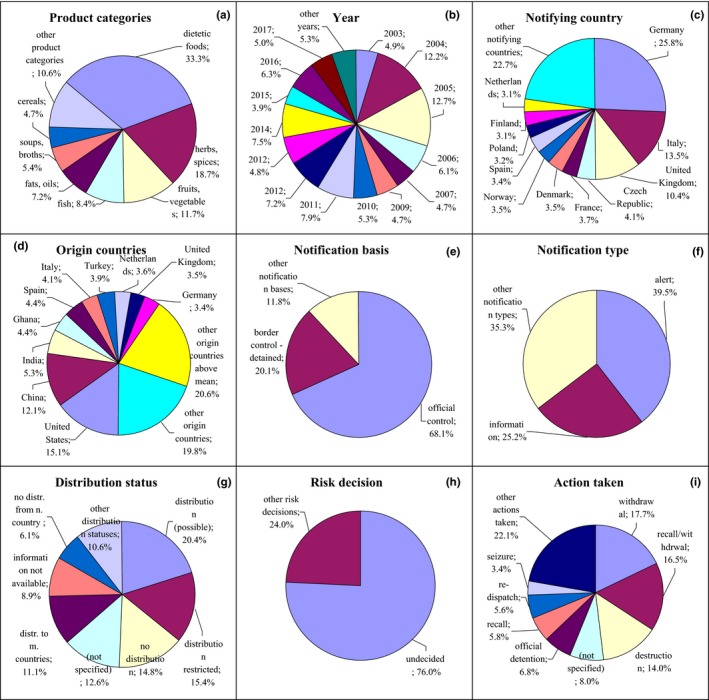
Notifications on composition in the RASFF. (a) product category: dietetic foods—dietetic foods, food supplements, fortified foods; herbs, spices—herbs and spices; fruits, vegetables—fruits and vegetables; fish—fish and fish products; fats, oils—fats and oils; soups, broths—soups, broths, sauces, and condiments; cereals—cereals and bakery products; (e) notification basis: official control—official control on the market; border control‐detained—border control‐consignment detained; (g) distribution status: distribution (possible)—distribution on the market (possible); distribution restricted—distribution restricted to notifying country; distr. to m. countries—distribution to other member countries; information not available—information on distribution not (yet) available; no distr. from n. country—no distribution from notifying country; (i) action taken: withdrawal—withdrawal from the market; recall/withdrawal—product recall or withdrawal; recall—recall from consumers

**Figure 6:**
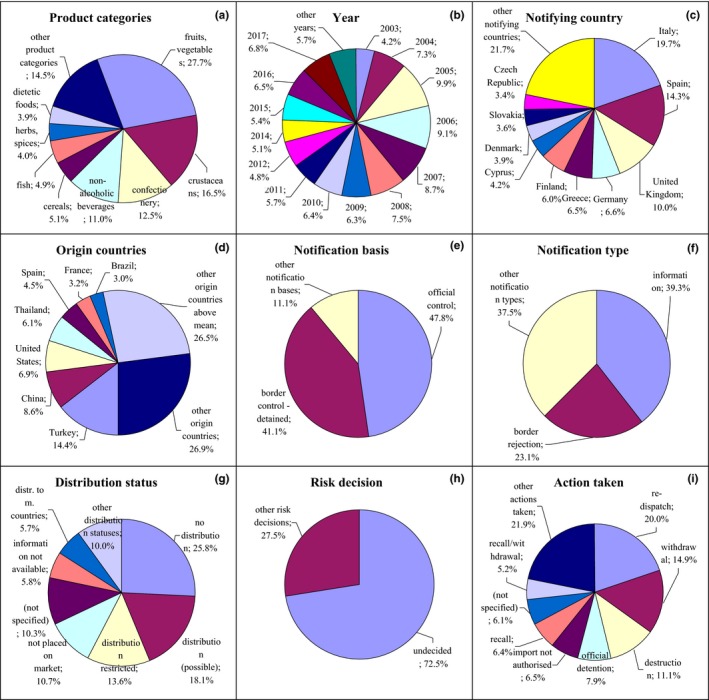
Notifications on food additives and flavorings in the RASFF. (a) product category: fruits, vegetables—fruits and vegetables; crustaceans—crustaceans and products thereof; cereals—cereals and bakery products; fish—fish and fish products; herbs, spices—herbs and spices; dietetic foods—dietetic foods, food supplements, fortified foods; (e) notification basis: official control—official control on the market; border control‐detained—border control‐consignment detained; (g) distribution status: distribution (possible)—distribution on the market (possible); distribution restricted—distribution restricted to notifying country; not placed on market—product not (yet) placed on the market; information not available—information on distribution not (yet) available; distr. to m. countries—distribution to other member countries; (i) action taken: withdrawal—withdrawal from the market; recall—recall from consumers; recall/withdrawal—product recall or withdrawal

**Figure 7:**
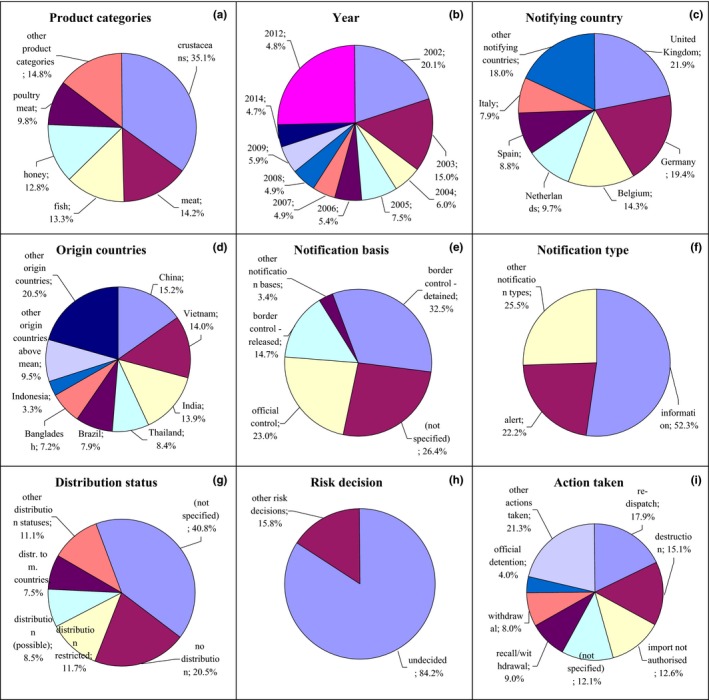
Notifications on residues of veterinary medicinal products in the RASFF. (a) product category: crustaceans—crustaceans and products thereof; meat—meat and meat products (other than poultry); fish—fish and fish products; honey—honey and royal jelly; poultry meat—poultry meat and poultry meat products; (e) notification basis: border control‐detained—border control‐consignment detained; official control—official control on the market; border control‐released—border control‐consignment released; (g) distribution status: distribution restricted—distribution restricted to notifying country; distribution (possible)—distribution on the market (possible); distr. to m. countries—distribution to other member countries; (i) action taken: recall/withdrawal—product recall or withdrawal; withdrawal—withdrawal from the market

**Figure 8:**
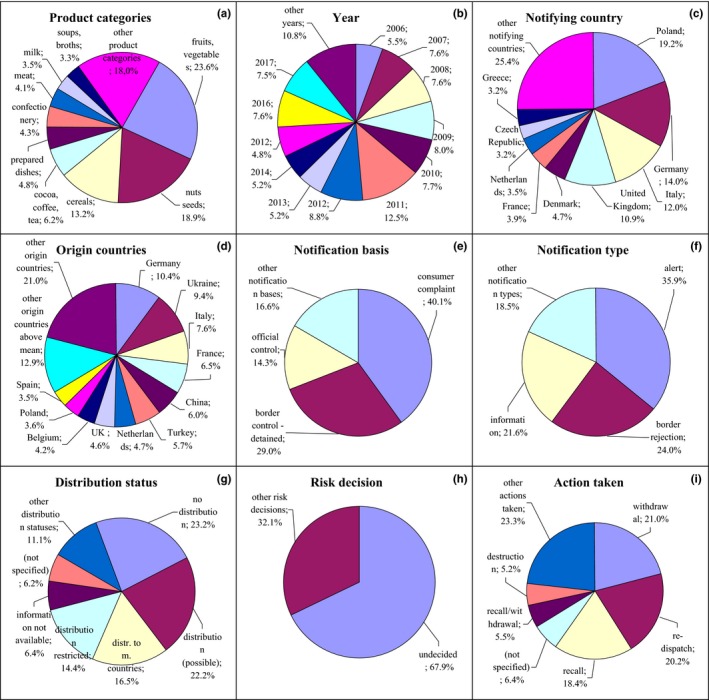
Notifications on foreign bodies in the RASFF. (a) product category: fruits, vegetables—fruits and vegetables; nuts, seeds—nuts, nut products and seeds; cereals—cereals and bakery products; cocoa, coffee, tea—cocoa and cocoa preparations, coffee, and tea; prepared dishes—prepared dishes and snacks; meat—meat and meat products (other than poultry); milk—milk and milk products; soups, broths—soups, broths, sauces, and condiments; (e) notification basis: border control‐detained—border control‐consignment detained; official control—official control on the market; (g) distribution status: distribution (possible)—distribution on the market (possible); distr. to m. countries—distribution to other member countries; distribution restricted—distribution restricted to notifying country; information not available—information on distribution not (yet) available; (i) action taken: withdrawal—withdrawal from the market; recall—recall from consumers; recall/withdrawal—product recall or withdrawal

These results were also supported by charts (Figures S10–S73) created with the use of a two‐way joining cluster analysis method. The sixty‐four pivot tables (eight tables for each of the hazard category studied) for this analysis were prepared in Excel and then transferred to Statistica 12. They contained names of product categories in columns (in each of the tables) and, in the following tables, names of year, notifying country, origin country, notification basis, notification type, distribution status, risk decision, and action taken in rows. In each table (except the table for years), data on the number of notifications have been ordered in descending order. The charts presented similarities between product categories and other variables within particular hazard categories using contours, which thicken to the center of cluster changing colors (from green, through yellow, orange, red to brown). Interpretation of charts in some rare cases was impeded—if the number of notifications was large, but scattered, clusters could be only green or they were not formed. To improve the readability of charts, the number of variable values was limited to twenty. Long names were abbreviated, and full names were given below the charts.

## 3 RESULTS

The number and percentage shares of the RASFF notifications within particular hazard categories in 1979–2017 were presented in Table 1.

**Table 1:** Number and percentage shares of the RASFF notifications within hazard categories in 1979–2017

Hazard category	Number	Percentage	Hazard category	Number	Percentage
Mycotoxins	10,507	23.0	Biocontaminants	678	1.5
Pathogenic microorganisms	8,322	18.2	Parasitic infestation	650	1.4
Pesticide residues	4,002	8.7	Genetically modified food or feed	573	1.3
Heavy metals	2,735	6.0	Novel food	541	1.2
Composition	2,637	5.8	Biotoxins (other)	454	1.0
Food additives and flavorings	2,560	5.6	Radiation	437	1.0
Residues of veterinary medicinal products	2,007	4.4	Migration	385	0.8
Foreign bodies	1,706	3.7	Labeling absent/incomplete/incorrect	365	0.8
Poor or insufficient controls	1,329	2.9	Packaging defective/incorrect	360	0.8
Adulteration/fraud	1,212	2.6	Not determined/other	194	0.4
Non‐pathogenic microorganisms	1,077	2.4	Feed additives	84	0.2
Allergens	1,055	2.3	Chemical contamination (other)	75	0.2
Industrial contaminants	915	2.0	TSEs (transmissible spongiform encephalopathies)	75	0.2
Organoleptic aspects	826	1.8	Total	45,761	100

In the case of eight hazard categories, number of notifications exceeded the mean value and they were as follows: mycotoxins, pathogenic microorganisms, pesticide residues, heavy metals, composition, food additives and flavorings, residues of veterinary medicinal products, and foreign bodies (they accounted for over 75% of all notifications).

### 3.1 Hazard categories most often notified

The results of a joining cluster analysis for hazard categories taking into account different variables were presented in Figures S1–S9. In this analysis, one‐ or two‐element clusters (mycotoxins or mycotoxins and pathogenic microorganisms) were distinctly created. It confirmed that these hazards dominated in the number of notifications. One‐element clusters concerned mycotoxins created in the case of variable product category (Figure S1), origin country (Figure S4), notification basis (Figure S5), notification type (Figure S6), distribution status (Figure S7), and action taken (Figure S9). In turn, two‐element clusters consisting of mycotoxins and pathogenic microorganisms were formed in the case of years (Figure S2), notifying countries (Figure S3), and risk decision (Figure S8), which indicated similar character of notifications within these two hazard categories and particular variables mentioned. However, similar character had also notifications on food additives and flavorings and heavy metals in the case of years, notification basis and action taken (Figures S2, S5, and S9, respectively), residues of veterinary medicinal products and composition in the case of notifying country (Figure S3), residues of veterinary medicinal products and heavy metals in the case of origin country (Figure S4), residues of veterinary medicinal products and food additives and flavorings in the case of notification type (Figure S6), and composition and heavy metals in the case of notification type and distribution status (Figures S6 and S7, respectively). The nature of these similarities can be more clearly explained while presenting the results of a two‐way joining cluster analysis.

What is characteristic, however, almost all of the eight hazard categories examined (regardless of the variable) are located close to each other (on the right side of Figures S1–S9), forming clusters or subclusters, clearly distinguishing themselves from the other hazard categories. This is also confirmed by the high values of linkage distance between them and other hazard categories.

### 3.2 Detailed analysis in hazard categories

The percentage share of notifications on the examined hazard categories was presented in panels in Figures 1, 2, 3, 4, 5, 6, 7, 8 and the related to them results of a two‐way joining cluster analysis were presented in Figures S10–S73.

#### 3.2.1 Mycotoxins

In the case of mycotoxins, the most frequently notified were aflatoxins, ochratoxin A, fumonisins, and deoxynivalenol. Notifications related first at all to nuts and seeds (74.5%) after 2003 with the higher number in 2005, also fruits and vegetables (12.0%) and herbs and spices (7.3%; Figures 1a,b, Figure S10). These products were notified mainly by Germany (22.4%), the Netherlands (13.8%), and Italy (13.6%; Figure 1c, Figure S11) and originated from Iran (24.8%), Turkey (21.1%), and China (10.8%; Figure 1d, Figure S12). The notification basis was border control, after which the consignment was detained (73.3%), and also official control on the market (11.6%; Figure 1e, Figure S13) and the notification type were border rejection (41.9%) and information (45.7%; Figure 1f, Figure S14). Products were not distributed (49.1%), or the distribution status was not specified (20.5%; Figure 1g, Figure S15). Risk decision was usually not made (76.4%; Figure 1h, Figure S16), and products were mainly redispatched (42.4%; Figure 1i, Figure S17).

#### 3.2.2 Pathogenic microorganisms

Notifications on pathogenic microorganisms related mainly to *Salmonella* spp., *Listeria monocytogenes*, *Escherichia coli*, *Vibrio* spp., *Bacillus cereus*, *Campylobacter* spp., and noroviruses. They were the most varied notifications among the examined hazard categories; however, they related mainly to poultry meat (22.7%) after 2002 with the highest number in 2017 (Figure 2a,b, Figure S18). They were reported mainly by Italy, France, the Netherlands, United Kingdom, Denmark, and Germany and originated from Brazil, France, Germany, Poland, and the Netherlands. However, the other product categories were also meat (18.5%) notified by Italy and originated from Spain, France, Germany, Poland, and the Netherlands; fish (9.6%) notified by Italy and originated from Denmark and Poland; bivalve mollusks (6.8%) notified by Italy and originated from this country and crustaceans (4.6%) and mollusks also notified by Italy and originated from Thailand and India; fruits and vegetables (9.5%) notified by United Kingdom and originated from Thailand, India, and Bangladesh; herbs and spices (7.1%) notified by the Netherlands and originated from Thailand; nuts and seeds (4.6%) notified by Italy and Greece and originated from Brazil and India; and milk (6.5%) notified by and originated from France (Figure 2c and Figure S19, Figure 2d and Figure S20). The notification basis for these products was official control on the market (37.5%) and border control, after which the consignment was detained (24.5%; Figure 2e, Figure S21), and also company's own check (16.2%). The notification type was mainly alert (39.0%; Figure 2f, Figure S22) and information (24.5%). Distribution status of these products was usually not specified (18.4%), they were also not (yet) placed on the market (14.5%), distribution on the market was possible (14.2%), there was also distribution to other member countries (12.2%), or distribution was restricted to the notified country (11.2%; Figure 2g, Figure S23). Risk decision was usually not made (59.2%); however, risk could be also specified as serious (36.3%; Figure 2h, Figure S24). These products were mainly withdrawn from the market (14.5%) and redispatched (11.9%), or import was not authorized (10.4%; Figure 2i, Figure S25).

#### 3.2.3 Pesticide residues

Notifications on pesticide residues referred to the presence of, for example, chlorpyrifos, methomyl, dimethoate, carbendazim, omethoate, methamidophos, or oxamyl. Pesticides were notified mainly after 2007 in fruits and vegetables (77.6%), also in herbs and spices (8.3%) as well as cocoa, coffee, and tea (5.7%; Figure 3a,b, Figure S26). These products were notified by Germany (12.8%), Bulgaria (12.2%), United Kingdom (11.7%), Italy (11.3%), the Netherlands (8.6%), and France (7.2%; Figure 3c, Figure S27) and originated from Turkey (18.5%) and India (12.4%; Figure 3d, Figure S28). The notification basis was border control, after which the consignment was detained (46.8%) and official control on the market (28.7%; Figure 3e, Figure S29). The notification type was border rejection (48.1%) and information (23.7%; Figure 3f, Figure S30). Products were not (yet) placed on the market (24.1%) and not distributed (23.2%), distribution was restricted to notifying country (12.3%), or products were already consumed (8.9%; Figure 3g, Figure S31). Risk decision was usually not made (70.2%; Figure 3h, Figure S32), and products were mainly destroyed (24.6%; Figure S33); however, they could also be withdrawn from the market (8.9%) and redispatched (8.4%) or the action was not specified (8.2%; Figure 3i, Figure S33).

#### 3.2.4 Heavy metals

The most frequently reported heavy metals were mercury, cadmium, lead, chromium, arsenic, tin, and nickel. The highest number of notifications on heavy metals related to fish (52.8%) and food contact materials (7.2%) after 2003 (Figure 4a,b, Figure S34). They were notified by Italy (43.9%) and Spain (12.5%; Figure 4c, Figure S35) and originated from Spain (18.4%) and Vietnam (5.4%; Figure 4d, Figure S36), and also from other Asian countries, for example, China, India, Indonesia, and Thailand as well as from France. The notification basis for these products was official control on the market (46.9%; Figure 4e, Figure S37) and border control, after which the consignment was detained (29.1%) or released (11.9%). The notification type was information (39.1%) and alert (33.6%; Figure 4f, Figure S38). Products were not distributed (19.3%), distribution was restricted to the notified country (18.2%), or distribution on the market was possible (15.1%; Figure 4g, Figure S39). Risk decision was usually not made (68.2%; Figure 4h, Figure S40), and products were redispatched (19.5%) and withdrawn from the market (15.8%); some products were also destroyed (9.9%), action was not specified (9.1%), or they were officially detained (7.2%; Figure 4i, Figure S41).

#### 3.2.5 Composition

Notifications on composition concerned mainly: unauthorized colors: Sudan 1, Sudan 4, Rhodamine B and Para Red; unauthorized substances: 1,3‐dimethylamylamine, sibutramine, sildenafil, and yohimbine; high content of aluminum, cyanide, iodine, and morphine; and too high content of erucic acid, nitrate, vitamins, and carbon monoxide treatment. The highest number of notifications on composition related to dietetic foods (33.3%) after 2012, also herbs and spices (18.7%) in 2004 and 2005, fruits and vegetables (11.7%) and fish (8.4%; Figure 5a,b, Figure S42). Dietetic foods, herbs and spices, and fruits and vegetables were notified mainly by Germany and fish by Italy (Figure 5c, Figure S43). Dietetic foods originated usually from the United States, China, and India; herbs and spices from Turkey; and fruits and vegetables from China and fish from Vietnam and Spain (Figure 5d, Figure S44). The notification basis for these products was official control on the market (68.1%) and border control, after which the consignment was detained (20.1%; Figure 5e, Figure S45). The notification type was alert (39.5%) and information (25.2%; Figure 5f, Figure S46). Distribution of notified products was various: it was possible (20.4%) and restricted to the notifying country (15.4%). However, products were also not distributed (14.8%), distribution status was not specified (12.6%), or they could be also distributed to other member countries (11.1%) or information on distribution was not available (8.9%; Figure 5g, Figure S47). Risk decision was usually not made (76.0%; Figure 5h, Figure S48), and products were withdrawn (17.7%) or recalled (16.5%; Figure 5i, Figure S49).

#### 3.2.6 Food additives and flavorings

Notifications on food additives and flavorings referred to too high content of sulfite, sorbic acid (E 200), benzoic acid (E 210), polyphosphates (E 452), and sweeteners, undeclared sulfite; too high content of colors: tartrazine (E 102), Sunset Yellow FCF (E 110) and Ponceau 4R/cochineal red A (E 124); and unauthorized use of colors: Sunset Yellow FC (E 110), tartrazine (E 102), azorubine (E 122), amaranth (E 123), Ponceau 4R/cochineal red A (E 124), erythrosine (E 127), annatto/bixin/norbixin (E 160b), and titanium dioxide (E 171). These notifications related mainly to fruits and vegetables (27.7%) from 2003, but also crustaceans (16.5%; Figure 6a,b, Figure S50). These products were notified mainly by Italy (19.7%) and Spain (14.3%; Figure 6c, Figure S51) and originated from Turkey (14.4%), Vietnam, China, Thailand, and the United States (Figure 6d, Figure S52). The other products notified in this hazard category were confectionary (12.5%) and nonalcoholic beverages (11.0%). Products were notified on the basis of official control on the market (47.8%) or border control, after which the consignment was detained (41.1%; Figure 6e, Figure S53). The notification type was information (39.3%) or border rejection (23.1%; Figure 6f, Figure S54). Products were mainly not distributed (25.8%); however, distribution on the market was also possible (18.1%) or restricted to the notifying country (13.6%), products were not placed on the market (10.7%), or distribution status was not specified (10.3%; Figure 6g, Figure S55). Risk decision on notified products was usually not made (72.5%; Figure 6h, Figure S56). Products were mainly redispatched (20.0%) and withdrawn from the market (14.9%; Figure 6i, Figure S57).

#### 3.2.7 Residues of veterinary medicinal products

Among the residues of veterinary medicinal products, most frequently reported were nitrofuran metabolites, chloramphenicol, malachite green, and leucomalachite green. Notifications related mainly to crustaceans (35.1%) from 2002 (Figure 7a,b, Figure S58). These products were notified by United Kingdom, Germany, Belgium, and the Netherlands (Figure 7c, Figure S59) and originated from China, Vietnam, Bangladesh, Thailand, and India (Figure 7d, Figure S60). In this hazard category, the following were also notified meat (14.2%), fish (13.3%), honey (12.8%), and poultry meat (9.8%). The notification basis for these products was mainly border control, after which the consignment was detained (32.5%) or released (14.7%); however, the basis could be also not specified (26.4%; Figure 7e, Figure S61). The notification type was mainly: information (52.3%; Figure 7f, Figure S62) and alert (22.2%). Distribution status was usually not specified (40.8%), or products were not distributed (20.5%; Figure 7g, Figure S63). Risk decision on notified products was usually not made (84.2%; Figure 7h, Figure S64), and they were redispatched (17.9%) and destroyed (15.1%) or import was not authorized (12.6%; Figure 7i, Figure S65).

#### 3.2.8 Foreign bodies

In the case of foreign bodies, the most frequently reported were glass, plastic, metal, stones, rodent excrements, and dead and living insects and mites. These notifications related mainly to fruits and vegetables (23.6%) and nuts and seeds (18.9%) from 2006; however, for example, cereals (13.2%) and cocoa, coffee, and tea were also notified (6.2%; Figure 8a,b, Figure S66). Fruits and vegetables were notified mainly by Italy and Germany and related to products from Turkey and Tunisia; nuts and seeds were notified by Poland; and concerned products from Ukraine and cereals were notified by Germany and Italy and originated from these countries (Figure 8c and Figure S67, Figure 8d and Figure S68). Products were notified on the basis of consumer complaints (40.1%), border control, after which the consignment was detained (29.0%) and official control on the market (14.3%; Figure 8e, Figure S69). The notification basis was alert (35.9%), border rejection (24.0%), and information (21.6%; Figure 8f, Figure S70). The notified products were usually not distributed (23.2%; Figure 8g, Figure S71); however, distribution could also be possible (22.2%). Risk decision was usually not made (67.9%; Figure 8h, Figure S72), and products were withdrawn from the market (21.0%), redispatched (20.2%), or recalled from consumers (18.4%; Figure 8i, Figure S73).

## 4 DISCUSSION

**Figure 9:**
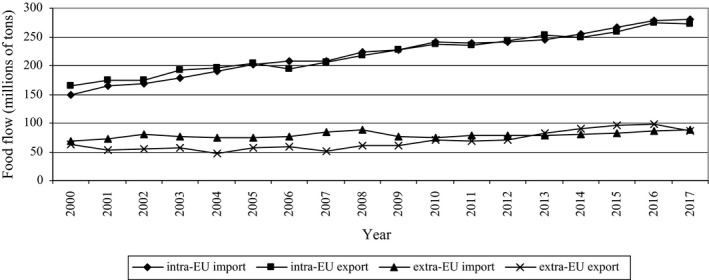
Food flow for EU countries according to Eurostat within the BEC in 2000–2017

According to the data from Eurostat (European Commission, 2018a) within the Broad Economic Categories (BEC), the intra‐EU import and export food flow almost doubled in 2000–2017. In turn, the ratio of extra‐EU import and export changed only slightly. However, the difference between the EU import and export (both intra and extra) significantly increased and in 2017 was three times higher (Figure 9).

**Figure 10:**
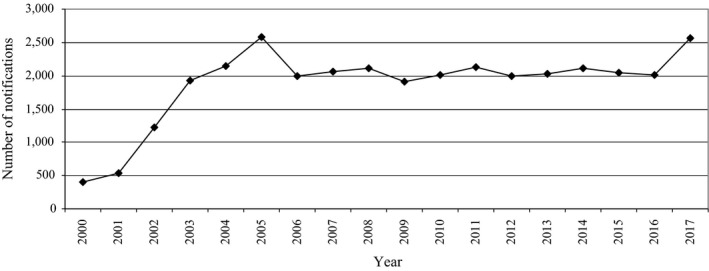
Total number of notifications in the RASFF within the examined hazard categories in 2000–2017

Total number of notifications in the RASFF within the examined hazard categories in this period was presented in Figure 10.

A significant increase in the number of notifications can be observed from 2002/2003 until 2005/2006, and then, it stabilized; however, a significant increase occurred again in 2017. The correlation (Pearson's coefficient *r*) between the food flow and the notifications number in the RASFF in 2000–2017 was moderate (Table 2). What is important is the fact that in the case of intra‐EU import, the correlation was higher than in the case of extra‐EU import, which could indicate that detection of hazards coming from the internal market in relation to the amount of food was higher.

**Table 2:** Correlation between food flow and notifications number in the RASFF in 2000–2017

Food flow	Pearson's coefficient *r*	Statistics *t*
Intra‐EU import	.69	3.77
Extra‐EU import	.53	2.50

Note

Critical statistics in two‐tailed distribution *t*_0.05; 16_ was 2.12.

**Figure 11:**
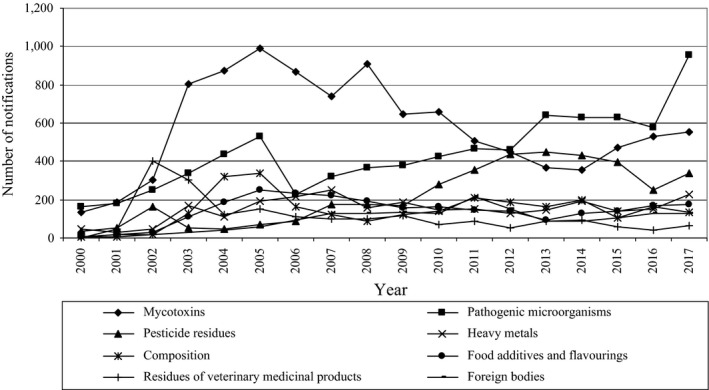
Notifications within particular hazard categories in the RASFF in 2000–2017

However, the correlation based on the food flow did not include food, which was only produced in a given EU country and was not exported to another country. Additionally, the changes in number of notifications in the RASFF differed in particular hazard categories and to a greatest extent, concerned hazards most frequently notified, that is, mycotoxins and pathogenic microorganisms (Figure 11). Particularly disturbing is the very marked increase in the number of notifications regarding pathogenic microorganisms. Lammerding (2013) noted that pathogenic microorganisms can adapt to new niches, new transmission vehicles, new hosts, acquiring new resistance, and virulence mechanisms. She also added that in this context, attention should also be paid to new technologies, consumer preferences, global food trade, and shifts in population demographics. In turn, Shaw and Osborne (2011) indicated that the geographical distribution of plant pathogens may depend on host availability, susceptibility and abundance, suitability of climate conditions, and historical contingency including evolutionary change and also climate change. However, Velásquez, Castroverde, and He (2018) noted that a susceptible plant host would not be infected by a virulent pathogen if environmental conditions were not favorable for disease. Moreover, they stated that changes in CO_2_ concentration, temperature, and water availability can have a positive, neutral, or negative impact on disease development. Similar conclusions were presented by Hernroth and Baden (2018) regarding shellfish. They stated that a moderate increase in temperature may have a stimulating effect on antimicrobial activity and may counteract the negative effects of warming in the future. On the other hand, they added that rising seawater surface temperature and climatic events causing land runoff promotes the abundance of natural pathogen (in this case, it was *Vibrio*) and introduces enteric pathogens into coastal waters.

It can, however, be noticed that changes in the notification number in the RASFF in a short period could be also a result of changes in the European law.

### 4.1 Legal conditions of notifications in the RASFF

The first significant increase in the number of notifications in the RASFF can be observed in 2002/2003 (as mentioned before) after the Regulation (EC) n. 178/2002 came into force on 21.02.2002 and 1.01.2005 (European Parliament, & Council, 2002), and then in 2004/2005 after adopting food hygiene regulations: the Regulation (EC) n. 852/2004 (on hygiene of foodstuffs; European Parliament, & Council, 2004a), the Regulation (EC) n. 853/2004 (laying down specific hygiene rules for food of animal origin; European Parliament, & Council, 2004b), and the Regulation (EC) n. 854/2004 (laying down specific rules for organization of official controls on products of animal origin intended for human consumption; European Parliament, & Council, 2004c), all in force from 20.05.2004 and 1.01.2006, as well as the Regulation (EC) n. 882/2004 (on official controls performed to ensure verification of compliance with feed and food law, animal health and animal welfare rules), in force from 1.01.2006 and 1.07.2007 (European Parliament, & Council, 2004d).

However, in the meantime the following acts were also adopted: the Decision 2005/85/EC (imposing special conditions on the import of pistachios and certain products derived from pistachios originating in, or consigned from Iran), with the date of effect 1.02.2005 (Commission, 2005a) and 7.02.2005, not then in force; the Regulation (EC) n. 2073/2005 (on microbiological criteria for foodstuffs) in force from 1.01.2006 and 11.01.2006 (Commission, 2005b) and the Regulation (EC) n. 1881/2006 (setting maximum levels for certain contaminants in foodstuffs) in force from 9.01.2007 and 1.03.2007 (Commission, 2006). The Regulation (EC) n. 2073/2005 related to microorganisms in meat and products thereof, milk and dairy products, egg products, fishery products and vegetables, fruits, and products thereof (Commission, 2005b). In turn, the Regulation (EC) n. 1881/2006 referred to nitrate, mycotoxins, metals, and other contaminants (Commission, 2006). These law acts could contribute to a decrease in the number of notifications in 2005–2007 (particularly in the case of pathogenic microorganisms and composition); however, particularly important was managing the hazard associated with mycotoxins in pistachios from Iran (although there was a distinct increase in 2008). In contrast, the number of notifications on pathogenic microorganisms again clearly increased from 2007 to 2017.

From 2009, there were also fluctuations in the number of notifications, which could be related to the adoption of the Regulation (EC) n. 669/2009 (implementing the Regulation (EC) n. 882/2004 as regards the increased level of official controls on imports of certain feed and food of nonanimal origin) in force from 14.08.2009 and 25.01.2010 (Commission, 2009) and the Regulation (EU) n. 16/2011 (laying down implementing measures for the Rapid Alert System for Food and Feed) in force from 31.01.2011 (Commission, 2011). The Regulation (EC) n. 854/2004 and the Regulation (EC) n. 882/2004 will be repealed and replaced by the Regulation (EU) 2017/625 (on official controls and other official activities performed to ensure application of food and feed law, rules on animal health and welfare, plant health and plant protection products) with the date of effect 14.12.2019 (European Parliament & Council, 2017). However, it is already partly in force.

### 4.2 RASFF notifications reported by different authors

Only since 2011, the European Commission has included in its annual report information on the 10 most frequently reported hazard types in the RASFF in the previous year. Table 3 presented a summary of these hazards in the descending order for individual years (2010–2017), completed with hazard categories and taking into account the product category and origin country. It can be seen that the hazards are becoming more and more diverse. However, according to the Commission, notifications during this period mainly concerned aflatoxins (type of mycotoxins) in nuts and seeds from China, Iran, Turkey, and the United States. Aflatoxins have also been frequently reported in fruits and vegetables from Turkey and herbs and spices from India. *Salmonella* (pathogenic microorganism) was reported in fruits and vegetables from Bangladesh and India, as well as in poultry meat from Brazil and Poland. The Commission also noted mercury in fish from Spain and pesticide residues in fruits and vegetables from Turkey. Annual reports also indicated the migration of heavy metals (chromium and manganese) from food contact materials originated from China. Thus, the results presented in Sections 3.2.1, 3.2.8 correspond to those reported by the European Commission. However, it is worth noting that according to the Commission about 70% of notifications in each year of the period 2010–2017 were alerts for products from EU countries (European Union 2011, 2012, 2013, 2014, 2015, 2016, 2017, 2018). This is particularly worrying because the number of notifications increases from year to year. What's more, in 2017, for the first time, the number of notifications for products from Europe was greater than for products from Asia (European Union, 2018).

**Table 3:** The most frequently notified hazards in the RASFF according to the European Commission in 2010–2017

Year	Hazard category (and hazard type)	Product category (and origin country)	Reference
2010	Mycotoxins (aflatoxins)	Nuts and seeds (from Argentina, China, Iran, Turkey, the United States); herbs and spices (from India); fruits and vegetables (from Turkey)	European Union (2011)
	Genetically modified food or feed (unauthorized genetically modified)	Cereals (from China)	
	Heavy metals (mercury)	Fish (from Spain)	
	Heavy metals (migration of chromium)	Food contact materials (from China)	
2011	Mycotoxins (aflatoxins)	Nuts and seeds (from China, Turkey, Iran); feed materials (from India) Fruits and vegetables (from Turkey); herbs and spices (from India)	European Union (2012)
	Pathogenic microorganisms (*Salmonella* spp.)	Fruits and vegetables (from Bangladesh)	
	Heavy metals (migration of chromium)	Food contact materials (from China)	
	Migration (migration of formaldehyde)	Food contact materials (from China)	
	Foreign bodies (living and dead mites)	Nuts and seeds (from Ukraine)	
2012	Mycotoxins (aflatoxins)	Fruits and vegetables (from Turkey); nuts and seeds (from China); feed materials (from India)	European Union (2013)
	Migration (migration of formaldehyde)	Food contact materials (from China)	
	Heavy metals (migration of chromium)	Food contact materials (from China)	
	Pathogenic microorganisms (*Salmonella* spp.)	Fruits and vegetables (from Bangladesh)	
	Heavy metals (migration of manganese)	Food contact materials (from China)	
	Heavy metals (migration of nickel)	Food contact materials (from China)	
	Migration (migration of primary aromatic amines)	Food contact materials (from China)	
	Pesticide residues (monocrotophos)	Fruits and vegetables (from India)	
2013	Mycotoxins (aflatoxins)	Nuts and seeds (from Turkey, China); fruits and vegetables (from Turkey)	European Union (2014)
	Heavy metals (migration of chromium)	Food contact materials (from China)	
	Heavy metals (mercury)	Fish (from Spain)	
	Heavy metals (migration of manganese)	Food contact materials (from China)	
	Pathogenic microorganisms (*Salmonella* spp.)	Poultry meat (from Brazil)	
	Composition (carbon monoxide treatment)	Fish (from Spain)	
	Pathogenic microorganisms (*Salmonella Heidelberg*)	Poultry meat (from Brazil)	
	Pathogenic microorganisms (*Salmonella enteritidis*)	Poultry meat (from Poland)	
2014	Mycotoxins (aflatoxins)	Nuts and seeds (from Iran, China, Turkey); fruits and vegetables (from Turkey)	European Union (2015)
	Heavy metals (mercury)	Fish (from Spain)	
	Pathogenic microorganisms (*Salmonella* spp.)	Poultry meat (from Brazil)	
	Heavy metals (migration of chromium)	Food contact materials (from China)	
	Pathogenic microorganisms (*Listeria monocytogenes*)	Fish (from Poland)	
	Pathogenic microorganisms (norovirus)	Bivalve mollusks (from Vietnam)	
	Pathogenic microorganisms (Shiga toxin‐producing *Escherichia coli*)	Meat (from New Zealand)	
	Heavy metals (migration of manganese)	Food contact materials (from China)	
	Genetically modified food or feed (unauthorized genetically modified)	Feed additives (from China)	
	Pesticide residues (unauthorized substance dichlorvos)	Fruits and vegetables (from Nigeria)	
2015	Mycotoxins (aflatoxins)	Nuts and seeds (from China, Iran, Turkey, the United States); fruits and vegetables (from Turkey)	European Union (2016)
	Pathogenic microorganisms (*Salmonella*)	Fruits and vegetables (from India)	
	Pathogenic microorganisms (*Salmonella*)	Nuts and seeds (from India)	
	Heavy metals (mercury)	Fish (from Spain)	
	Pathogenic microorganisms (*Salmonella*)	Poultry meat (from Brazil)	
	Heavy metals (migration of chromium)	Food contact materials (from China)	
2016	Mycotoxins (aflatoxins)	Nuts and seeds (from Turkey, Iran, China, the United States, Egypt); fruits and vegetables (from Turkey); herbs and spices (from India)	European Union (2017)
	Pesticide residues	Fruits and vegetables (from Turkey)	
	Heavy metals (mercury)	Fish (from Spain)	
	Pathogenic microorganisms (*Salmonella*)	Fruits and vegetables (from India)	
2017	Pathogenic microorganisms (*Salmonella*)	Poultry meat (from Spain, Poland)	European Union (2018)
	Mycotoxins (aflatoxins)	Nuts and seeds (from China, Turkey, Iran); fruits and vegetables (from Turkey)	
	Pesticide residues	Fruits and vegetables (from Turkey)	
	Heavy metals (mercury)	Fish (from Spain)	
	Novel food (unauthorized novel food–ingredient)	Dietetic food (from the United States)	
	Pesticide residues (fipronil)	Eggs (from Italy)	

However, most authors usually only briefly mentioned RASFF notifications within one or few hazard types (or hazard categories) in particular year (or period) in the context of their research. Table 4 presented these hazards (completed with hazard category if it was not indicated) and products. Only in rare cases authors indicated also origin country. Similarly, as in the case of RASFF annual reports, the most frequently indicated notifications were aflatoxins in nuts, fruits and vegetables and herbs and spices, pathogenic microorganisms in herbs and spices, meat, poultry, and seafood, pesticide residues in fruits and vegetables, and herbs and spices, as well as residues of veterinary medicinal products and cadmium in seafood. However, attention was also paid to other hazards, for example, parasitic infestation and industrial contaminants in seafood; food additives and flavorings in fruits and vegetables, herbs, and spices; and dietetic food, nuts, and seafood.

**Table 4:** Hazards reported in the RASFF in 1996–2018 indicating by different authors

Year(s)	Hazard category (and hazard type)	Product (and origin country)	Reference
1996–2018	Composition	Cereals and bakery products	Kowalska, Soon, and Manning (2018)
1998–2011	Pathogenic microorganisms, residues of veterinary medicinal products (antibiotic and other products), heavy metals, food additives and flavorings	Pangasius, shrimp, swordfish, tuna (from Vietnam)	Little et al. (2012)
2000–2010	Mycotoxins (aflatoxins)	Nuts, nut products and seeds (mainly pistachios)	García‐Cela, Ramos, Sanchis, and Marín (2012)
2000–2017	Mycotoxins (aflatoxins)	Nutmeg (from Indonesia)	Wahidin and Purnhagen (2018)
2001–2009	Residues of veterinary medicinal products (chloramphenicol)	Shrimp (from Indonesia)	Wahidin and Purnhagen (2018)
2002–2011	Residues of veterinary medicinal products (malachite green and leucomalachite green)	Fish and fish products (from China, Indonesia, Malaysia, Denmark, Germany, the Netherlands, Sweden, United Kingdom)	Bilandžić, Varenina, Solomun Kolanovic, Oraic, and Zrncic, (2012)
2002–2012	Mycotoxins (aflatoxins)	Capsicums (from India)	Golge, Hepsag, and Kabak (2013)
2002–2013	Residues of veterinary medicinal products (malachite green and leucomalachite green)	Fish and seafood products (from Vietnam, Thailand, Indonesia, China, and European countries)	Fallah and Barani (2014)
2002–2014	Mycotoxins (aflatoxins)	Dried figs, hazelnuts, and pistachios (from Turkey)	Kabak (2016)
2003–2006	Heavy metals (cadmium)	Fishery products	Figueroa (2008)
2003–2007	Mycotoxins (aflatoxins, ochratoxin A)	Dried fruits (from Turkey)	Bircan (2009)
2003–2009	Food additives and flavorings (Sudan I, Sudan IV)	Palm oil (from African countries)	Rebane, Leito, Yurchenko, and Herodes (2010)
2003–2010	Pathogenic microorganisms (*Salmonella*), mycotoxins (aflatoxins), allergens, food additives and flavorings, radiation	Dietetic food, food supplements and fortified foods	Petróczi, Taylor, and Naughton (2011)
2003–2012	Residues of veterinary medicinal products (nitrofuran metabolites)	Crustaceans and associated products	Douny et al. (2013)
2003–2014	Residues of veterinary medicinal products (chloramphenicol)	Dairy products	Śniegocki, Gbylik‐Sikorska, and Posyniak (2015)
2003–2016	Pathogenic microorganisms (*Salmonella*)	Herbs and spices	Lins (2018a, 2018b)
2004	Pathogenic microorganisms, pesticide residues	Pangasius (from Vietnam)	Jespersen, Kelling, Ponte, and Kruijssen (2014)
2004–2006	Residues of veterinary medicinal products (nitrofurazone)	Aquaculture products (from Asian countries)	Hassan et al. (2013)
2004–2009	Mycotoxins (aflatoxins)	Nuts and dried figs (from Turkey)	Imperato, Campone, Piccinelli, Veneziano, and Rastrelli (2011)
2004–2009	Pathogenic microorganisms	Composite products	Stella et al. (2013)
2004–2014	Pathogenic microorganisms (*Listeria*)	Cooked ham	Zwietering, Jacxsens, Membré, Nauta, and Peterz (2016)
2005	Mycotoxins (aflatoxins)	Pistachio nuts (from Iran)	Ariño et al. (2009, Molyneux, Mahoney, Kim, and Campbell (2007)
2005–2006	Pathogenic microorganisms	Fresh herbs and spices	Elviss et al. (2009)
2005–2006	Food additives and flavorings (Sudan I, Sudan IV)	Spices—chili, seasonings, spice mixtures	Vera, Ruisánchez, and Callao (2018)
2005–2014	Mycotoxins, pesticide residues, pathogenic microorganisms, composition	Herbs and spices—mainly chili and curry (from India, Thailand)	Bouzembrak, Camenzuli, Janssen, and Van Der Fels‐Klerx (2018)
2005–2014	Pathogenic microorganisms (*Salmonella*)	Chocolate	Zwietering et al. (2016)
2007	Residues of veterinary medicinal products	Meat other than poultry	Andrée, Jira, Schwind, Wagner, and Schwägele (2010)
2007–2009	Heavy metals (cadmium)	Crabs (from France, Ireland, United Kingdom)	Noël et al. (2011)
2007–2014	Composition, novel food (unauthorized ingredients), residues of veterinary medicinal products (undeclared medicinal drugs)	Dietetic food, food supplements, and fortified foods	Da Justa Neves and Caldas (2015)
2008	Mycotoxins (aflatoxins)	Nuts, nut product and seeds	Ding, Li, Bai, and Zhou (2012)
2008–2010	Mycotoxins	Nuts, dried fruits and spices	Van De Perre, Jacxsens, Lachat, El Tahan, and De Meulenaer (2015)
2008–2011	Pesticide residues, mycotoxins, pathogenic microorganisms, food additives and flavorings	Fruits and vegetables, herbs and spices, nuts, nut products, and seeds	Uyttendaele, Jacxsens, and Van Boxstael (2014)
2008–2011	Pesticide residues, mycotoxins, pathogenic microorganisms, food additives and flavorings	Fruits and vegetables, herbs, and spices	Van Boxstael et al. (2013)
2008–2012	Adulteration/ fraud	Fish and fish products, meat and meat products (including poultry), nuts, nut products, and seeds	Tähkäpää, Maijala, Korkeala, and Nevas (2015)
2008–2013	Industrial contaminants (semicarbazide), residues of veterinary medicinal products (furazolidone)	Aquaculture (from Bangladesh, China, India)	Points, Burns, and Walker (2015)
2009	Mycotoxins (aflatoxins)	Peanuts (from Argentina, China, USA, Brazil, Egypt, South Africa), Pistachios (from Iran, Turkey, USA), Hazelnuts (from Turkey)	Rodrigues, Venâncio, and Lima (2012)
2009	Mycotoxins (aflatoxins)	Hazelnuts, pistachios from Turkey	Kabak (2012)
2009	Pathogenic microorganisms (*Salmonella*, *Campylobacter*, *Listeria*)	Poultry meat products	Lavelli (2013)
2009	Residues of veterinary medicinal products (nitrofuran metabolites)	Crustaceans	Vidaček (2014)
2009	Pathogenic microorganisms (*Salmonella*)	Fish, bivalve mollusks, cephalopods, crustaceans	Amagliani, Brandi, and Schiavano (2012)
2009–2013	Parasitic infestation (Anisakis)	Fish	D'Amico et al. (2014)
2010	Pathogenic microorganisms (*Escherichia coli*)	Bivalve mollusks	Vidaček (2014)
2010	Residues of veterinary medicinal products (antibiotics)	Honey, shrimp	Van Asselt, Van der Spiegel, Noordam, Pikkemaat, and Van der Fels‐Klerx (2013)
2010–2011	Mycotoxins	Spices—mainly capsicums and nutmeg	Ozbey and Kabak (2012)
2010–2011	Mycotoxins (aflatoxins)	Dried chestnuts, flours, and flakes (from Italy)	Pietri, Rastelli, Mulazzi, and Bertuzii (2012)
2010–2016	Parasitic infestation (Anisakis)	Fish and fishery products (from Spain)	Bao et al. (2017)
2010–2016	Parasitic infestation (anisakid larvae)	Fishery products	Guardone et al. (2018)
2011	Mycotoxins (aflatoxins)	Cereals and bakery products	Cheli, Battaglia, Gallo, and Dell'Orto (2014)
2011	Mycotoxins (aflatoxins)	Nuts	Clavel and Brabet (2013)
2011	Mycotoxins (aflatoxins)	Nuts, nut products and seeds, fruits and vegetables, herbs, and spices	Johannessen and Cudjoe (2014)
2011	Pathogenic microorganisms	Shellfish	Anacleto, Barrento, Nunes, Rosa, and Marques (2014)
2011–2013	Chemical contaminants (chemical residues), allergens, food additives and flavorings (undeclared substances), heavy metals (mercury), adulteration/ fraud (fraudulent health certificates)	Fish and fish products	He (2015)
2011–2017	Heavy metals (mercury)	Fish and fish products (from Spain)	Giusti et al. (2019)
2012	Pesticide residues	Fresh pepper (from Turkey)	Engelbert, Bektasoglu, and Brockmeier (2014)
2012–2013	Parasitic infestation (anisakid larvae)	Fish	Robertson, Sprong, Ortega, Giessen, and Fayer (2014)
2012–2016	Pesticide residues (acaricide, insecticides)	Black and green tea	Cladière, Delaporte, Le Roux, and Camel (2018)
2013	Pathogenic microorganisms, mycotoxins, pesticide residues	Meat products, fruits, and vegetables	Brandão, Liébana, and Pividori (2015)
2013	Heavy metals, residues of veterinary medicinal products	Fishery products (from China)	Xiong et al. (2016)
2014	Pathogenic microorganisms (noroviruses)	Bivalve mollusks, fruits	Bosch, Pintó, and Guix (2016)
2014–2015	Mycotoxins (aflatoxins)	Peanuts	Granados‐Chinchilla et al. (2017)
2015	Mycotoxins (aflatoxins)	Nuts and nut products	Cunha Sá and Fernandes (2018)

Only some authors presented a comprehensive approach to the development of notifications within the RASFF. The detailed analysis of the RASFF notifications within different hazard and product categories was carried out by Kleter Prandini Filippi and Marvin (2009) for 2003–2007. They first of all paid attention to chemical hazards, that is, dyes, heavy metals, drug residues, allergens, and pesticides in seafood, spices, condiments, fruits, vegetables, and utensils. They also indicated microbiological hazards, mainly *Salmonella* spp., *Listeria monocytogenes,* and *Escherichia coli* in seafood, meat, poultry, spices, and condiments. However, the most important hazard in this period was mycotoxins (aflatoxins) in nuts and fruits. It was also noticed by Van Asselt, Banach, and Van Der Fels‐Klerx (2018), who indicated aflatoxins (also dyes) as one of the reasons for the RASFF notifications for spices and herbs (chili/paprika powder, curry, nutmeg, pepper, basil) in 2004–2014. In turn, in 2008–2015 notifications in the RASFF were related mainly to pathogenic microorganisms in meat and poultry, heavy metals in fish, pesticide residues in fruits and vegetables, and mycotoxins in nuts (Pigłowski, 2017). Thus, hazards involved the similar product categories. Nevertheless, significant changes in time in the RASFF notifications on mycotoxins (Figure 11) were noticed by Ariño et al. (2009); Dini et al. (2013); Freitas‐Silva and Venâncio (2011); Georgiadou, Dimou, and Yanniotis (2012); Marín, Ramos, and Sanchis (2012); Marín, Ramos, Cano‐Sancho, and Sanchis (2013); Taylor, Petróczi, Nepusz, and Naughton (2013); and Wiig and Kolstad (2005). They were a result of changes in the European law (pistachios from Iran) as mentioned before, and this problem has been largely resolved.

There are also some works concerning the RASFF notifications in greater detail within particular hazard or product categories. Pigłowski (2019), relating to pathogenic microorganisms, noticed that in 1980–2017, more frequently notified were *Salmonella* sp., *Listeria*, *Escherichia,* and *Vibrio* in products of animal origin: meat, poultry, milk, and seafood (fish, crustaceans and mollusks). D'Amico et al. (2018) also indicated pathogenic microorganisms among two main reasons for the RASFF notifications in bivalve mollusks in 2011–2015. This kind of hazard concerned also products of nonanimal origin: fruits, vegetables, herbs, spices, and nuts (Pigłowski, 2019). However, it is important that the number of notifications regarding *Salmonella* in poultry sharply increased in 2017 (Figure 11) and was related to fraud of certification of poultry meat from Brazil. This resulted in the removal of authorization for several Brazilian operators to export to the European Union. Notifications on *Salmonella* concerned also poultry from Poland (European Union, 2018).

According to Pigłowski (2018), notifications on heavy metals (mercury, cadmium, chromium, lead, arsenic, and nickel) in the RASFF in 1980–2016 related mainly to fish and food contact materials, then to fruits and vegetables, seafood, and dietetic food. They were reported by Italy, Spain, Germany, and France and originated mainly from China and Spain. Similar elements (cadmium, mercury, and lead) were also indicated as a cause of the notifications in 2003–2007 by Kleter et al. (2009). D'Amico et al. (2018) indicated heavy metals as the main reason for the RASFF notifications in seafood (fish and cephalopods) in 2011–2015. Similarly, they noticed that seafood was notified mainly by Italy and Spain and originated from these two countries, followed by Vietnam and Morocco. Both D'Amico et al. (2018) and Pigłowski (2019) pointed out that the basis for notifications on heavy metals was mainly official controls and border checks.

Recently, RASFF notification analyses for other hazard categories or product categories apart from the presented ones have also been conducted. Czepielewska, Makarewicz‐Wujec, Różewski, Wojtasik, and Kozłowska‐Wojciechowska (2018) drew attention to a significantly increasing number of notifications regarding unauthorized composition in dietetic food, food supplements, and fortified foods in 2003– 2016. In turn, Djekic, Jankovic, and Rajkovic (2017) noticed that in 1998–2015, most notifications related to foreign bodies coming from Eastern Europe. These were mainly: pests, glass, and metals in fruits and vegetables, nuts, confectionary, and bakery products.

Italy, Germany, United Kingdom and Spain were the countries, which most frequently notified products in the RASFF in 2000–2009 (Petróczi, Taylor, Nepusz, & Naughton, 2010), which was also confirmed for shorter period (2003–2007) by Taylor et al. (2013) and also for 2008–2017 by Pigłowski (2017).

Similar hazards as notified in the RASFF were also reported in other institutional systems. In the report of the Reportable Food Registry (RFR) for 2009–2014, *Salmonella*, *Listeria monocytogenes,* and undeclared allergens were indicated as the most frequently reported food hazards (Food & Drug Administration, 2016). In turn, according to the International Food Safety Authorities Network (INFOSAN) in 2011–2017, the main biological hazards were *Salmonella enterica* spp., *Clostridium* spp., *Escherichia coli,* and *Listeria monocytogenes,* and chemical hazards were heavy metals, aflatoxins, and methanol (Food and Agriculture of Organization of the United Nations, & World Health Organization, 2016; World Health Organization, & Food and Agriculture Organization of the United Nations, 2018).

### 4.3 Remarks on the RASFF database

An undoubted advantage of the RASFF database is its public availability. This enables authorities, scientists, consumers, and other interested parties to observe the most frequently reported hazards and track changing trends. For example, the RASFF enables pay attention to increasing (in the recent years) the number of notifications in such hazard categories, which may become a significant problem in the future, that is, adulteration/fraud, allergens, and novel food. However, few shortcomings can be identified. This is mainly due to the ambiguity, inaccuracy of data, or even the lack of it (especially in the case of notifications regarding earlier years of operation of the RASFF). The removal of these disadvantages could significantly increase the affordability of the RASFF database for the users.

There are currently three product types that can be notified in the RASFF, that is, food, feed, and food contact material. The name of the last product type can be misleading (and should be removed from the database), because there is also a very similar product category, that is, “food contact materials.” The same name of the hazard category and product category may also be misleading, that is “food additives and flavorings” (these names should be supplemented with information whether they related to product or hazard). In addition, there are categories in the RASFF database that are already obsolete, for example, “farmed crustaceans and products thereof,” “farmed fish and products thereof (other than crustaceans and mollusks),” “mollusks and products thereof,” “wild‐caught crustaceans and products thereof,” and “wild‐caught fish and products thereof (other than crustaceans and mollusks)”. The names of these product categories should be reclassified as currently in force.

In the notification list, which is exported from the RASFF database to Excel, there is no information about the hazard category, because one notification can concern several hazard categories, as already mentioned before. In turn, information about a specific hazard is included in the notification list in the “subject” column. The lack of information about the hazard category means that it is necessary to export each category separately. If a given notification would refer to several hazard categories, each of them should be placed in a separate column. In turn, the “subject” column should be divided into several separate columns, for example, “hazard type,” “detected value,” “reference value,” and “other remarks,” next to the given hazard category. Besides, most RASFF hazard categories (including those that have been examined) relate to specific contaminants in food. However, some categories relate to activities (or lack of activities) that may only indirectly affect food safety. These are, for example, poor or insufficient controls, adulteration/fraud, labeling absent/incomplete/incorrect or packaging defective/incorrect. They could be classified into the existing hazard category, that is, “Not determined/other” or the new “Other hazards.”

The notification list also does not contain directly information about the origin country (this information is included in the column “countries concerned”). It would be clearer to divide this column into several separate columns, for example, “country of raw materials origin,” “production country,” and “distribution country.” The missing data on countries concerned, but also on notification basis, distribution status and action taken should be completed. Other difficulties in data interpretation were similar names, which were adopted in the RASFF database. Both in the case of distribution status and in the case of action taken, the phrase “product already consumed” is used. In the case of action taken, similar phrases “informing recipients” and “informing recipient(s)” are used (these phrases should be removed, changed, or more accurately described). Additionally, risk decision is rarely made, even in the case of alerts, that is, it is most often classified as “undecided.” The risk decision should be clearly indicated, that is, “serious” or “not serious.”

Another type of difficulties in processing data obtained from RASFF was the change or addition of new hazard categories or notification types. Alerts were reported from 1979, information notifications in 1989–2011, information for attention from 2011, information for follow‐up from 2010, and border rejections from 2008. However, these changes were related to the development of RASFF. Other additional information (name, graphic symbol, and address) regarding economic operators (manufacturer, exporter, distributor), related to the notified product, as well as photographs of this product also could be included in the RASFF database. This kind of information is included in the RAPEX (Rapid Alert System for Dangerous Non‐food Products). Unification of trainings for persons to be responsible for reporting notifications to the RASFF in individual member countries may also be considered. All proposed changes would improve the traceability of the product and could contribute to increasing food safety in the European Union.

## 5 CONCLUSIONS

Food was the most frequently product type notified in the Rapid Alert System for Food and Feed (RASFF) in 1979–2017 (89.5% of all notifications). The number of notifications exceeded the mean value in the case of mycotoxins (23.0% of notifications within food), pathogenic microorganisms (18.2%), pesticide residues (8.7%), heavy metals (6.0%), composition (5.8%), food additives and flavorings (5.6%), residues of veterinary medicinal products (4.4%), and foreign bodies (3.7%). However, the main problems were mycotoxins in nuts, pathogenic microorganisms in poultry meat and fish, pesticide residues in fruits and vegetables, and heavy metals in fish.

Significant fluctuations in notifications began in 2003 and related to changes in law. Products were notified mainly by Italy, Germany, and United Kingdom, and also by Spain, the Netherlands, France, Denmark, Belgium, and Poland. Diversified notification activity probably depended on volume of production and the food flow, mainly related to import and re‐export, as well as preparation of inspection bodies. Notified products originated mainly from Asian countries (China, Turkey, India, Iran, Thailand, and Vietnam), EU countries (Spain, Germany, Italy, and France), and also the United States and Brazil. The notifications of products from European countries are particularly worrisome due to a possibility of free movement of food within the common market. However, the notification basis was mainly: border control, after which the consignment was detained, and official control on the market and notification type were: information, border rejection, and alert. Products were usually not distributed or not placed on the market, distribution status was also often not specified, or distribution was possible, also to other countries. Risk decision on notified products was most often not made. Products were redispatched, import was not authorized, and products were also withdrawn from the market or destroyed.

The RASFF database can be considered as a useful tool for tracking changes in notifications within the main hazards. However, in this base the data provided are often ambiguous (food contact material as product type and food contact materials as a product category, food additives and flavorings both as hazard category and product category), inaccurate (sort of product category, production country, origin country not given), or missing (in the case of the notification basis, origin country, distribution status, and action taken), particularly for earlier years. It significantly impedes a possibility of conducting research in the field of traceability. Nevertheless, the RASFF can significantly contribute to ensuring food safety in the European market.

However, the food policy of the European Union should pay more attention to the hazards that occur in products from EU countries, because more than half of the RASFF alert notifications concern products freely moved within the common market. Hazards in products that come from outside the EU had in fact been largely overrun in the earlier years of the RASFF by the introduction of border rejections. It is also worth noting that what was supposed to help becomes a hazard, which is why pesticide residues, drug residues, or additives are present in food. Therefore, the law regarding the application of these measures should be significantly tightened, for example, by limiting sales, increasing the grace period and official controls. In addition, more sustainable agriculture should be sought, that is, extensively or ecologically managed farms should be promoted, for example, by increasing subsidies for them. At the same time, the functioning of large‐scale farms conducting intensive farming should be limited.

## CONFLICT OF INTEREST

The author declares no conflict of interest.

## ETHICAL STATEMENT

This study does not involve any human nor animal testing.
