# Supplementary material for: Food hazards on the European Union market: The data analysis of the Rapid Alert System for Food and Feed
Source: Food Sci Nutr. 2020 Feb 11;8(3):1603–27. doi: 10.1002/fsn3.1448 (PMC7063371; doi:10.1002/fsn3.1448)
Supplement: Supplementary file 1 [file FSN3-8-1603-s001.pdf]

Supplementary Information

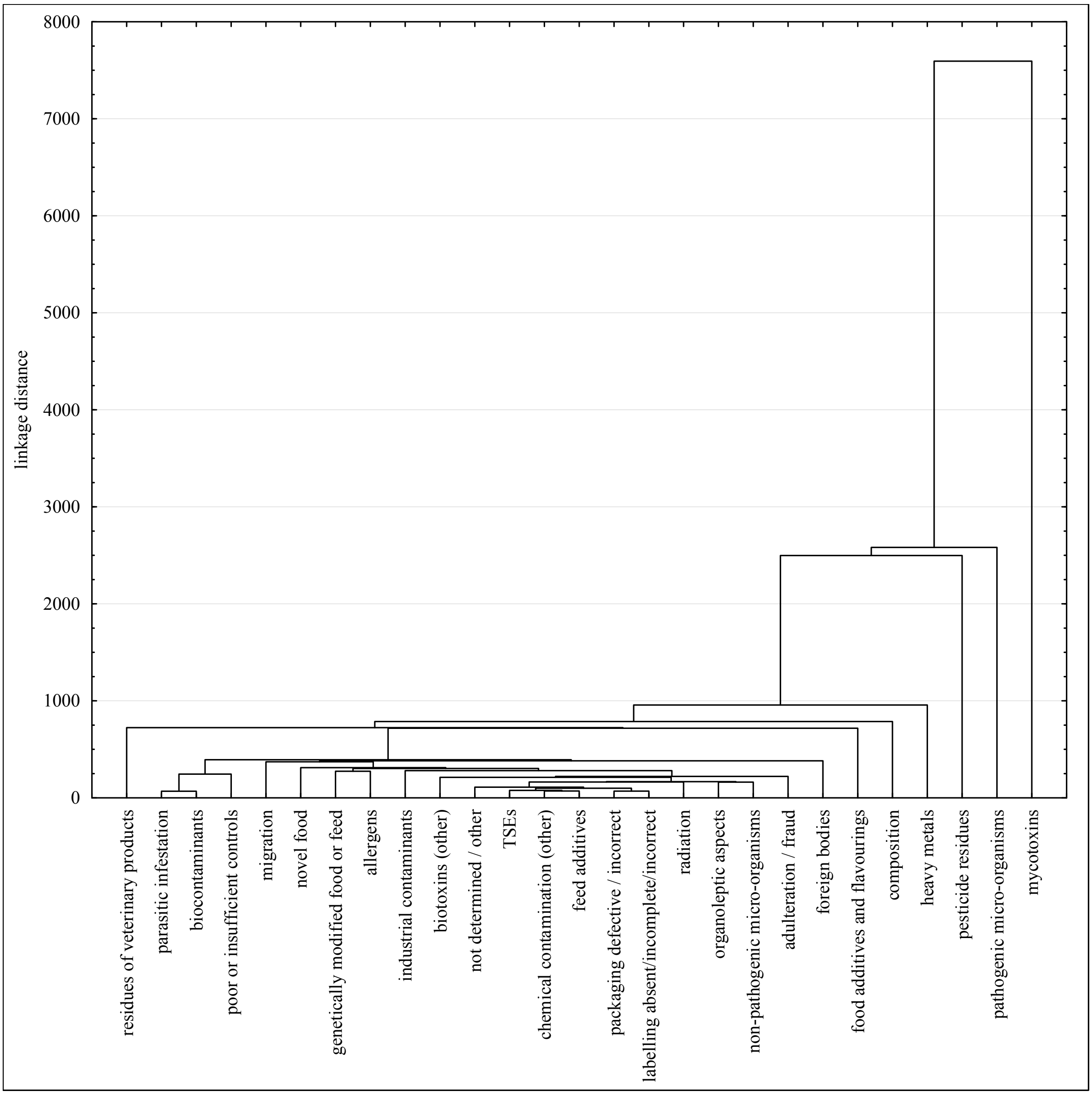

FIGURE S1 Similarities between notifications within product categories and hazard categories.

residues of veterinary products – residues of veterinary medicinal products
TSEs – transmissible spongiform encephalopathies

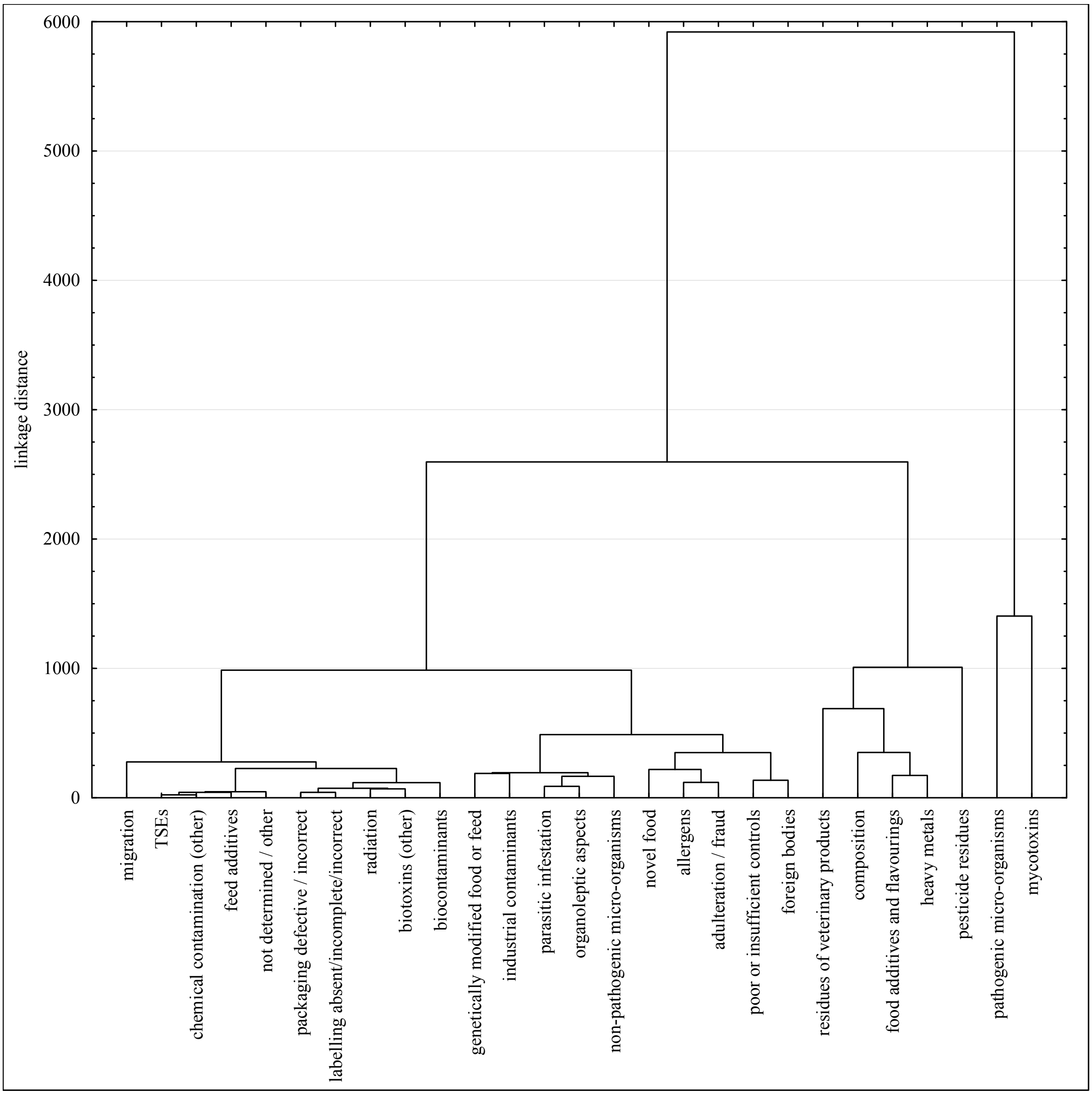

FIGURE S2 Similarities between notifications within years and hazard categories.

residues of veterinary products – residues of veterinary medicinal products
TSEs – transmissible spongiform encephalopathies

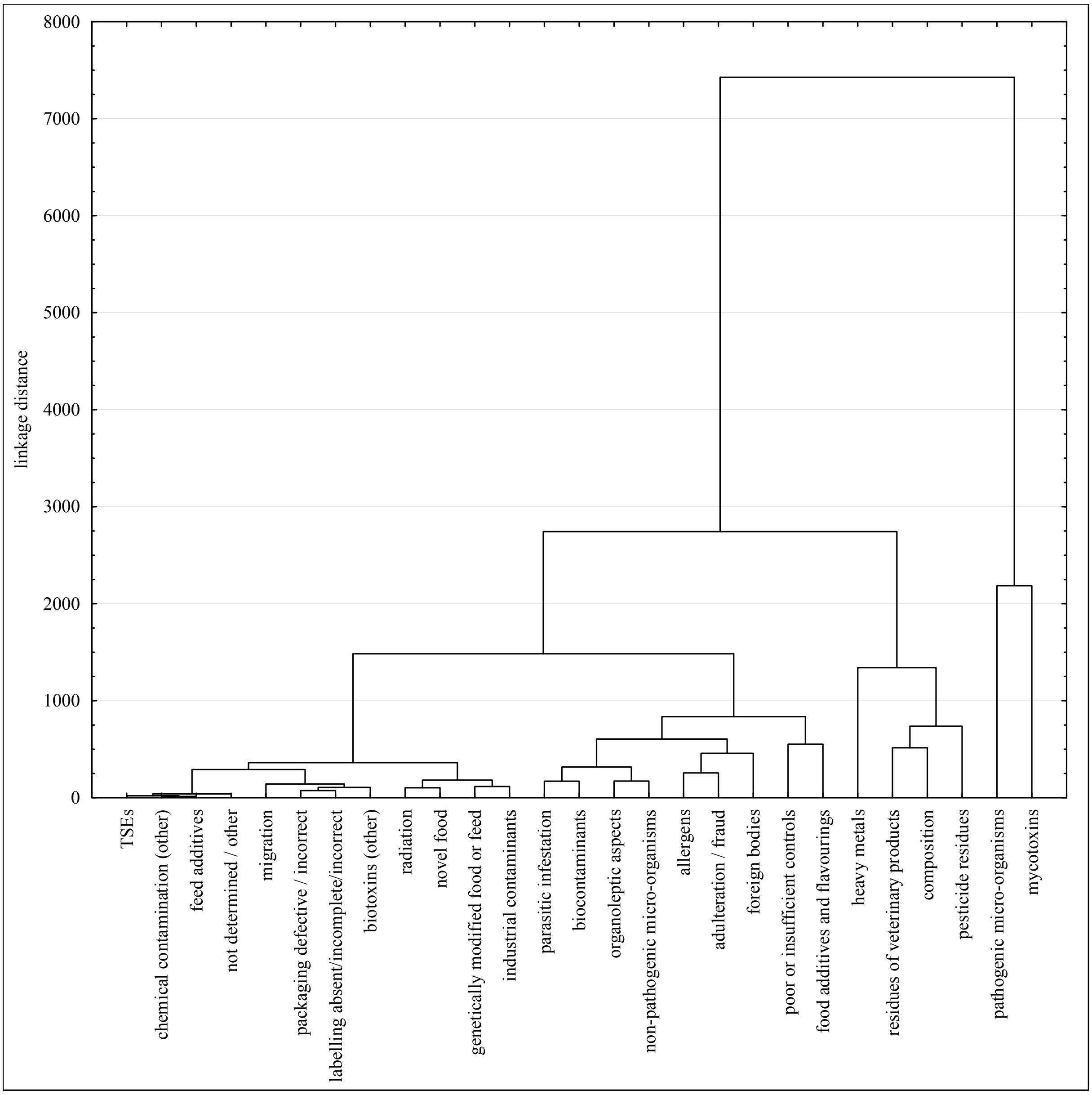

FIGURE S3 Similarities between notifications within notifying countries and hazard categories.

residues of veterinary products – residues of veterinary medicinal products
TSEs – transmissible spongiform encephalopathies

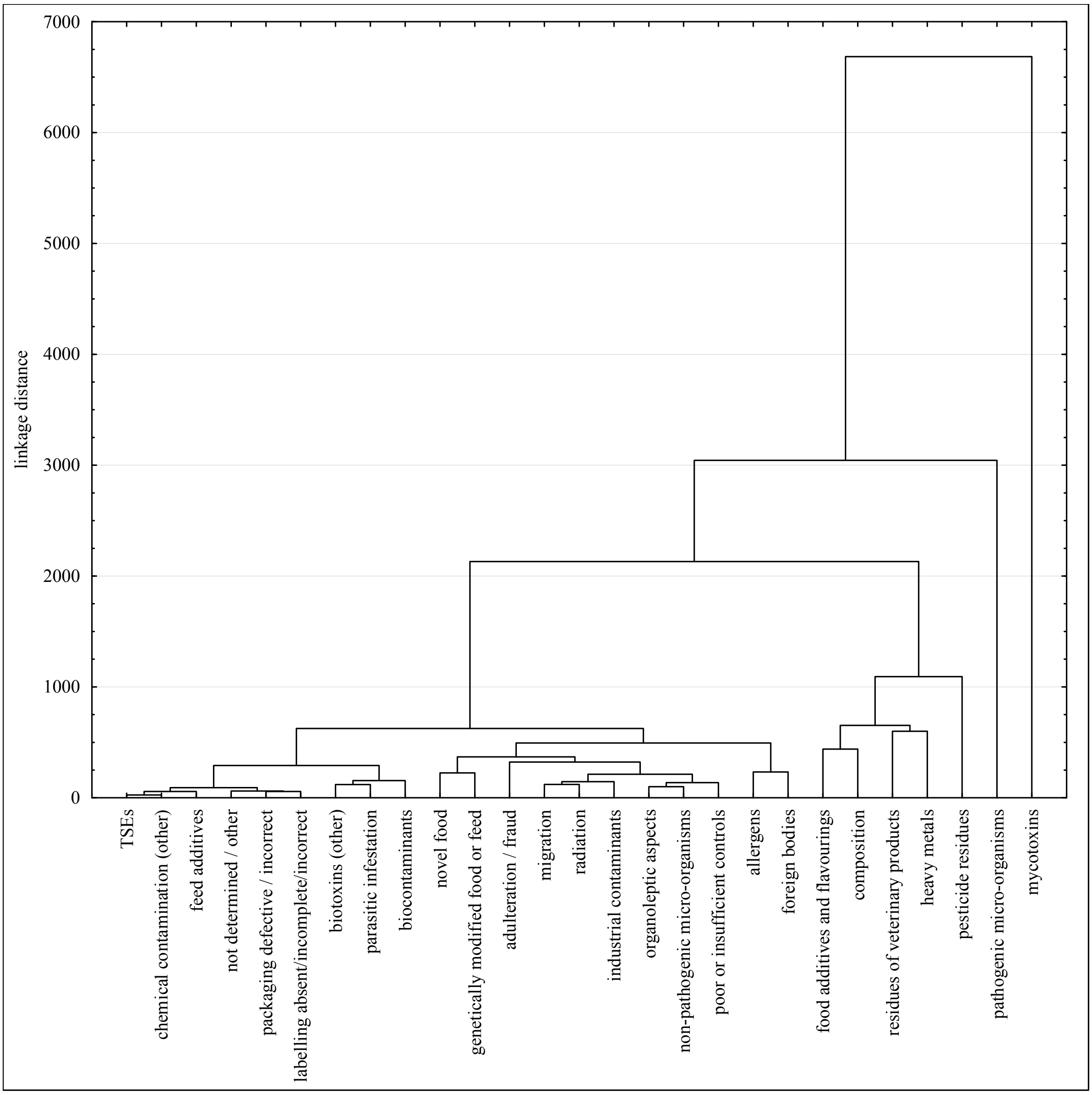

FIGURE S4 Similarities between notifications within origin countries and hazard categories.

residues of veterinary products – residues of veterinary medicinal products
TSEs – transmissible spongiform encephalopathies

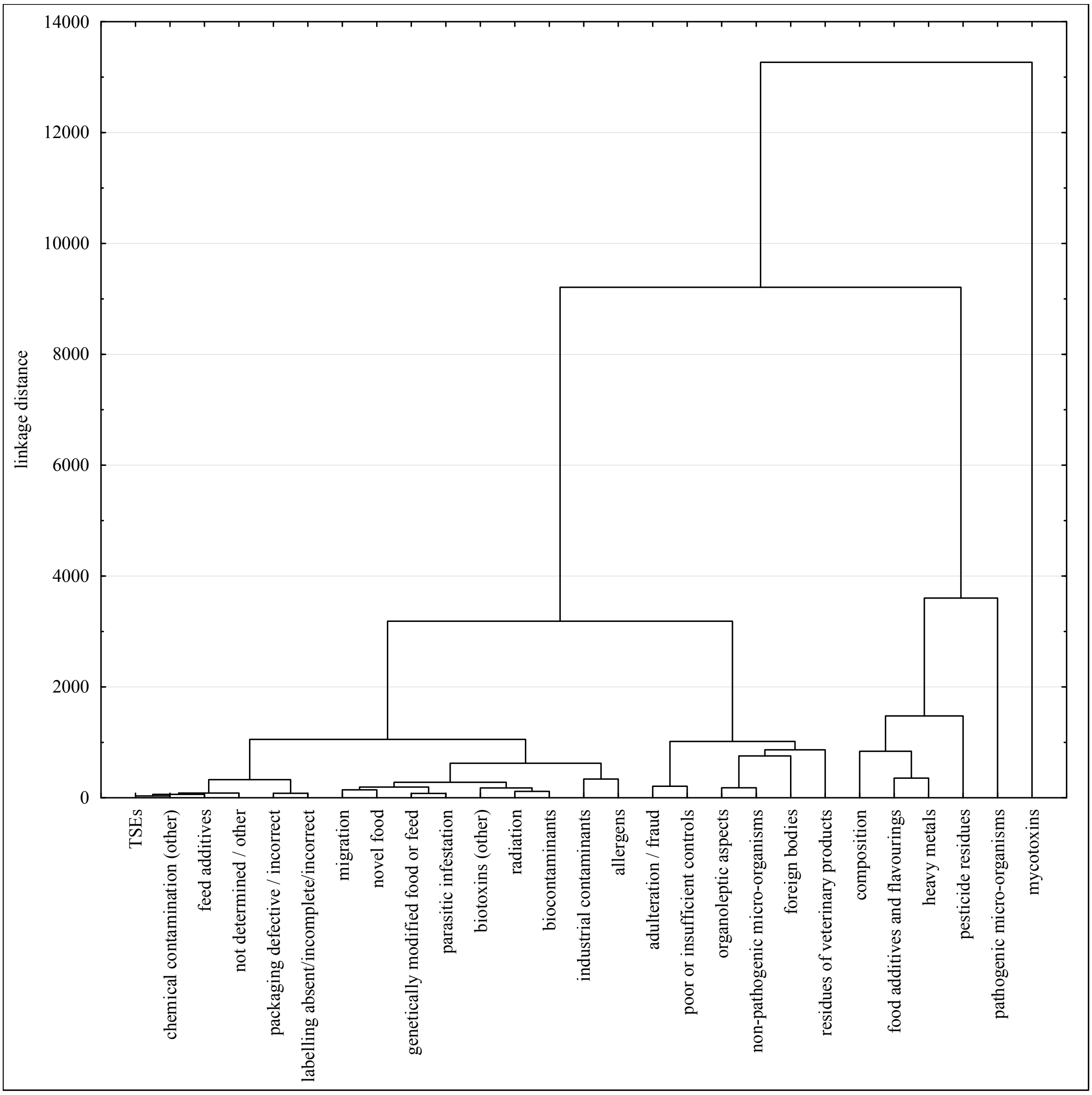

FIGURE S5 Similarities between notifications within notification basis and hazard categories.

residues of veterinary products – residues of veterinary medicinal products
TSEs – transmissible spongiform encephalopathies

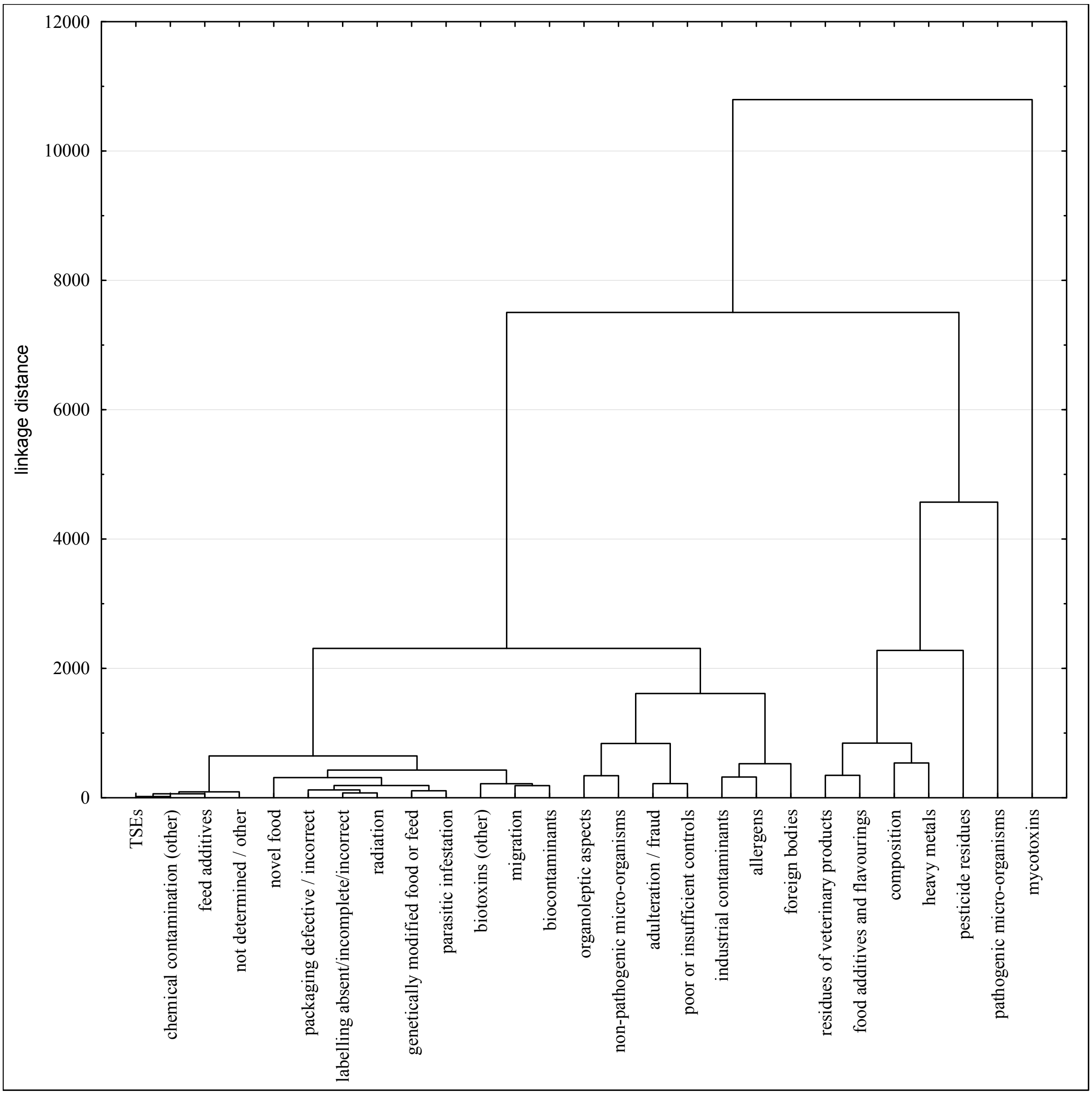

FIGURE S6 Similarities between notifications within notification type and hazard categories.

residues of veterinary products – residues of veterinary medicinal products
TSEs – transmissible spongiform encephalopathies

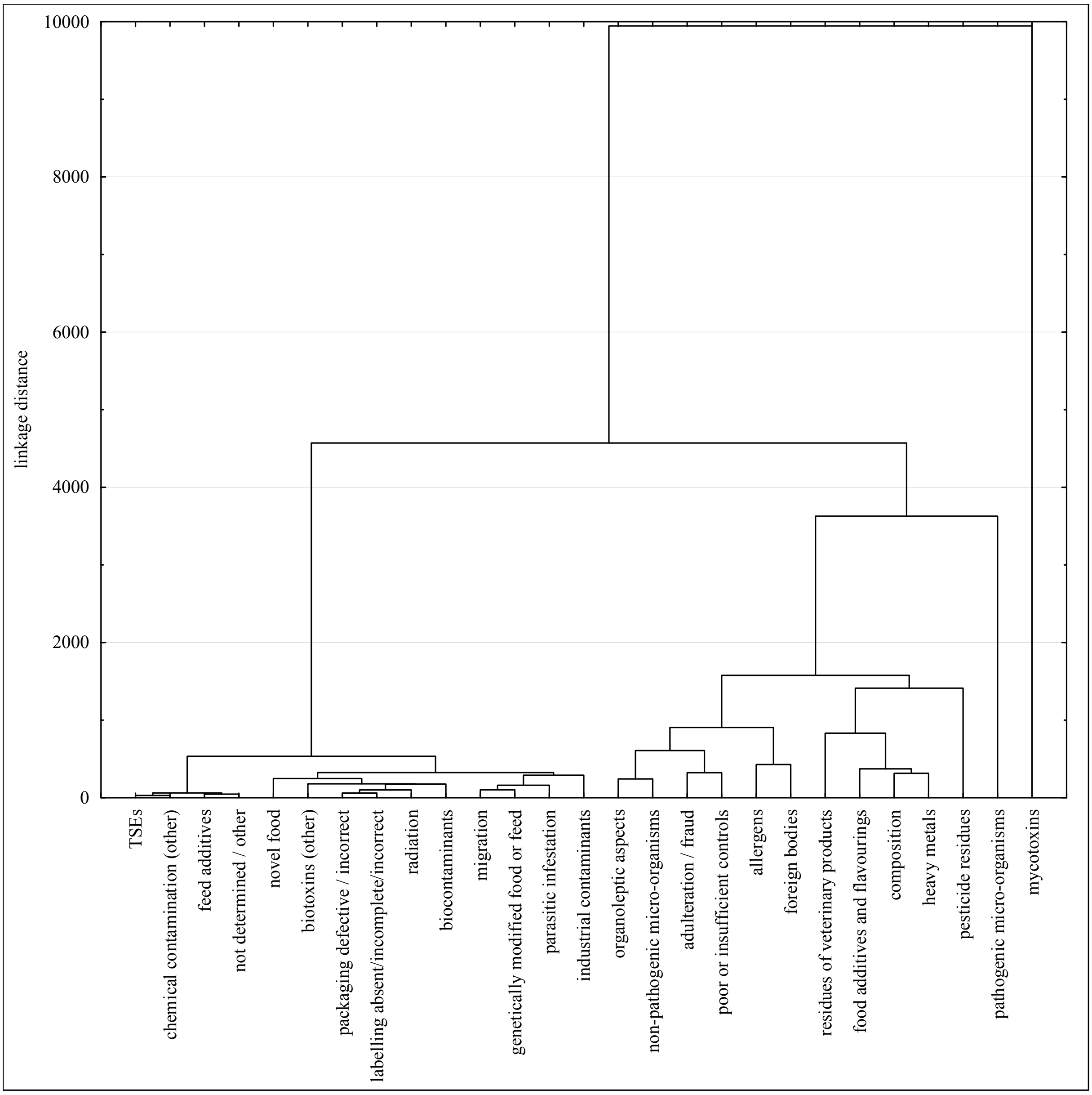

FIGURE S7 Similarities between notifications within distribution status and hazard categories.

residues of veterinary products – residues of veterinary medicinal products
TSEs – transmissible spongiform encephalopathies

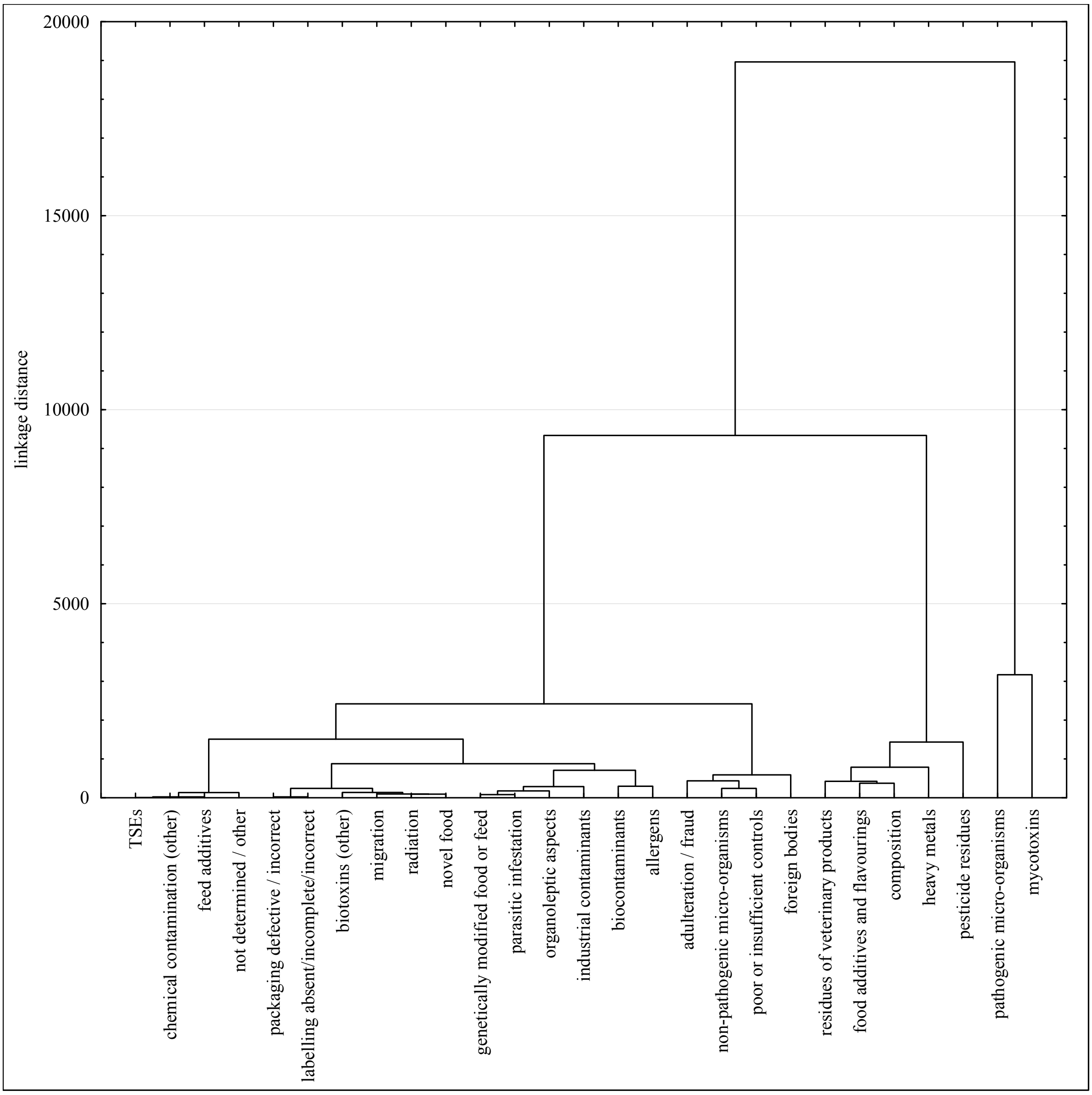

FIGURE S8 Similarities between notifications within risk decision and hazard categories.

residues of veterinary products – residues of veterinary medicinal products
TSEs – transmissible spongiform encephalopathies

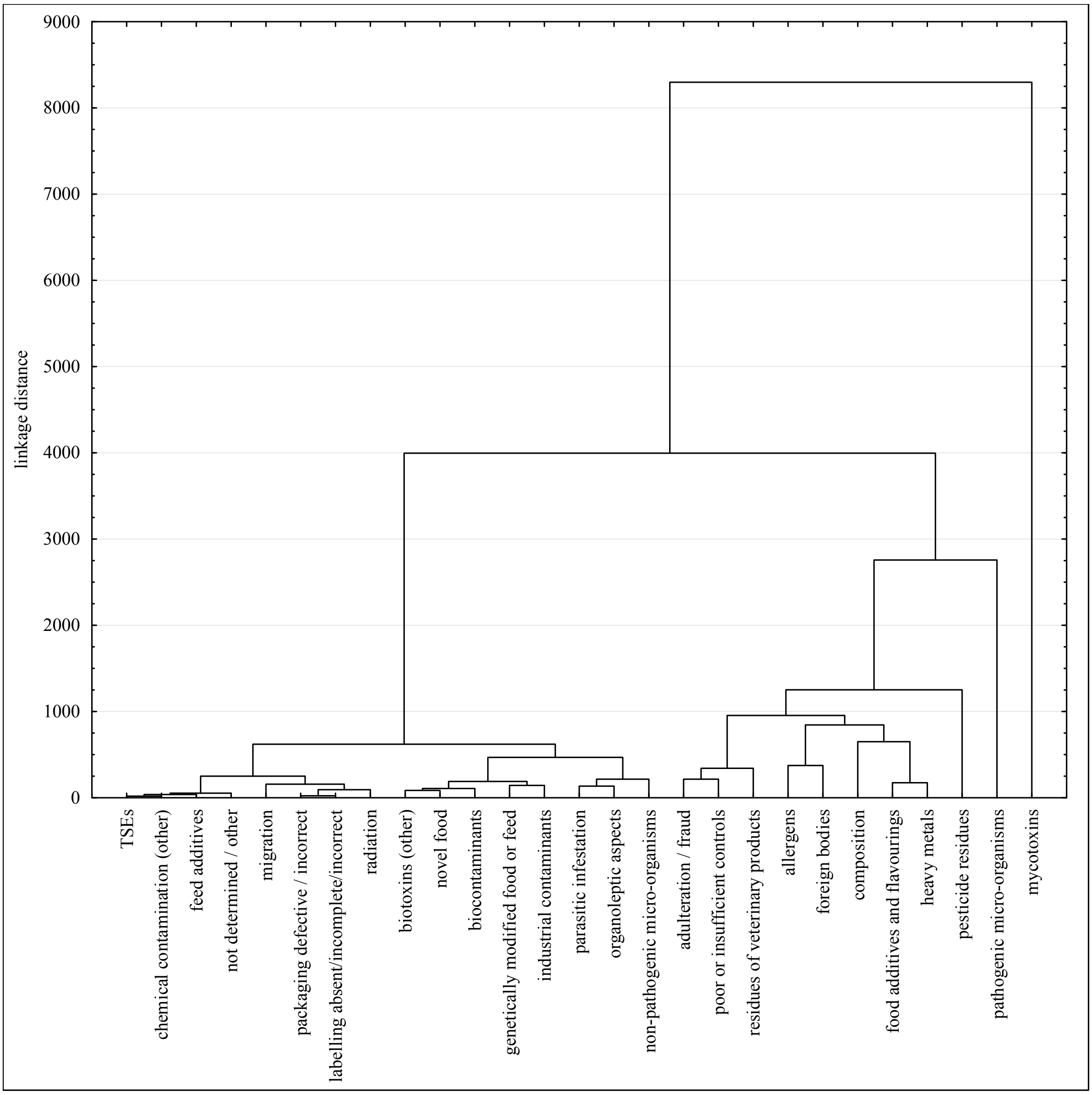

FIGURE S9 Similarities between notifications within action taken and hazard categories.

residues of veterinary products – residues of veterinary medicinal products
TSEs – transmissible spongiform encephalopathies

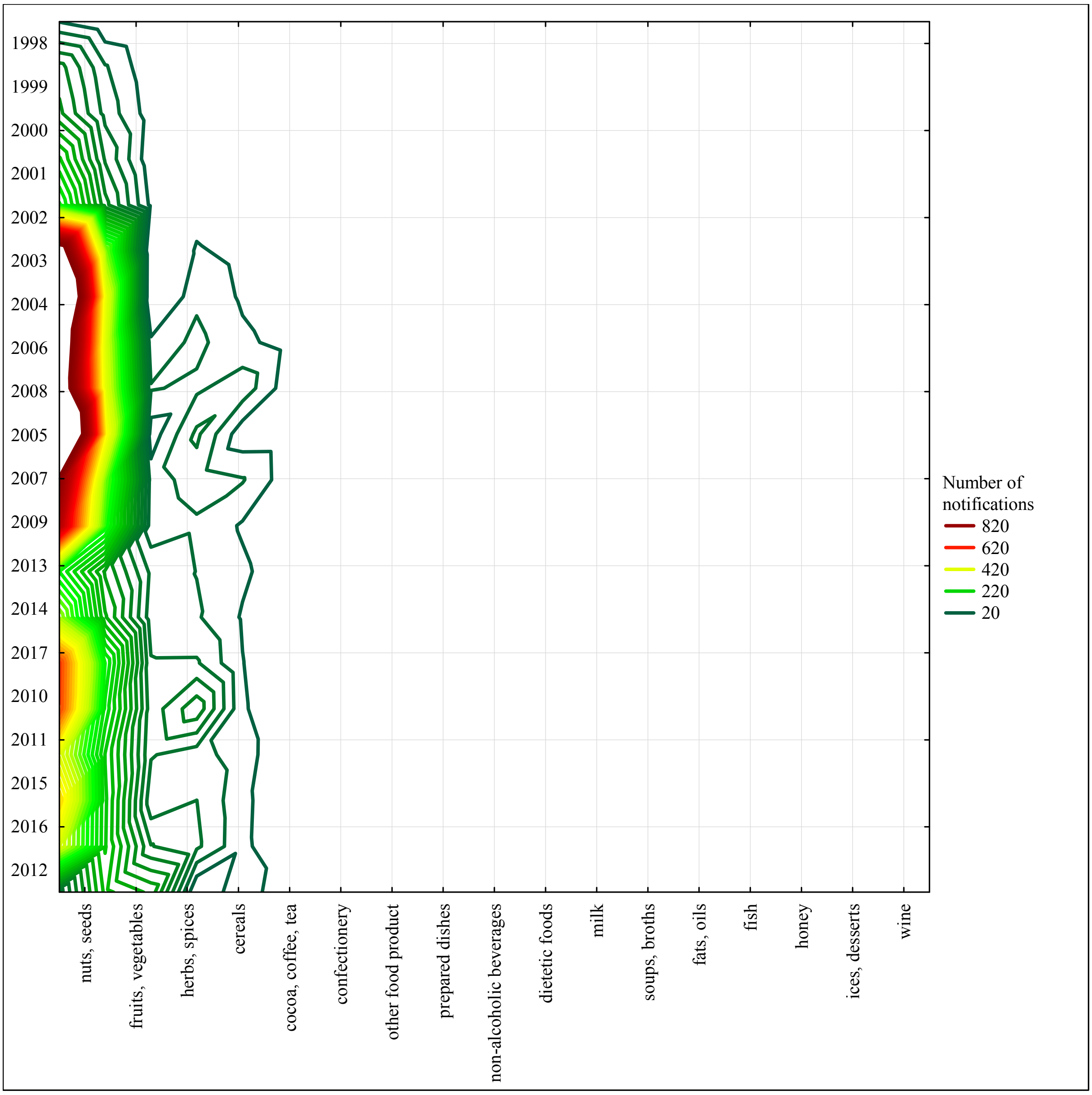

cereals – cereals and bakery products
cocoa, coffee, tea – cocoa and cocoa preparations, coffee and tea
dietetic foods – dietetic foods, food supplements, fortified foods
fats, oils – fats and oils
fish – fish and fish products
fruits, vegetables – fruits and vegetables
herbs, spices – herbs and spices
honey – honey and royal jelly
ices, desserts – ices and desserts
milk – milk and milk products
nuts, seeds – nuts, nut products and seeds
other food product – other food product / mixed
prepared dishes – prepared dishes and snacks
soups, broths – soups, broths, sauces and condiments

FIGURE S10 Similarities between product category and year within notifications on mycotoxins.

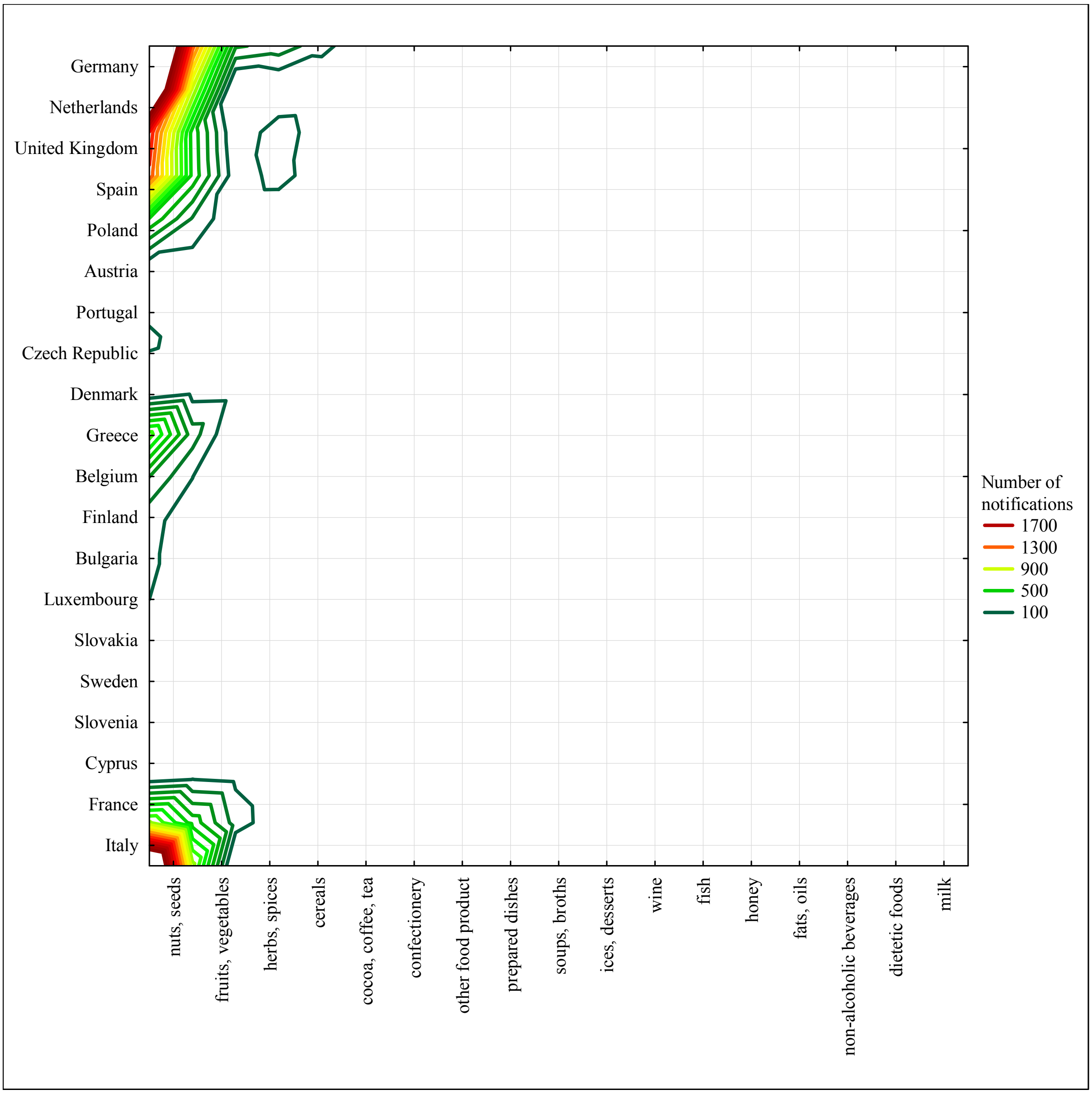

cereals – cereals and bakery products
cocoa, coffee, tea – cocoa and cocoa preparations, coffee and tea
dietetic foods – dietetic foods, food supplements, fortified foods
fats, oils – fats and oils
fish – fish and fish products
fruits, vegetables – fruits and vegetables
herbs, spices – herbs and spices
honey – honey and royal jelly
ices, desserts – ices and desserts
milk – milk and milk products
nuts, seeds – nuts, nut products and seeds
other food product – other food product / mixed
prepared dishes – prepared dishes and snacks
soups, broths – soups, broths, sauces and condiments

FIGURE S11 Similarities between product category and notifying country within notifications on mycotoxins.

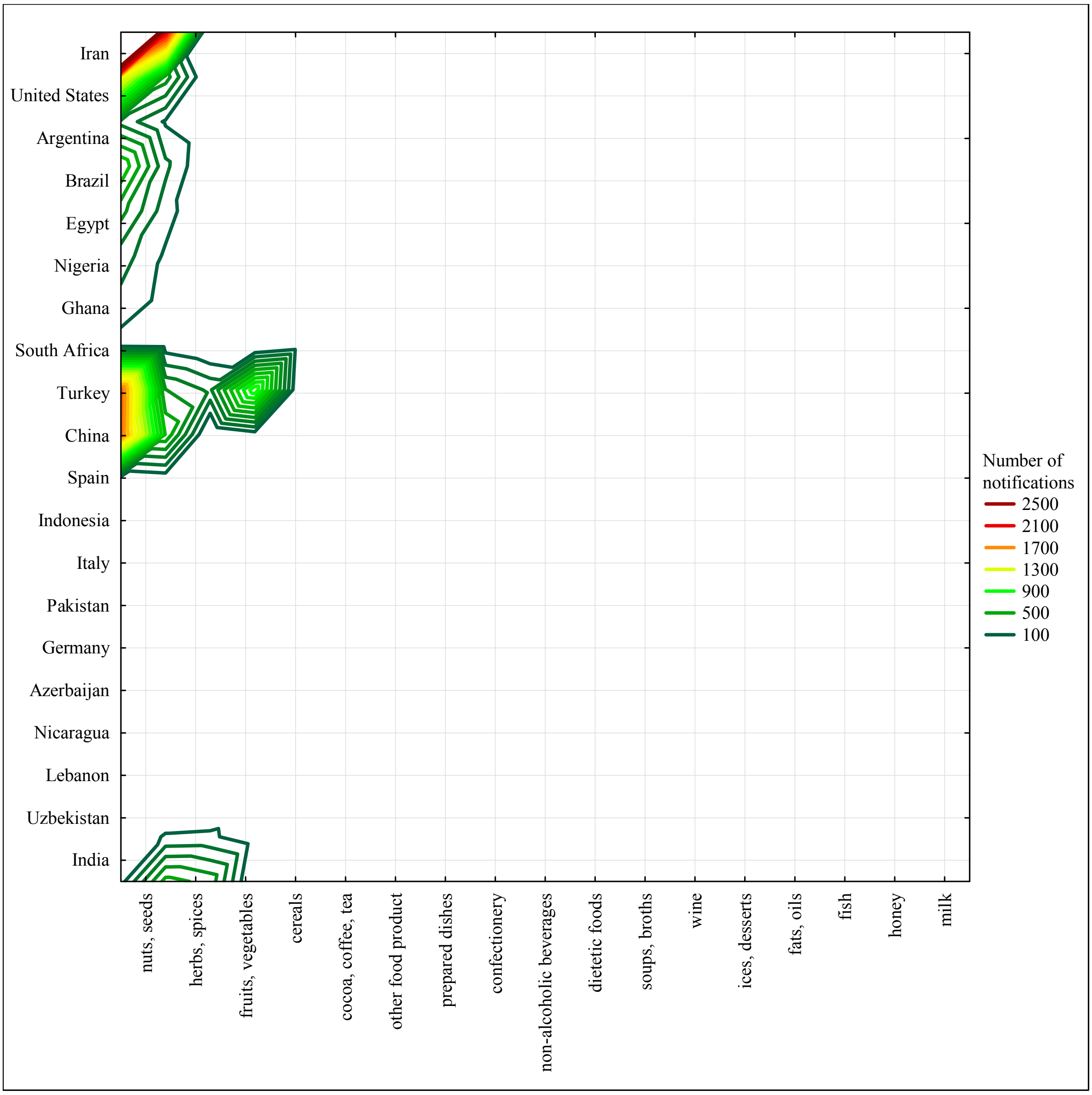

cereals – cereals and bakery products
cocoa, coffee, tea – cocoa and cocoa preparations, coffee and tea
dietetic foods – dietetic foods, food supplements, fortified foods
fats, oils – fats and oils
fish – fish and fish products
fruits, vegetables – fruits and vegetables
herbs, spices – herbs and spices
honey – honey and royal jelly
ices, desserts – ices and desserts
milk – milk and milk products
nuts, seeds – nuts, nut products and seeds
other food product – other food product / mixed
prepared dishes – prepared dishes and snacks
soups, broths – soups, broths, sauces and condiments

FIGURE S12 Similarities between product category and origin country within notifications on mycotoxins.

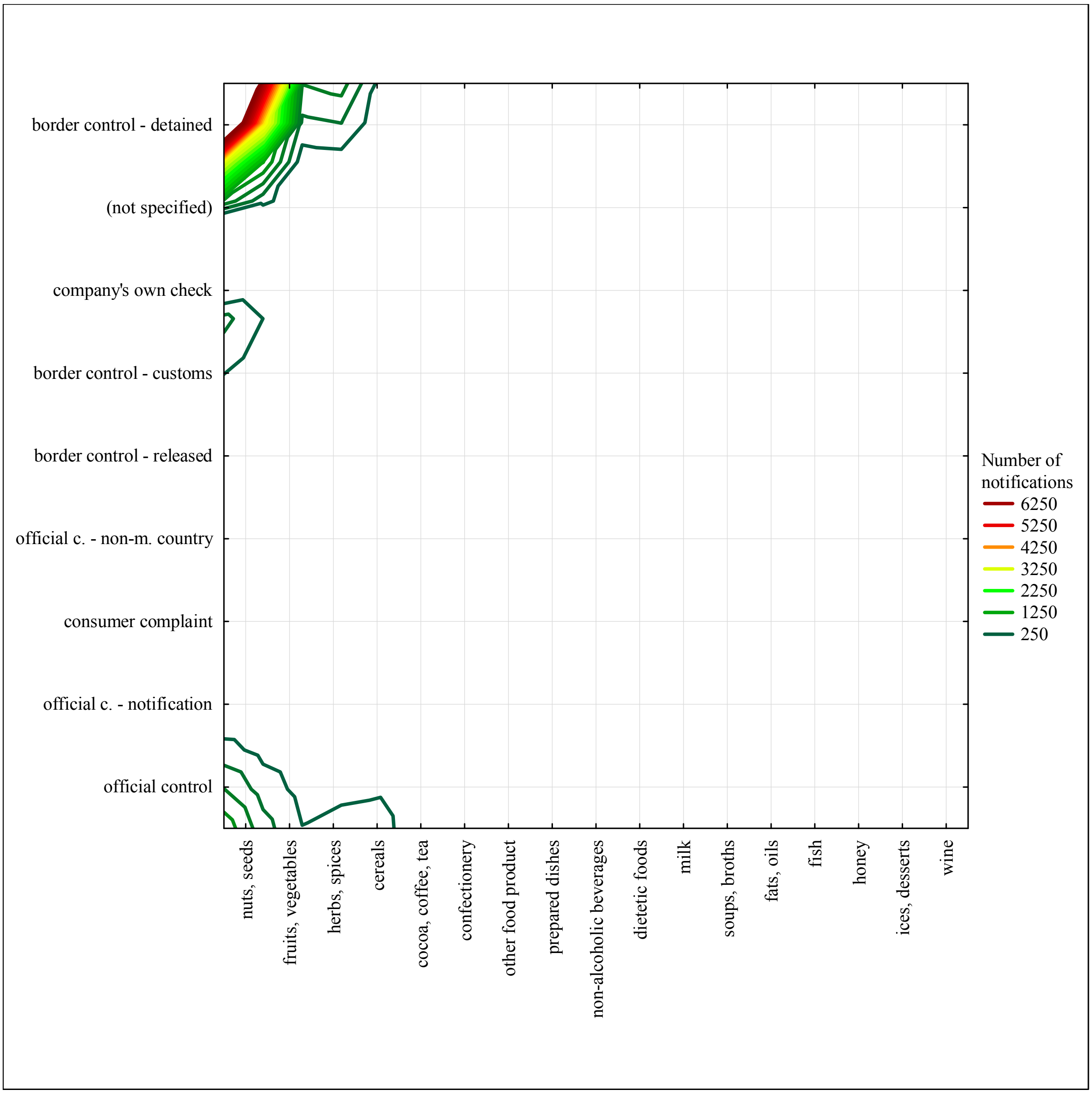

cereals – cereals and bakery products
cocoa, coffee, tea – cocoa and cocoa preparations, coffee and tea
dietetic foods – dietetic foods, food supplements, fortified foods
fats, oils – fats and oils
fish – fish and fish products
fruits, vegetables – fruits and vegetables
herbs, spices – herbs and spices
honey – honey and royal jelly
ices, desserts – ices and desserts
milk – milk and milk products
nuts, seeds – nuts, nut products and seeds
other food product – other food product / mixed
prepared dishes – prepared dishes and snacks
soups, broths – soups, broths, sauces and condiments

border control - customs – border control - consignment under customs
border control - detained – border control - consignment detained
border control - released – border control - consignment released
official c. - non-m. country – official control in non-member country
official c. - notification – official control following RASFF notification
official control – official control on the market

FIGURE S13 Similarities between product category and notification basis within notifications on mycotoxins.

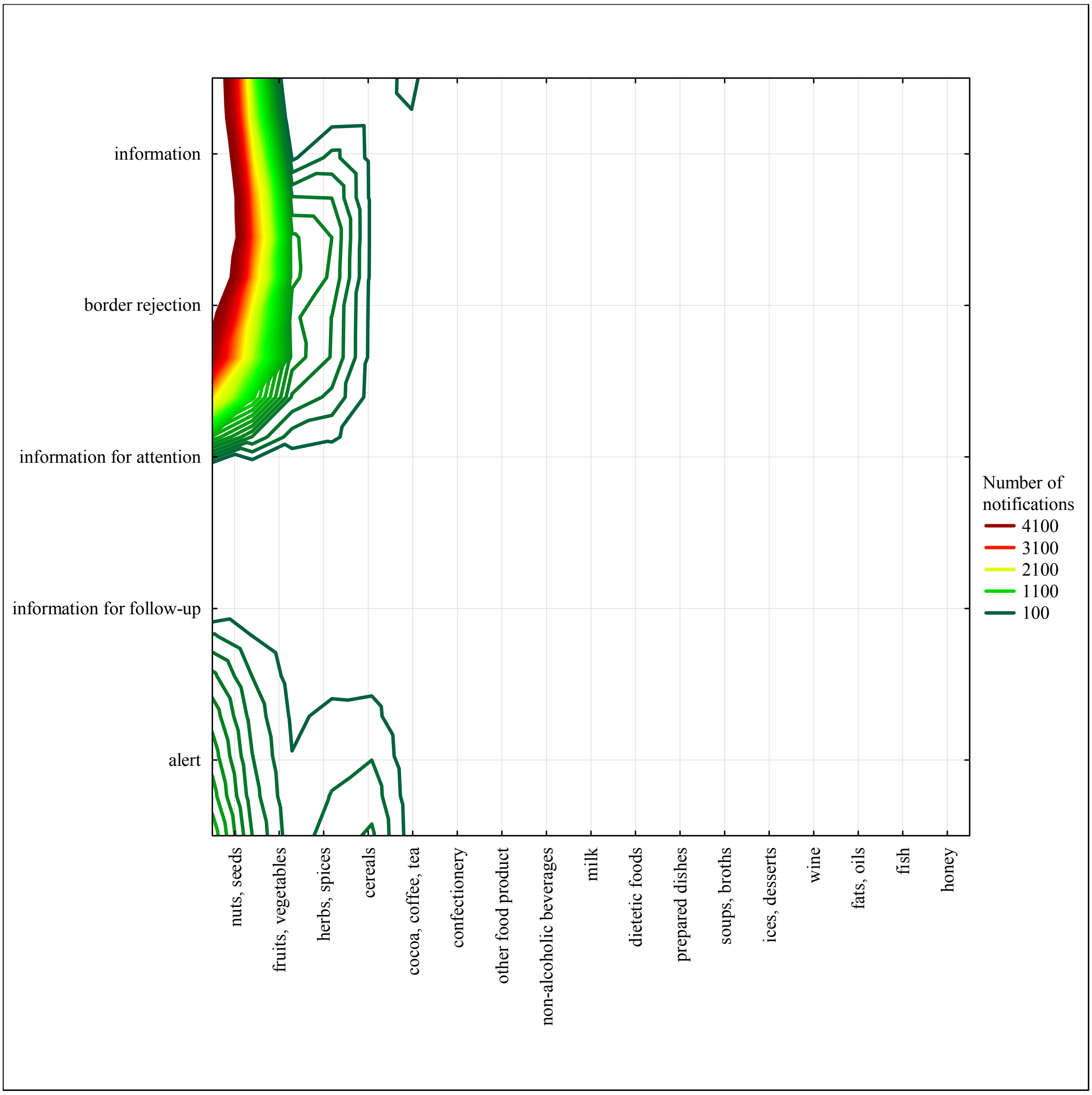

cereals – cereals and bakery products
cocoa, coffee, tea – cocoa and cocoa preparations, coffee and tea
dietetic foods – dietetic foods, food supplements, fortified foods
fats, oils – fats and oils
fish – fish and fish products
fruits, vegetables – fruits and vegetables
herbs, spices – herbs and spices
honey – honey and royal jelly
ices, desserts – ices and desserts
milk – milk and milk products
nuts, seeds – nuts, nut products and seeds
other food product – other food product / mixed
prepared dishes – prepared dishes and snacks
soups, broths – soups, broths, sauces and condiments

FIGURE S14 Similarities between product category and notification type within notifications on mycotoxins.

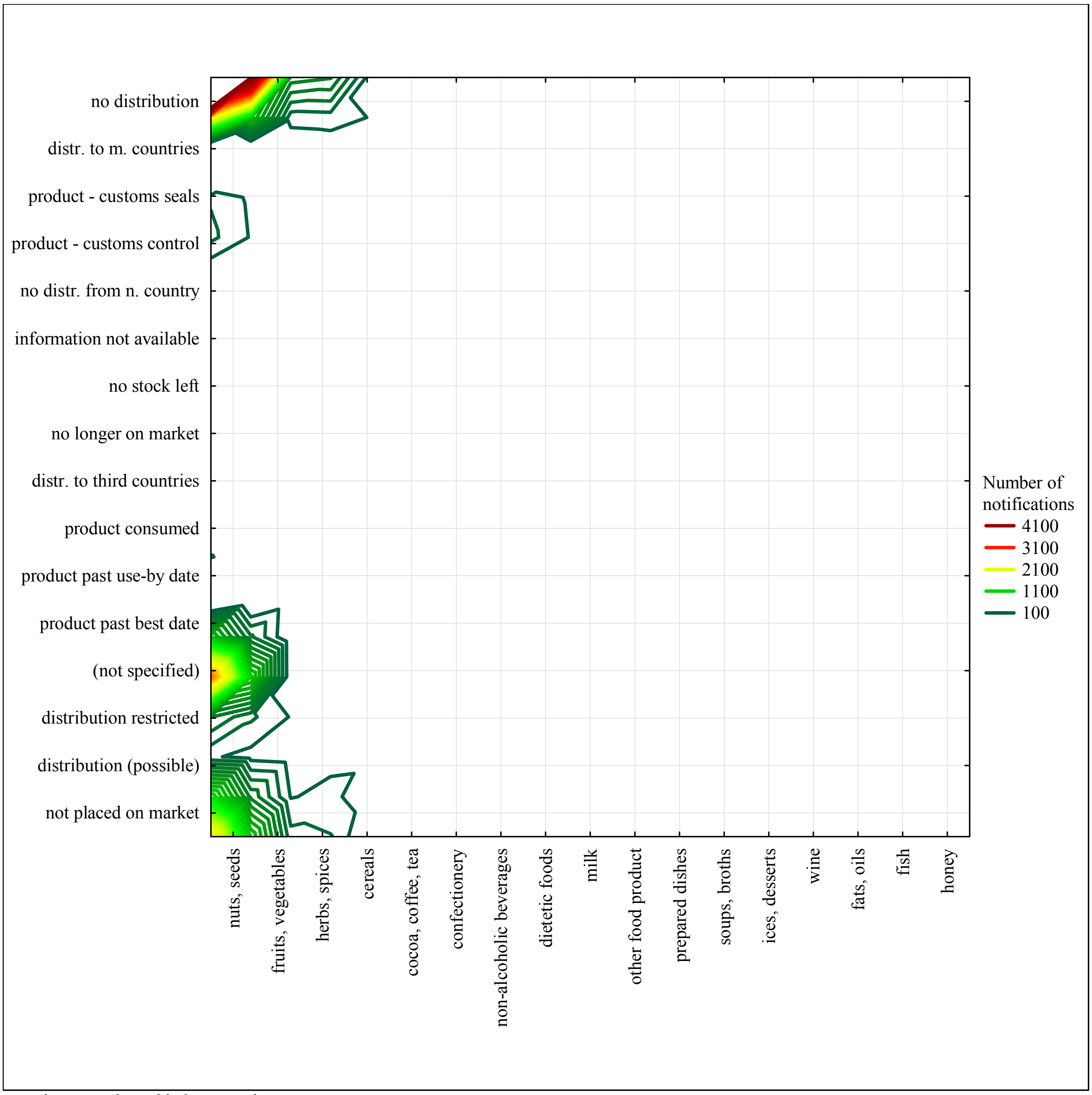

cereals – cereals and bakery products
cocoa, coffee, tea – cocoa and cocoa preparations, coffee and tea
dietetic foods – dietetic foods, food supplements, fortified foods
fats, oils – fats and oils
fish – fish and fish products
fruits, vegetables – fruits and vegetables
herbs, spices – herbs and spices
honey – honey and royal jelly
ices, desserts – ices and desserts
milk – milk and milk products
nuts, seeds – nuts, nut products and seeds
other food product – other food product / mixed
prepared dishes – prepared dishes and snacks
soups, broths – soups, broths, sauces and condiments

distr. to m. countries – distribution to other member countries
distr. to third countries – distribution to third countries
distribution (possible) – distribution on the market (possible)
distribution restricted – distribution restricted to notifying country
information not available – information on distribution not (yet) available
no distr. from n. country – no distribution from notifying country
no longer on market – product (presumably) no longer on the market
not placed on market – product not (yet) placed on the market
product - customs control – product under customs control
product - customs seals – product allowed to travel to destination under customs seals
product consumed – product already consumed
product past best date – product past best before date

FIGURE S15 Similarities between product category and distribution status within notifications on mycotoxins.

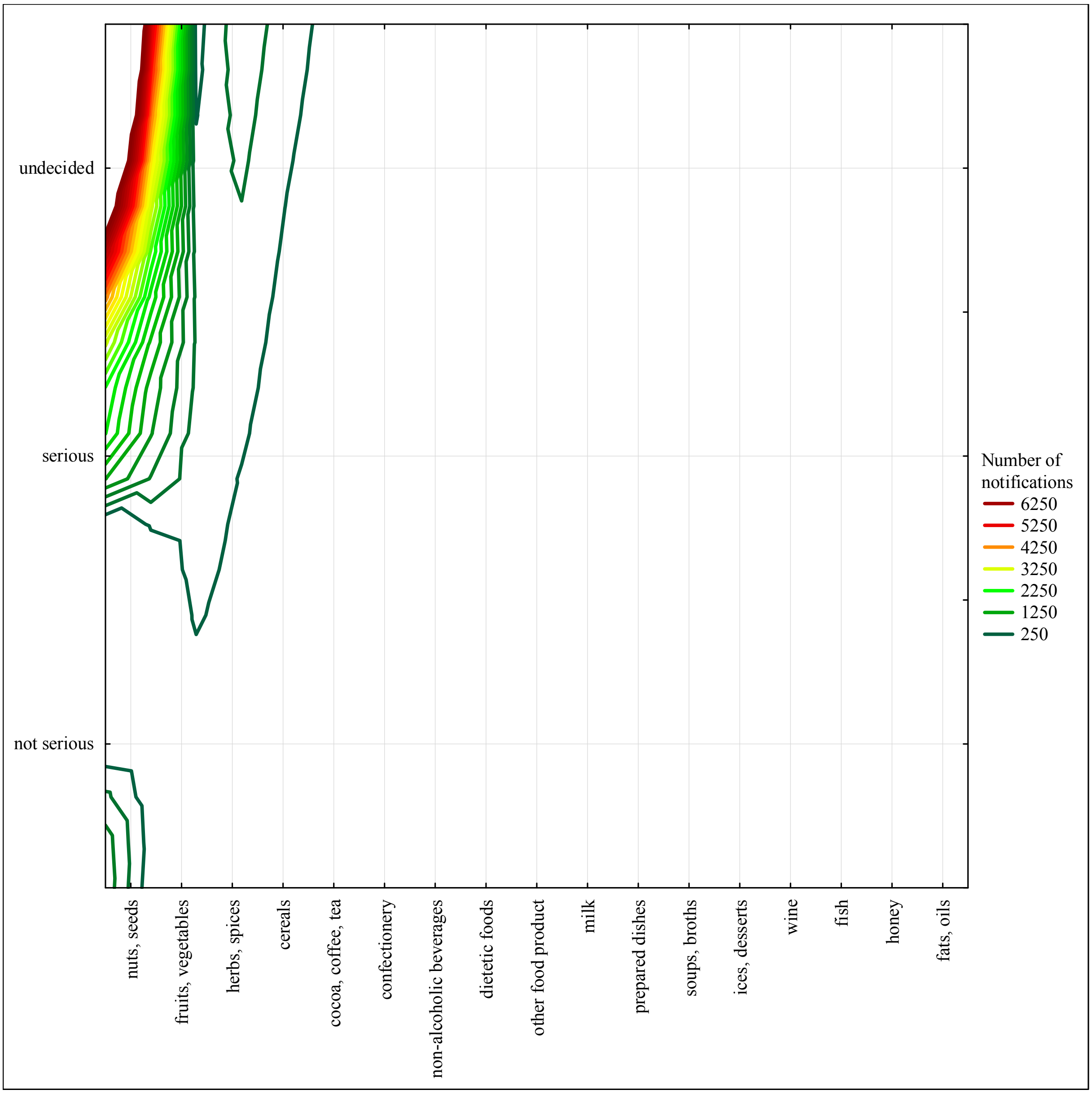

cereals – cereals and bakery products
cocoa, coffee, tea – cocoa and cocoa preparations, coffee and tea
dietetic foods – dietetic foods, food supplements, fortified foods
fats, oils – fats and oils
fish – fish and fish products
fruits, vegetables – fruits and vegetables
herbs, spices – herbs and spices
honey – honey and royal jelly
ices, desserts – ices and desserts
milk – milk and milk products
nuts, seeds – nuts, nut products and seeds
other food product – other food product / mixed
prepared dishes – prepared dishes and snacks
soups, broths – soups, broths, sauces and condiments

FIGURE S16 Similarities between product category and risk decision within notifications on mycotoxins.

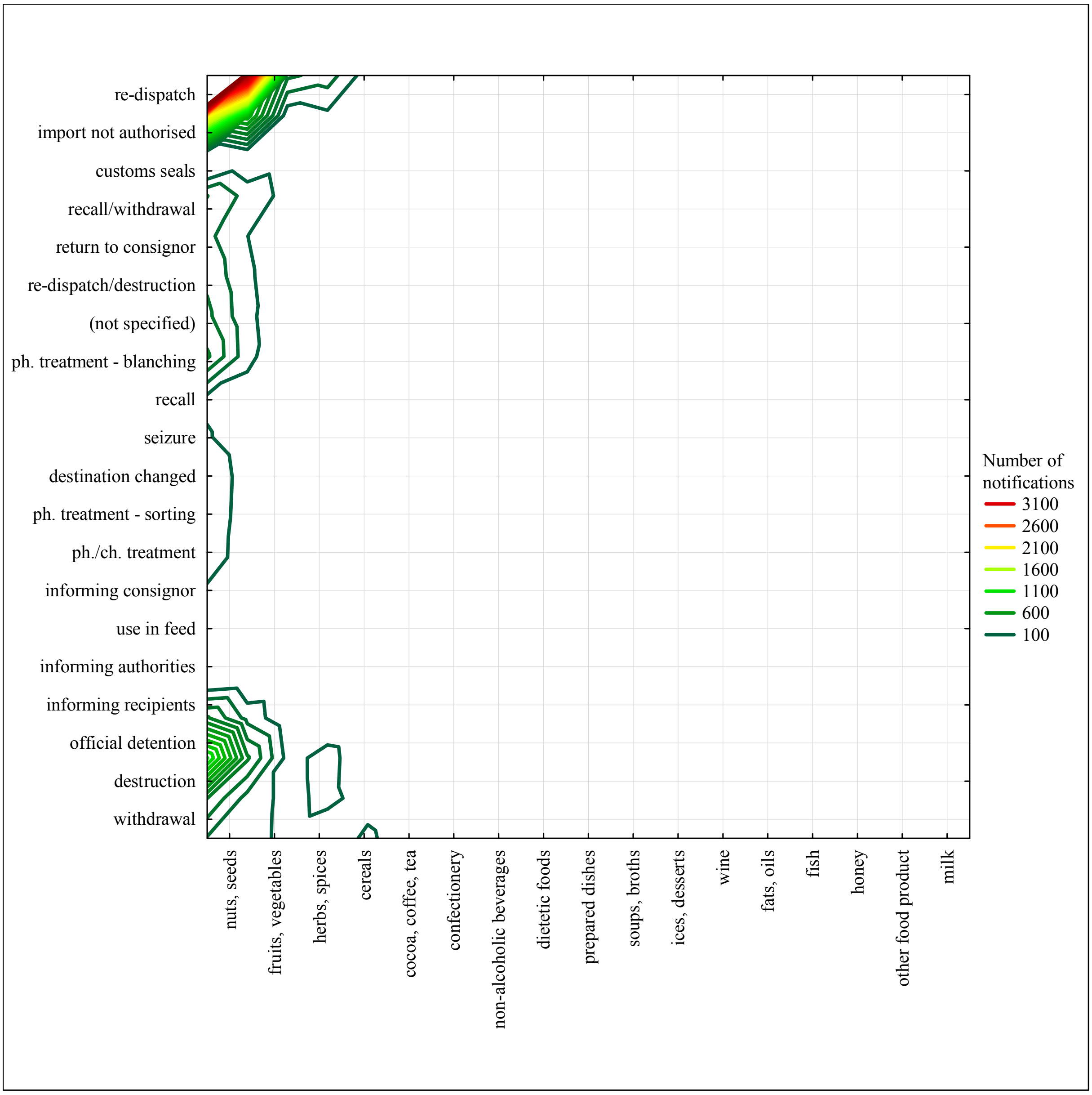

cereals – cereals and bakery products
cocoa, coffee, tea – cocoa and cocoa preparations, coffee and tea
dietetic foods – dietetic foods, food supplements, fortified foods
fats, oils – fats and oils
fish – fish and fish products
fruits, vegetables – fruits and vegetables
herbs, spices – herbs and spices
honey – honey and royal jelly
ices, desserts – ices and desserts
milk – milk and milk products
nuts, seeds – nuts, nut products and seeds
other food product – other food product / mixed
prepared dishes – prepared dishes and snacks
soups, broths – soups, broths, sauces and condiments

customs seals – placed under customs seals
destination changed – destination of the product changed
ph. treatment - blanching – physical treatment - blanching
ph. treatment - sorting – physical treatment - sorting
ph./ch. treatment – physical/chemical treatment
recall – recall from consumers
recall/withdrawal – product recall or withdrawal
re-dispatch/destruction – re-dispatch or destruction
withdrawal – withdrawal from the market

FIGURE S17 Similarities between product category and action taken within notifications on mycotoxins.

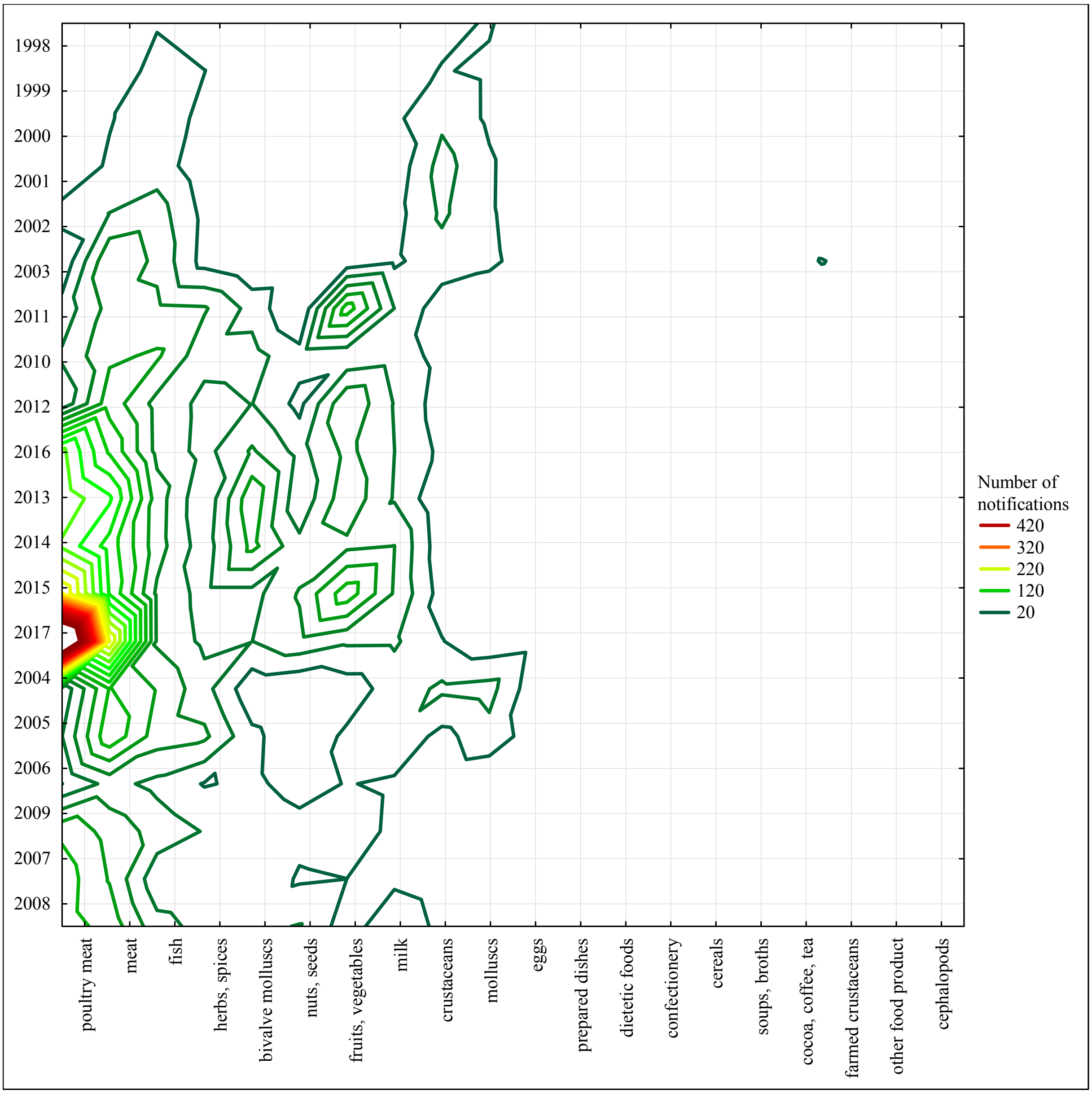

bivalve molluscs – bivalve molluscs and products thereof
cephalopods – cephalopods and products thereof
cereals – cereals and bakery products
cocoa, coffee, tea – cocoa and cocoa preparations, coffee and tea
crustaceans – crustaceans and products thereof
dietetic foods – dietetic foods, food supplements, fortified foods
eggs – eggs and egg products
farmed crustaceans – farmed crustaceans and products thereof - (obsolete)
fish – fish and fish products
fruits, vegetables – fruits and vegetables
herbs, spices – herbs and spices
meat – meat and meat products (other than poultry)
milk – milk and milk products
molluscs – molluscs and products thereof - (obsolete)
nuts, seedsnuts, nut products and seeds
other food product – other food product / mixed
poultry meat – poultry meat and poultry meat products
prepared dishes – prepared dishes and snacks
soups, broths – soups, broths, sauces and condiments

FIGURE S18 Similarities between product category and year within notifications on pathogenic micro-organisms.

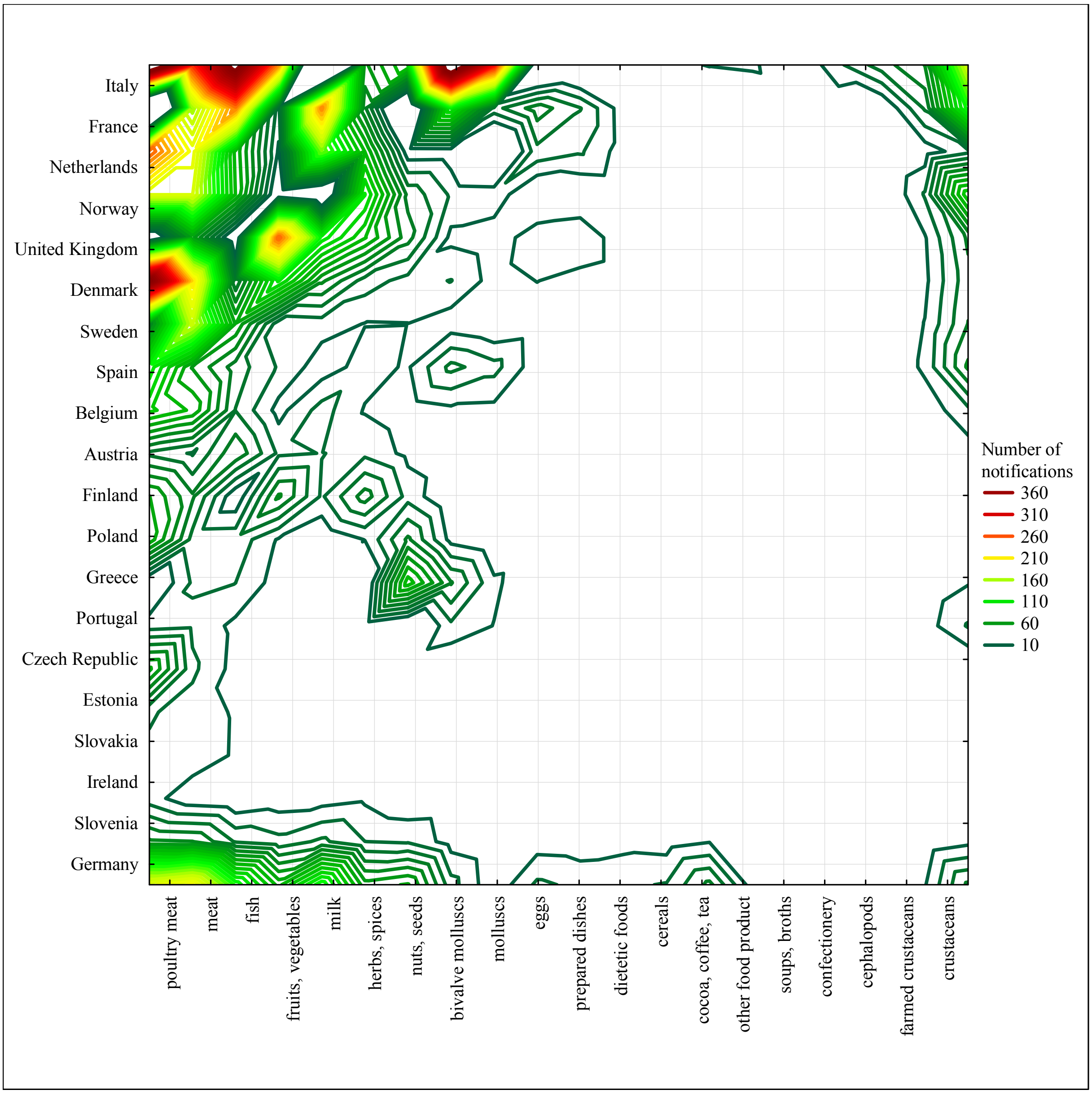

bivalve molluscs – bivalve molluscs and products thereof
cephalopods – cephalopods and products thereof
cereals – cereals and bakery products
cocoa, coffee, tea – cocoa and cocoa preparations, coffee and tea
crustaceans – crustaceans and products thereof
dietetic foods – dietetic foods, food supplements, fortified foods
eggs – eggs and egg products
farmed crustaceans – farmed crustaceans and products thereof - (obsolete)
fish – fish and fish products
fruits, vegetables – fruits and vegetables
herbs, spices – herbs and spices
meat – meat and meat products (other than poultry)
milk – milk and milk products
molluscs – molluscs and products thereof - (obsolete)
nuts, seedsnuts, nut products and seeds
other food product – other food product / mixed
poultry meat – poultry meat and poultry meat products
prepared dishes – prepared dishes and snacks
soups, broths – soups, broths, sauces and condiments

FIGURE S19 Similarities between product category and notifying country within notifications on pathogenic micro-organisms.

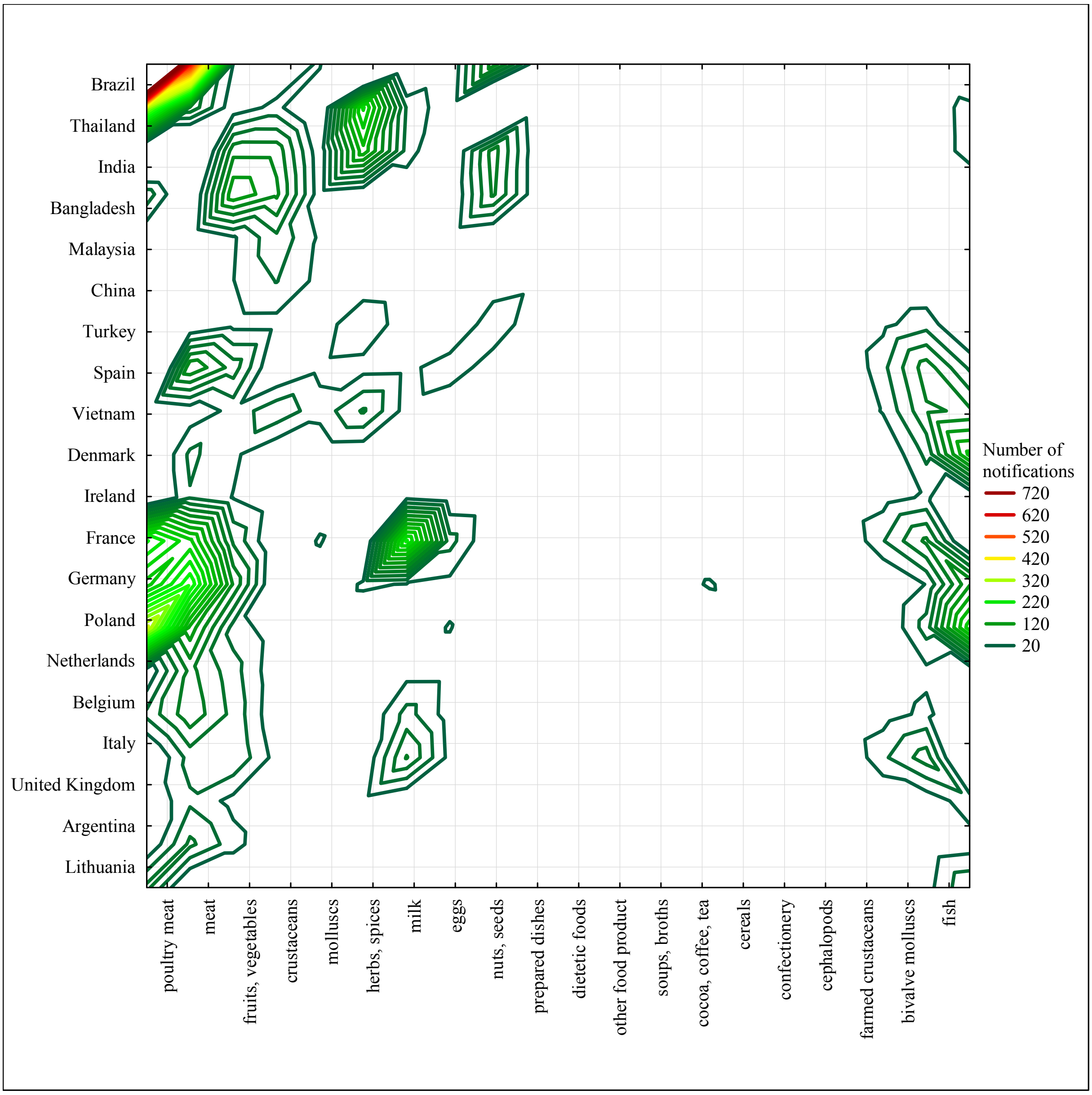

bivalve molluscs – bivalve molluscs and products thereof
cephalopods – cephalopods and products thereof
cereals – cereals and bakery products
cocoa, coffee, tea – cocoa and cocoa preparations, coffee and tea
crustaceans – crustaceans and products thereof
dietetic foods – dietetic foods, food supplements, fortified foods
eggs – eggs and egg products
farmed crustaceans – farmed crustaceans and products thereof - (obsolete)
fish – fish and fish products
fruits, vegetables – fruits and vegetables
herbs, spices – herbs and spices
meat – meat and meat products (other than poultry)
milk – milk and milk products
molluscs – molluscs and products thereof - (obsolete)
nuts, seedsnuts, nut products and seeds
other food product – other food product / mixed
poultry meat – poultry meat and poultry meat products
prepared dishes – prepared dishes and snacks
soups, broths – soups, broths, sauces and condiments

FIGURE S20 Similarities between product category and origin country within notifications on pathogenic micro-organisms.

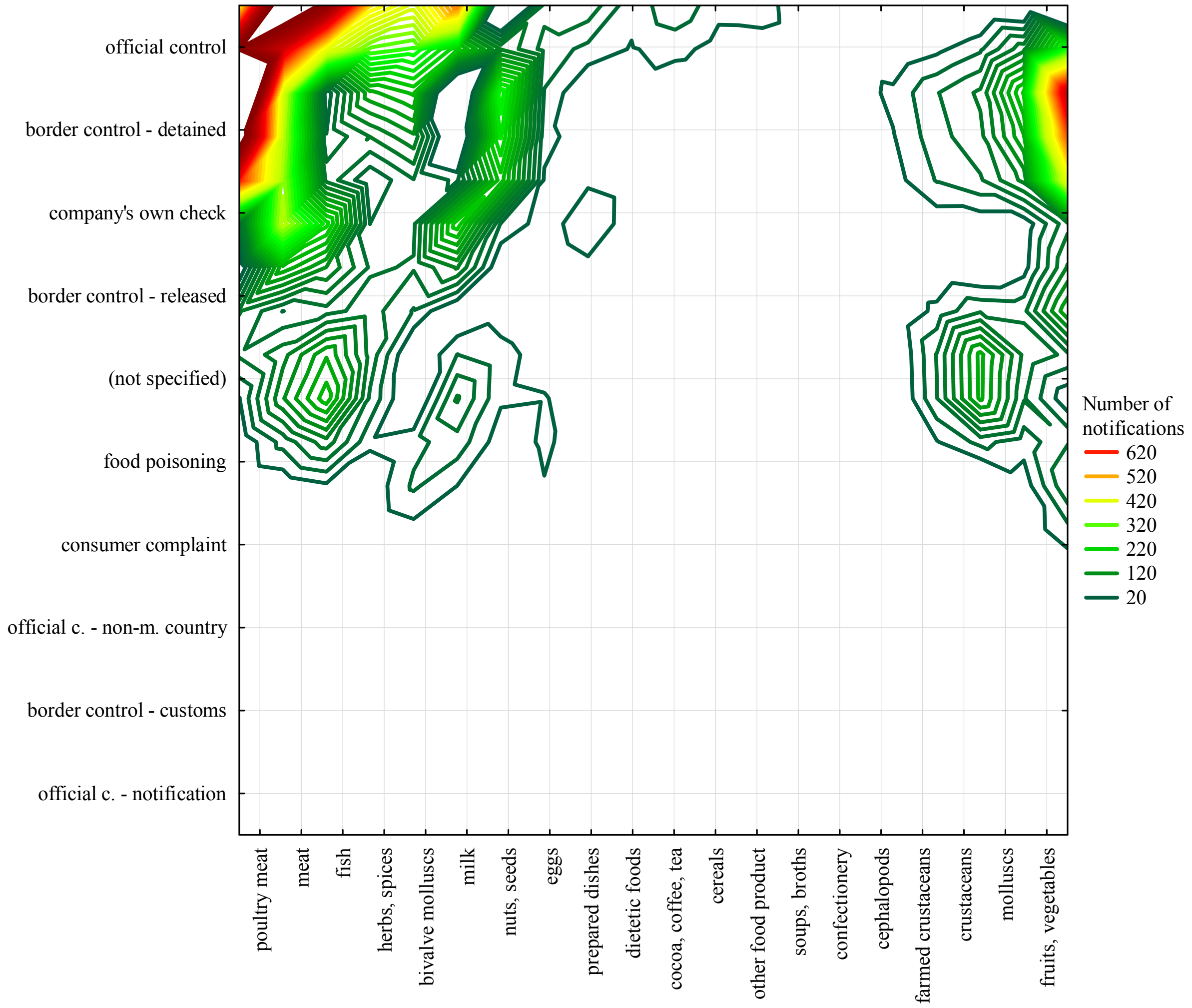

bivalve molluscs – bivalve molluscs and products thereof
cephalopods – cephalopods and products thereof
cereals – cereals and bakery products
cocoa, coffee, tea – cocoa and cocoa preparations, coffee and tea
crustaceans – crustaceans and products thereof
dietetic foods – dietetic foods, food supplements, fortified foods
eggs – eggs and egg products
farmed crustaceans – farmed crustaceans and products thereof - (obsolete)
fish – fish and fish products
fruits, vegetables – fruits and vegetables
herbs, spices – herbs and spices
meat – meat and meat products (other than poultry)
milk – milk and milk products
molluscs – molluscs and products thereof - (obsolete)
nuts, seedsnuts, nut products and seeds
other food product – other food product / mixed
poultry meat – poultry meat and poultry meat products
prepared dishes – prepared dishes and snacks
soups, broths – soups, broths, sauces and condiments

border control - customs – border control - consignment under customs
border control - detained – border control - consignment detained
border control - released – border control - consignment released
official c. - non-m. country – official control in non-member country
official c. - notification – official control following RASFF notification
official control – official control on the market

FIGURE S21 Similarities between product category and notification basis within notifications on pathogenic micro-organisms.

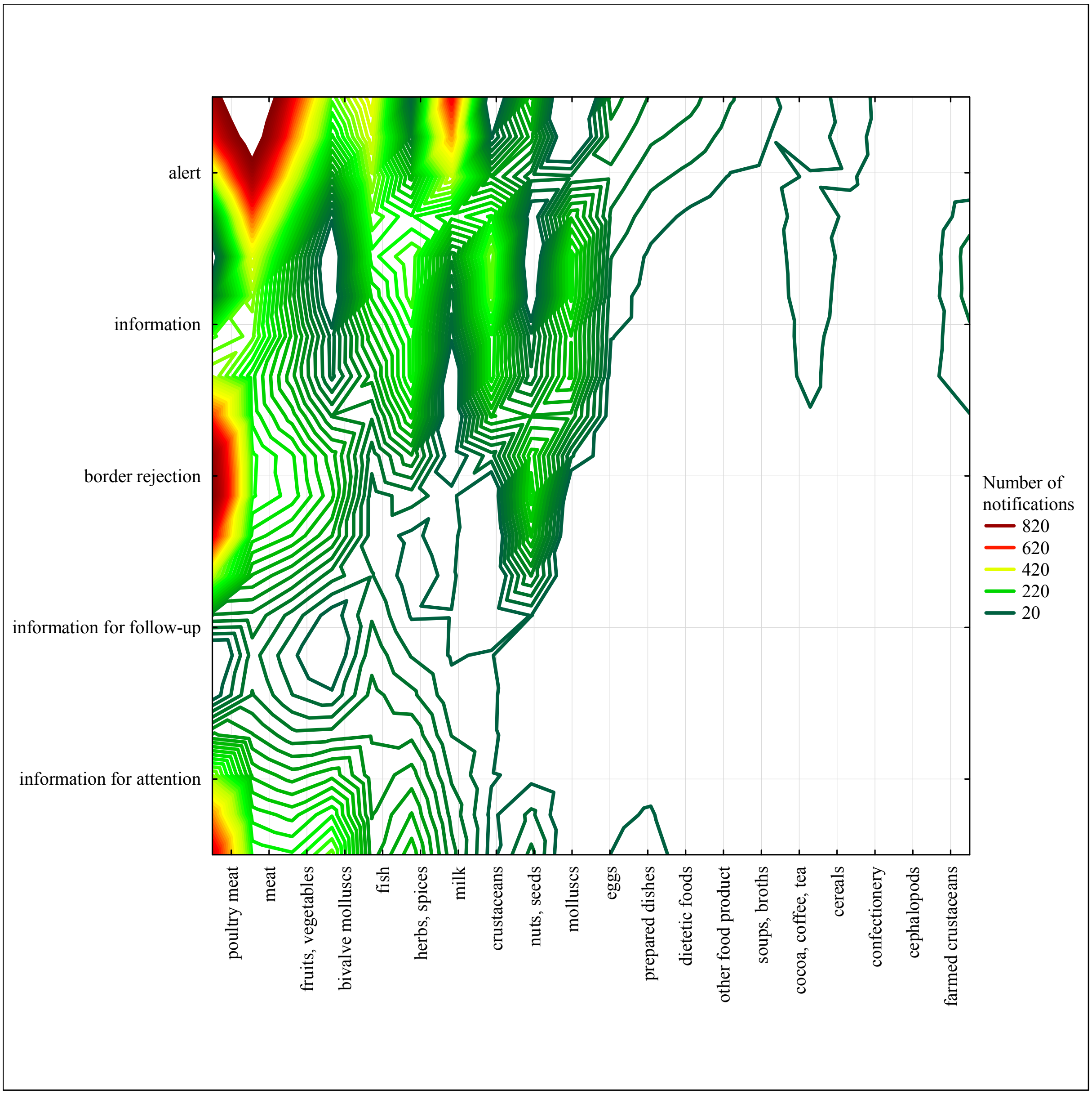

bivalve molluscs – bivalve molluscs and products thereof
cephalopods – cephalopods and products thereof
cereals – cereals and bakery products
cocoa, coffee, tea – cocoa and cocoa preparations, coffee and tea
crustaceans – crustaceans and products thereof
dietetic foods – dietetic foods, food supplements, fortified foods
eggs – eggs and egg products
farmed crustaceans – farmed crustaceans and products thereof - (obsolete)
fish – fish and fish products
fruits, vegetables – fruits and vegetables
herbs, spices – herbs and spices
meat – meat and meat products (other than poultry)
milk – milk and milk products
molluscs – molluscs and products thereof - (obsolete)
nuts, seedsnuts, nut products and seeds
other food product – other food product / mixed
poultry meat – poultry meat and poultry meat products
prepared dishes – prepared dishes and snacks
soups, broths – soups, broths, sauces and condiments

FIGURE S22 Similarities between product category and notification type within notifications on pathogenic micro-organisms.

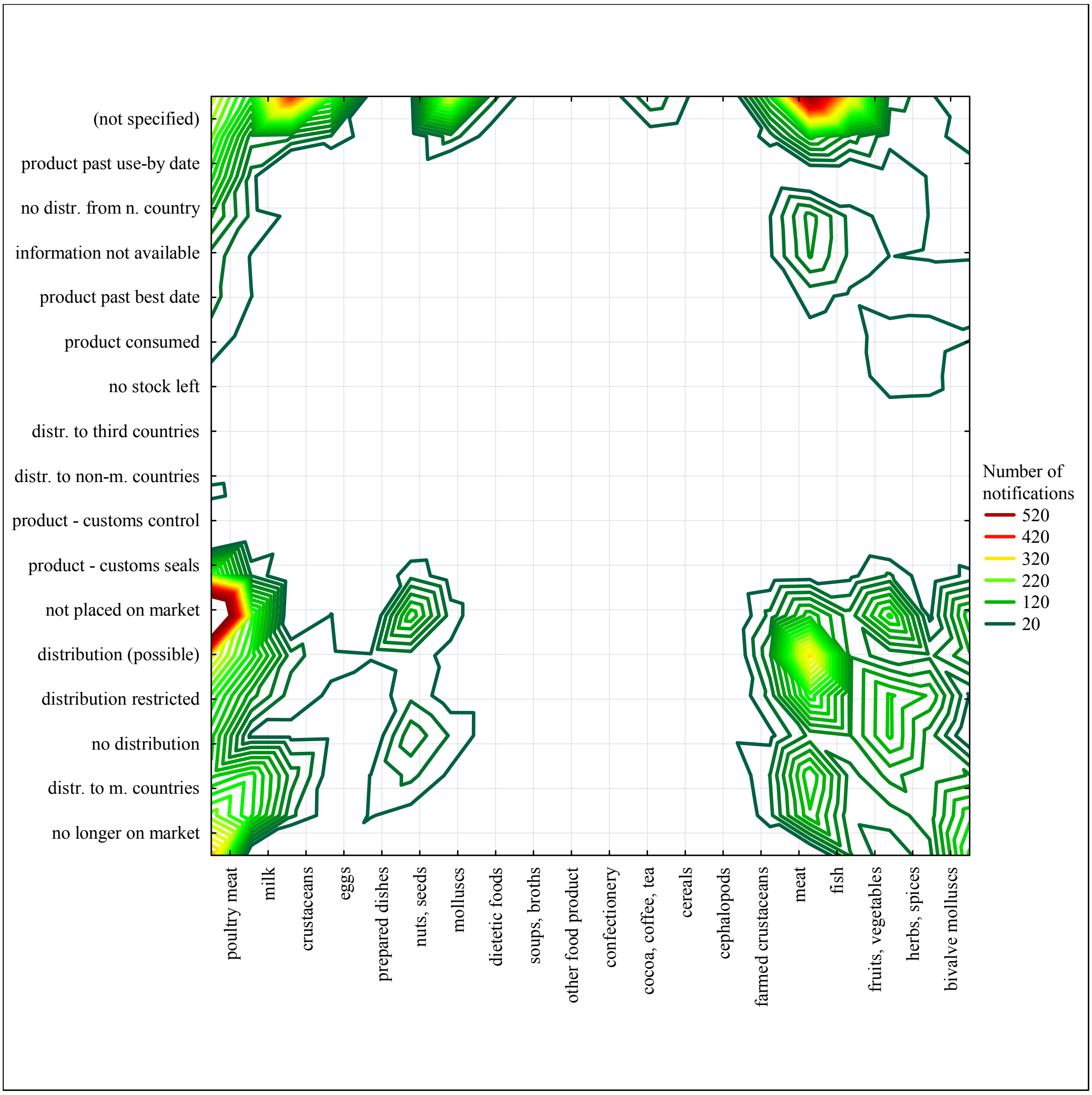

bivalve molluscs – bivalve molluscs and products thereof
cephalopods – cephalopods and products thereof
cereals – cereals and bakery products
cocoa, coffee, tea – cocoa and cocoa preparations, coffee and tea
crustaceans – crustaceans and products thereof
dietetic foods – dietetic foods, food supplements, fortified foods
eggs – eggs and egg products
farmed crustaceans – farmed crustaceans and products thereof - (obsolete)
fish – fish and fish products
fruits, vegetables – fruits and vegetables
herbs, spices – herbs and spices
meat – meat and meat products (other than poultry)
milk – milk and milk products
molluscs – molluscs and products thereof - (obsolete)
nuts, seedsnuts, nut products and seeds
other food product – other food product / mixed
poultry meat – poultry meat and poultry meat products
prepared dishes – prepared dishes and snacks
soups, broths – soups, broths, sauces and condiments

distr. to m. countries – distribution to other member countries
distr. to non-m. countries – distribution to non-member countries
distr. to third countries – distribution to third countries
distribution (possible) – distribution on the market (possible)
distribution restricted – distribution restricted to notifying country
information not available – information on distribution not (yet) available
no distr. from n. country – no distribution from notifying country
no longer on market – product (presumably) no longer on the market
not placed on market – product not (yet) placed on the market
product - customs seals – product allowed to travel to destination under customs seals
product consumed – product already consumed
product - customs control – product under customs control
product past best date – product past best before date

FIGURE S23 Similarities between product category and distribution status within notifications on pathogenic micro-organisms.

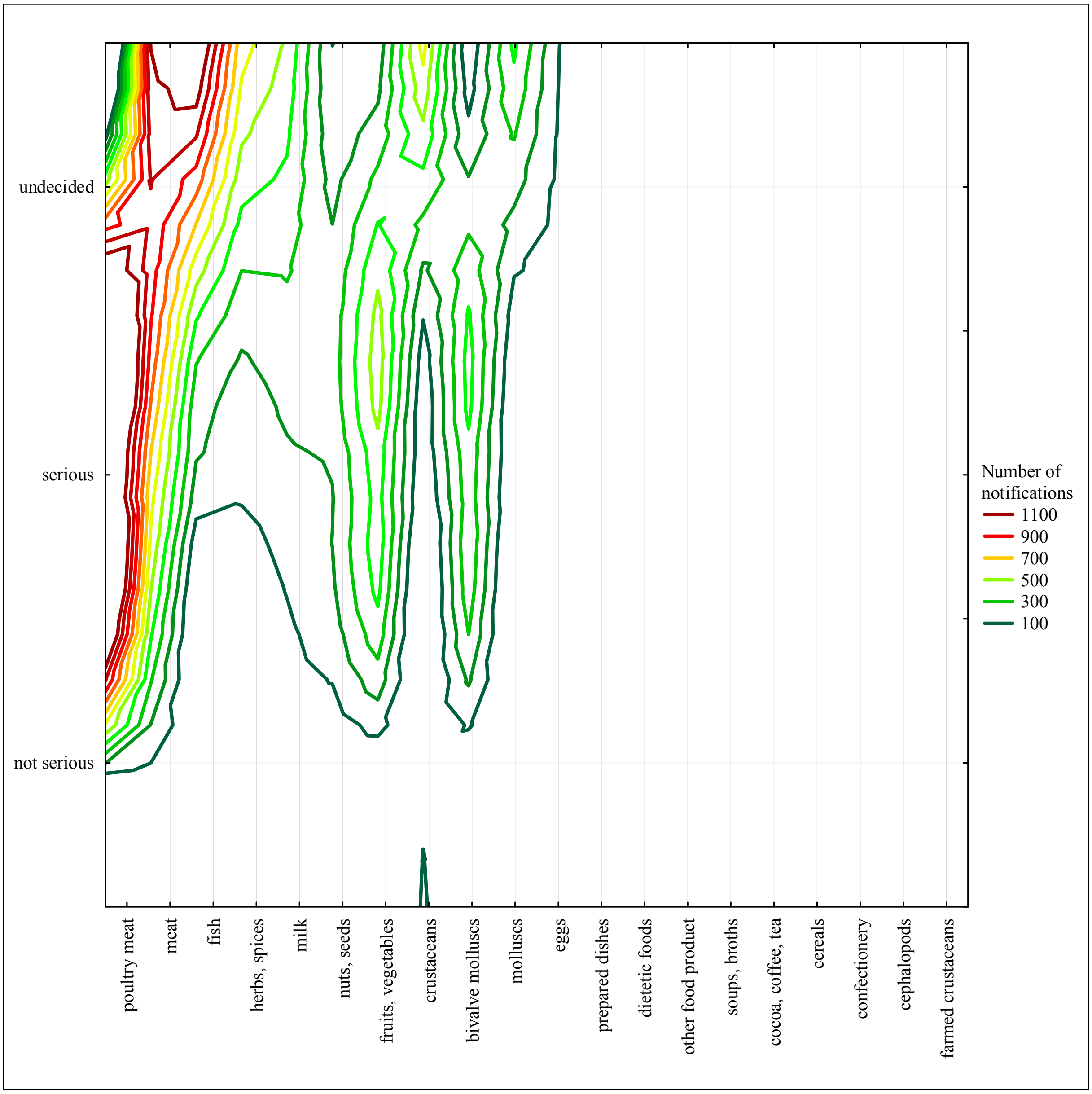

bivalve molluscs – bivalve molluscs and products thereof
cephalopods – cephalopods and products thereof
cereals – cereals and bakery products
cocoa, coffee, tea – cocoa and cocoa preparations, coffee and tea
crustaceans – crustaceans and products thereof
dietetic foods – dietetic foods, food supplements, fortified foods
eggs – eggs and egg products
farmed crustaceans – farmed crustaceans and products thereof - (obsolete)
fish – fish and fish products
fruits, vegetables – fruits and vegetables
herbs, spices – herbs and spices
meat – meat and meat products (other than poultry)
milk – milk and milk products
molluscs – molluscs and products thereof - (obsolete)
nuts, seedsnuts, nut products and seeds
other food product – other food product / mixed
poultry meat – poultry meat and poultry meat products
prepared dishes – prepared dishes and snacks
soups, broths – soups, broths, sauces and condiments

FIGURE S24 Similarities between product category and risk decision within notifications on pathogenic micro-organisms.

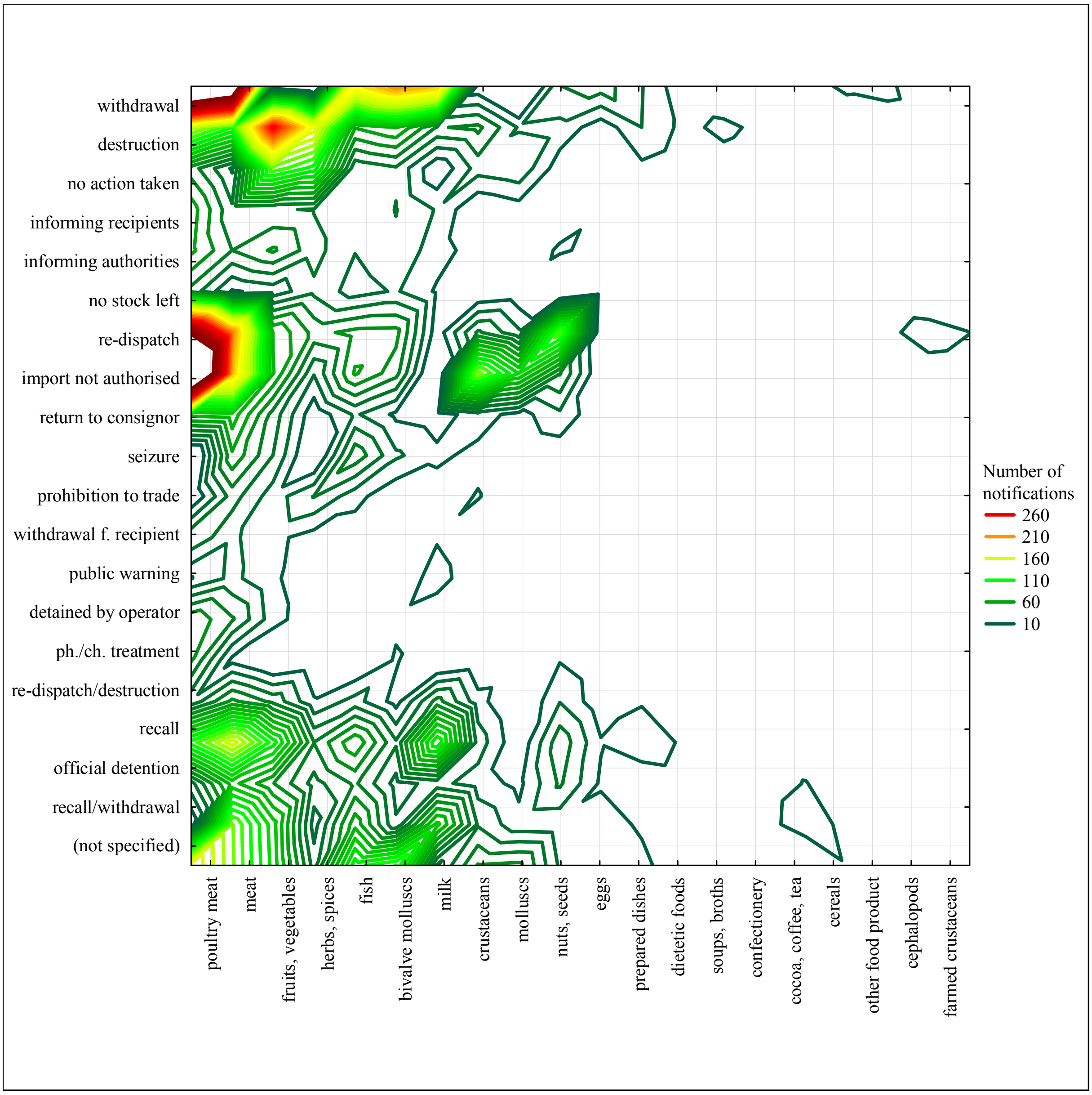

bivalve molluscs – bivalve molluscs and products thereof
cephalopods – cephalopods and products thereof
cereals – cereals and bakery products
cocoa, coffee, tea – cocoa and cocoa preparations, coffee and tea
crustaceans – crustaceans and products thereof
dietetic foods – dietetic foods, food supplements, fortified foods
eggs – eggs and egg products
farmed crustaceans – farmed crustaceans and products thereof - (obsolete)
fish – fish and fish products
fruits, vegetables – fruits and vegetables
herbs, spices – herbs and spices
meat – meat and meat products (other than poultry)
milk – milk and milk products
molluscs – molluscs and products thereof - (obsolete)

nuts, seedsnuts, nut products and seeds
other food product – other food product / mixed
poultry meat – poultry meat and poultry meat products
prepared dishes – prepared dishes and snacks
soups, broths – soups, broths, sauces and condiments

ph./ch. treatment – physical/chemical treatment
prohibition to trade – prohibition to trade - sales ban
public warning – public warning - press release
recall – recall from consumers
recall/withdrawal – product recall or withdrawal
re-dispatch/destruction – re-dispatch or destruction
withdrawal – withdrawal from the market
withdrawal f. recipient – withdrawal from recipient(s)

FIGURE S25 Similarities between product category and action taken within notifications on pathogenic micro-organisms.

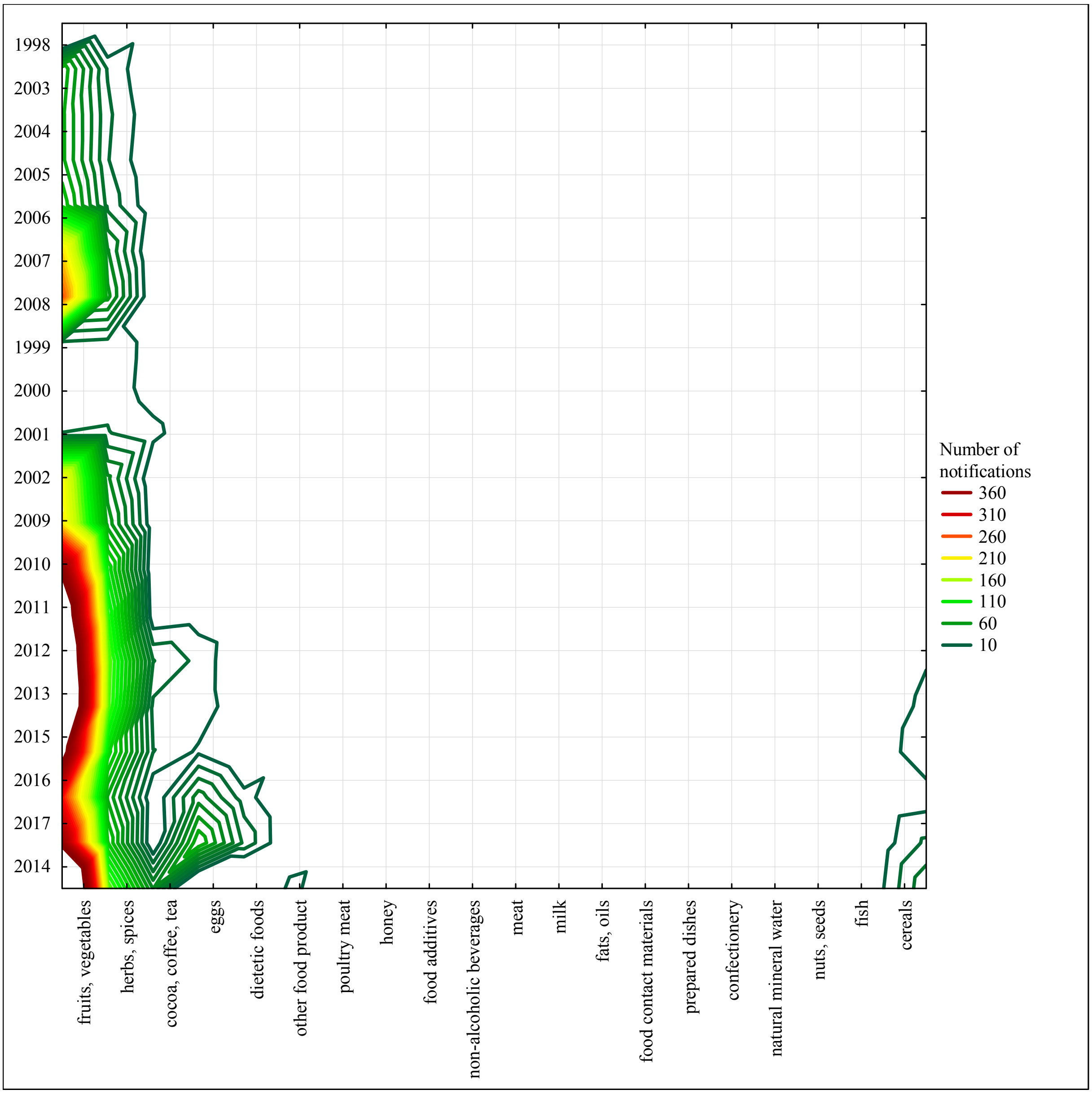

cereals – cereals and bakery products
cocoa, coffee, tea – cocoa and cocoa preparations, coffee and tea
dietetic foods – dietetic foods, food supplements, fortified foods
eggs – eggs and egg products
fats, oils – fats and oils
fish – fish and fish products
food additives – food additives and flavourings
fruits, vegetables – fruits and vegetables
herbs, spices – herbs and spices
honey – honey and royal jelly
meat – meat and meat products (other than poultry)
milk – milk and milk products
nuts, seeds – nuts, nut products and seeds
other food product – other food product / mixed
poultry meat – poultry meat and poultry meat products
prepared dishes – prepared dishes and snacks

FIGURE S26 Similarities between product category and year within notifications on pesticide residues.

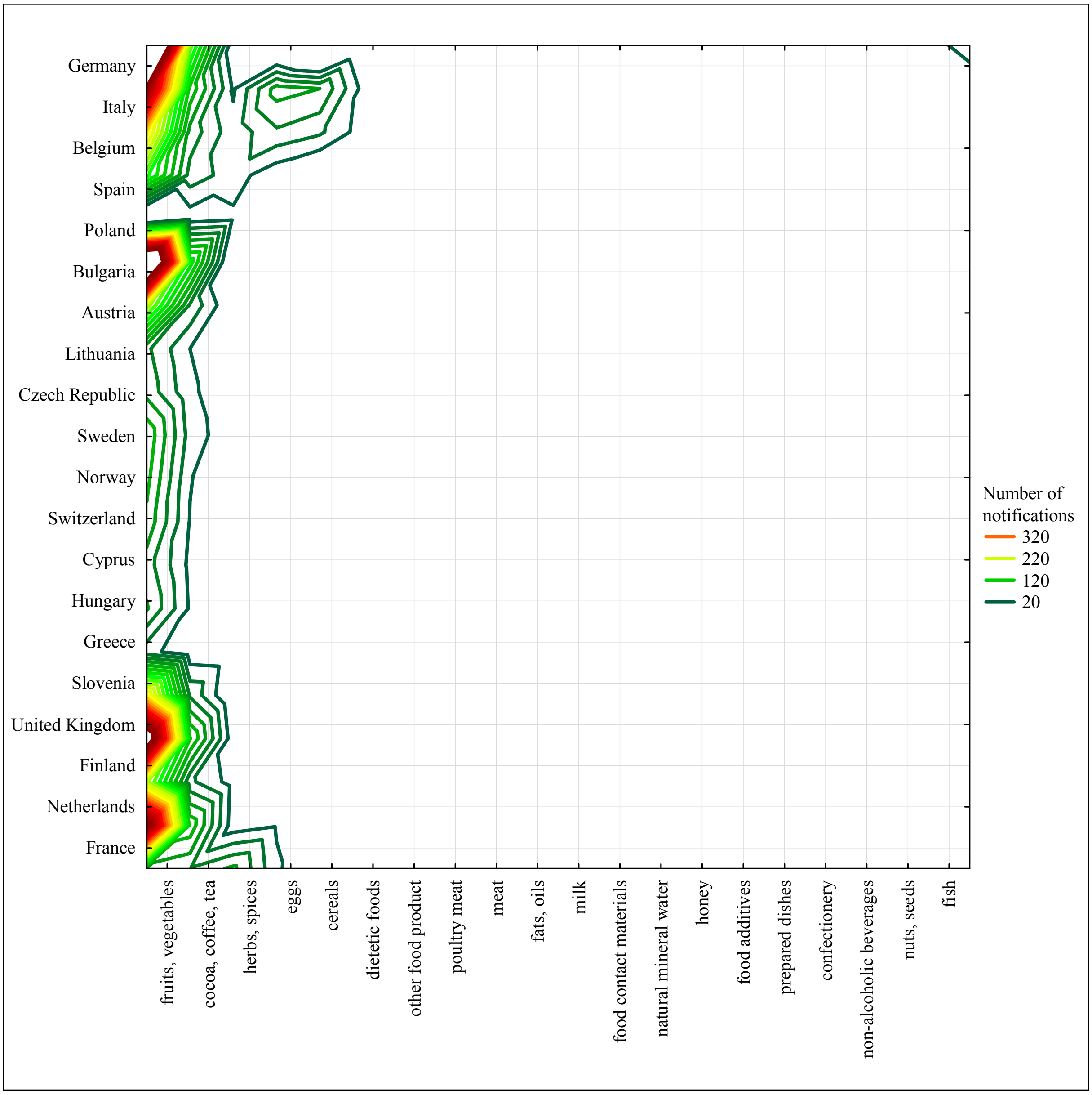

cereals – cereals and bakery products
cocoa, coffee, tea – cocoa and cocoa preparations, coffee and tea
dietetic foods – dietetic foods, food supplements, fortified foods
eggs – eggs and egg products
fats, oils – fats and oils
fish – fish and fish products
food additives – food additives and flavourings
fruits, vegetables – fruits and vegetables
herbs, spices – herbs and spices
honey – honey and royal jelly
meat – meat and meat products (other than poultry)
milk – milk and milk products
nuts, seeds – nuts, nut products and seeds
other food product – other food product / mixed
poultry meat – poultry meat and poultry meat products
prepared dishes – prepared dishes and snacks

FIGURE S27 Similarities between product category and notifying country within notifications on pesticide residues.

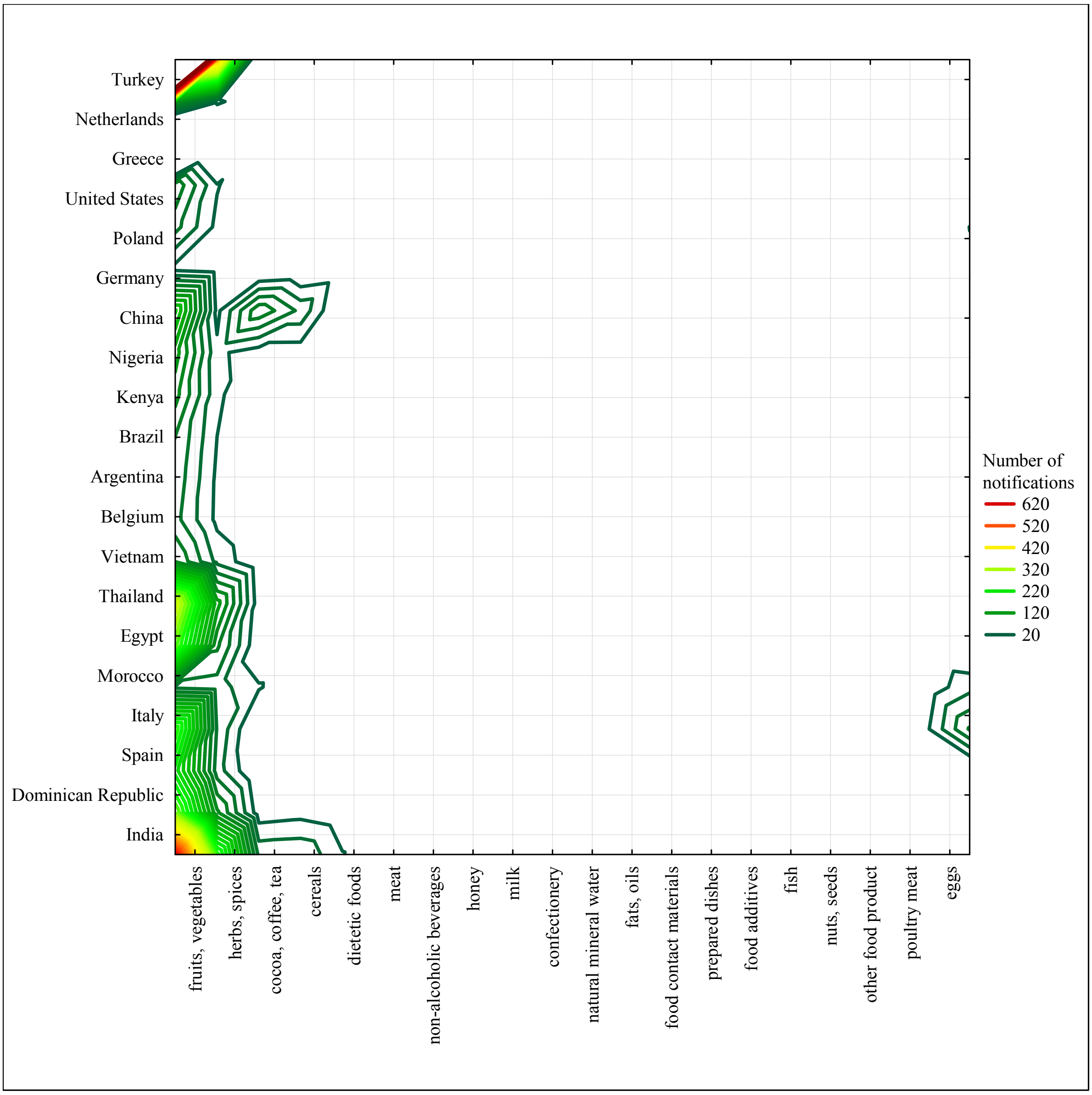

cereals – cereals and bakery products
cocoa, coffee, tea – cocoa and cocoa preparations, coffee and tea
dietetic foods – dietetic foods, food supplements, fortified foods
eggs – eggs and egg products
fats, oils – fats and oils
fish – fish and fish products
food additives – food additives and flavourings
fruits, vegetables – fruits and vegetables
herbs, spices – herbs and spices
honey – honey and royal jelly
meat – meat and meat products (other than poultry)
milk – milk and milk products
nuts, seeds – nuts, nut products and seeds
other food product – other food product / mixed
poultry meat – poultry meat and poultry meat products
prepared dishes – prepared dishes and snacks

FIGURE S28 Similarities between product category and origin country within notifications on pesticide residues.

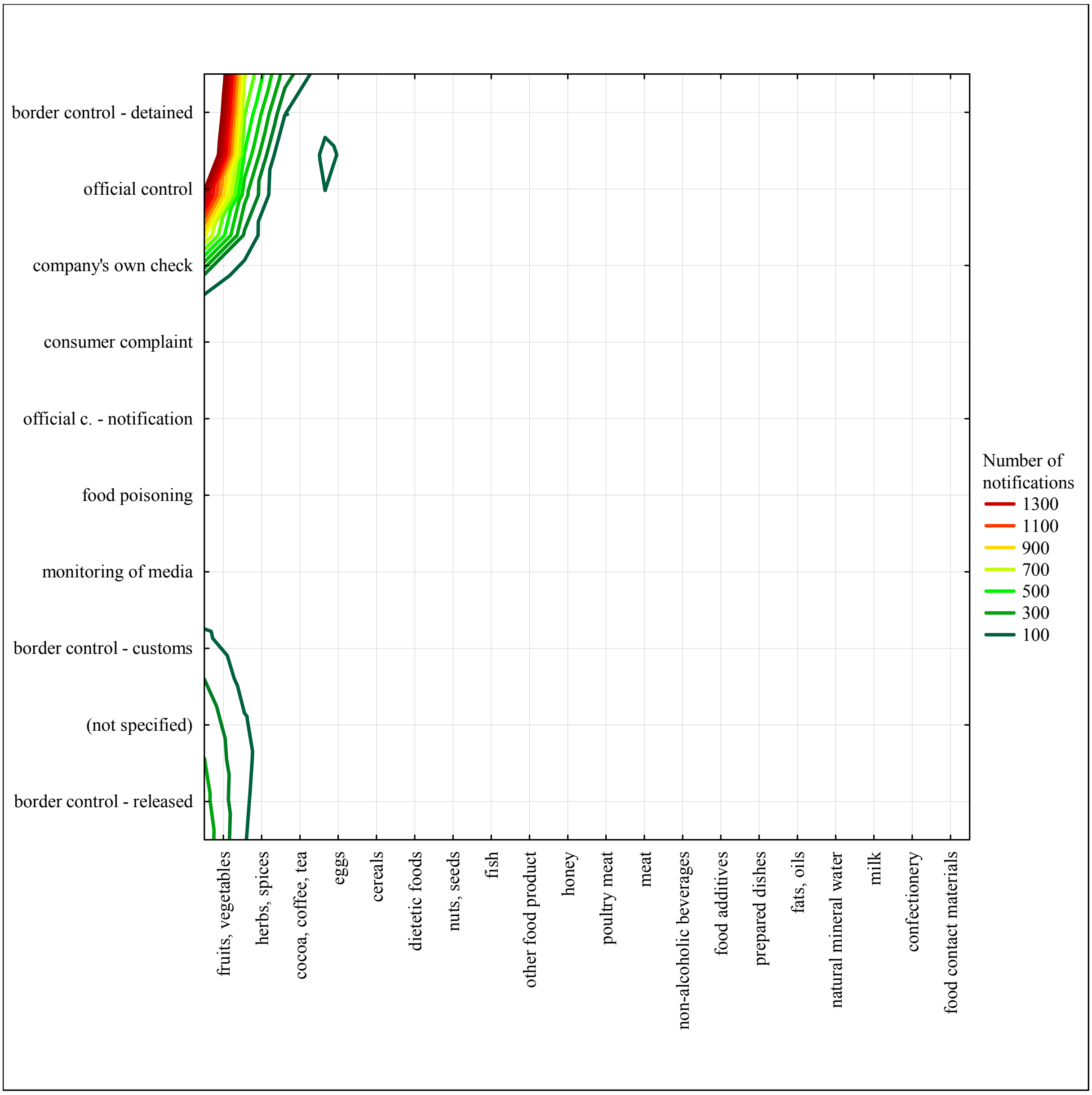

cereals – cereals and bakery products
cocoa, coffee, tea – cocoa and cocoa preparations, coffee and tea
dietetic foods – dietetic foods, food supplements, fortified foods
eggs – eggs and egg products
fats, oils – fats and oils
fish – fish and fish products
food additives – food additives and flavourings
fruits, vegetables – fruits and vegetables
herbs, spices – herbs and spices
honey – honey and royal jelly
meat – meat and meat products (other than poultry)
milk – milk and milk products
nuts, seeds – nuts, nut products and seeds
other food product – other food product / mixed
poultry meat – poultry meat and poultry meat products
prepared dishes – prepared dishes and snacks

border control - customs – border control - consignment under customs
border control - detained – border control - consignment detained
border control - released – border control - consignment released
official c. - notification – official control following RASFF notification
official control – official control on the market

FIGURE S29 Similarities between product category and notification basis within notifications on pesticide residues.

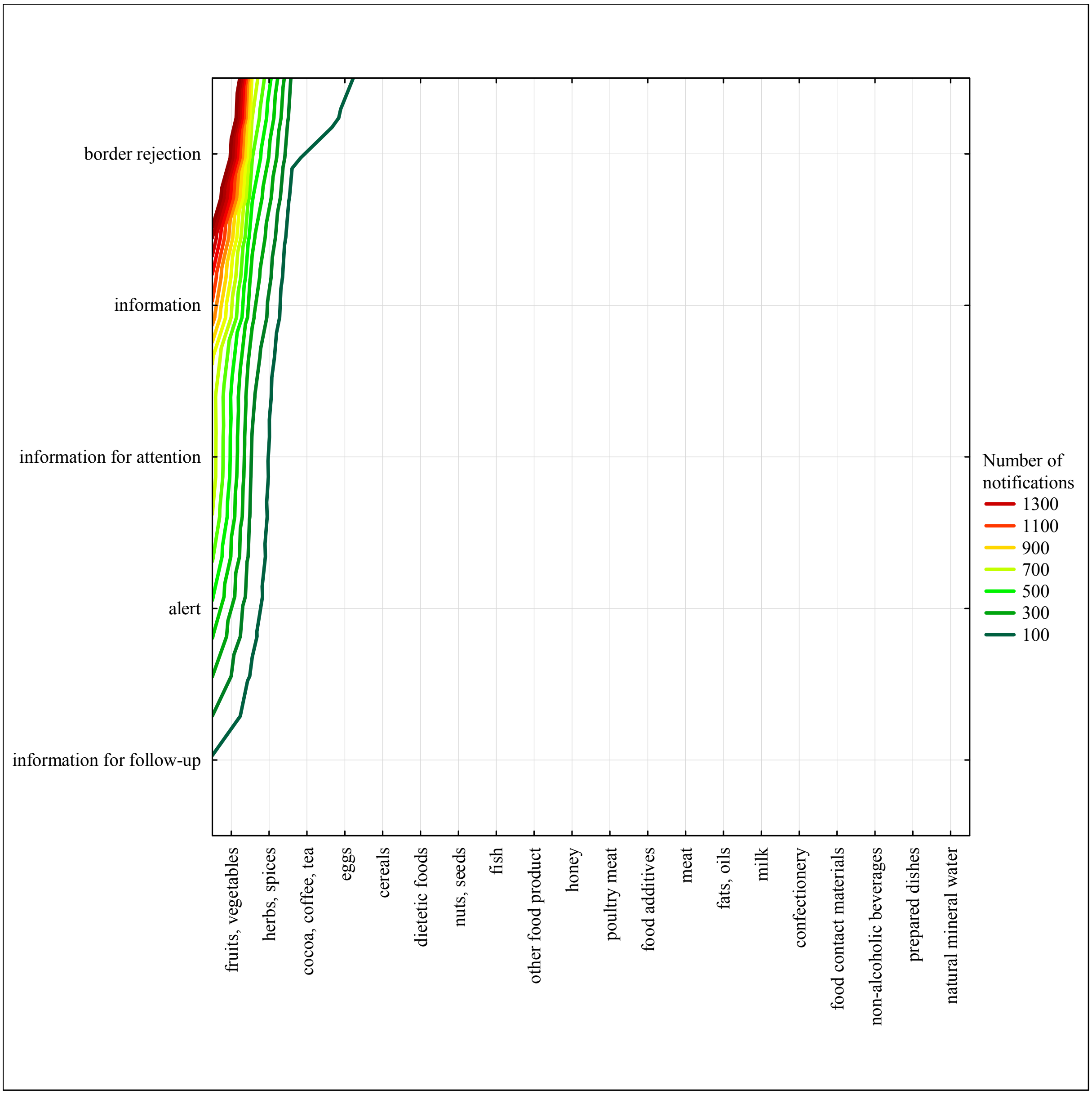

cereals – cereals and bakery products
cocoa, coffee, tea – cocoa and cocoa preparations, coffee and tea
dietetic foods – dietetic foods, food supplements, fortified foods
eggs – eggs and egg products
fats, oils – fats and oils
fish – fish and fish products
food additives – food additives and flavourings
fruits, vegetables – fruits and vegetables
herbs, spices – herbs and spices
honey – honey and royal jelly
meat – meat and meat products (other than poultry)
milk – milk and milk products
nuts, seeds – nuts, nut products and seeds
other food product – other food product / mixed
poultry meat – poultry meat and poultry meat products
prepared dishes – prepared dishes and snacks

FIGURE S30 Similarities between product category and notification type within notifications on pesticide residues.

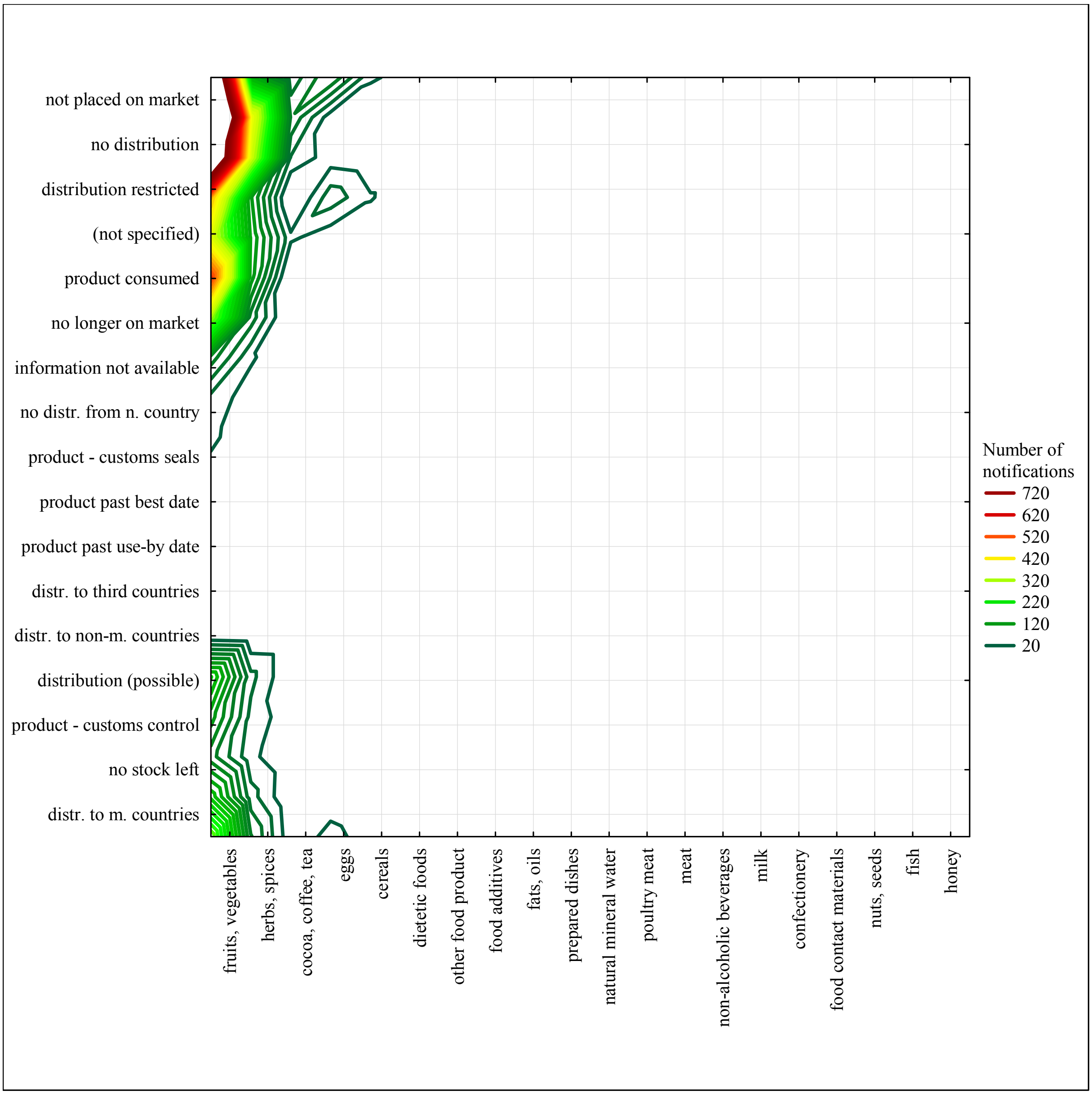

cereals – cereals and bakery products
cocoa, coffee, tea – cocoa and cocoa preparations, coffee and tea
dietetic foods – dietetic foods, food supplements, fortified foods
eggs – eggs and egg products
fats, oils – fats and oils
fish – fish and fish products
food additives – food additives and flavourings
fruits, vegetables – fruits and vegetables
herbs, spices – herbs and spices
honey – honey and royal jelly
meat – meat and meat products (other than poultry)
milk – milk and milk products
nuts, seeds – nuts, nut products and seeds
other food product – other food product / mixed
poultry meat – poultry meat and poultry meat products
prepared dishes – prepared dishes and snacks

distr. to m. countries – distribution to other member countries
distr. to non-m. countries – distribution to non-member countries
distr. to third countries – distribution to third countries
distribution (possible) – distribution on the market (possible)
distribution restricted – distribution restricted to notifying country
information not available – information on distribution not (yet) available
no distr. from n. country – no distribution from notifying country
no longer on market – product (presumably) no longer on the market
not placed on market – product not (yet) placed on the market
product - customs control – product under customs control
product - customs seals – product allowed to travel to destination under customs seals
product consumed – product already consumed
product past best date – product past best before date

FIGURE S31 Similarities between product category and distribution status within notifications on pesticide residues.

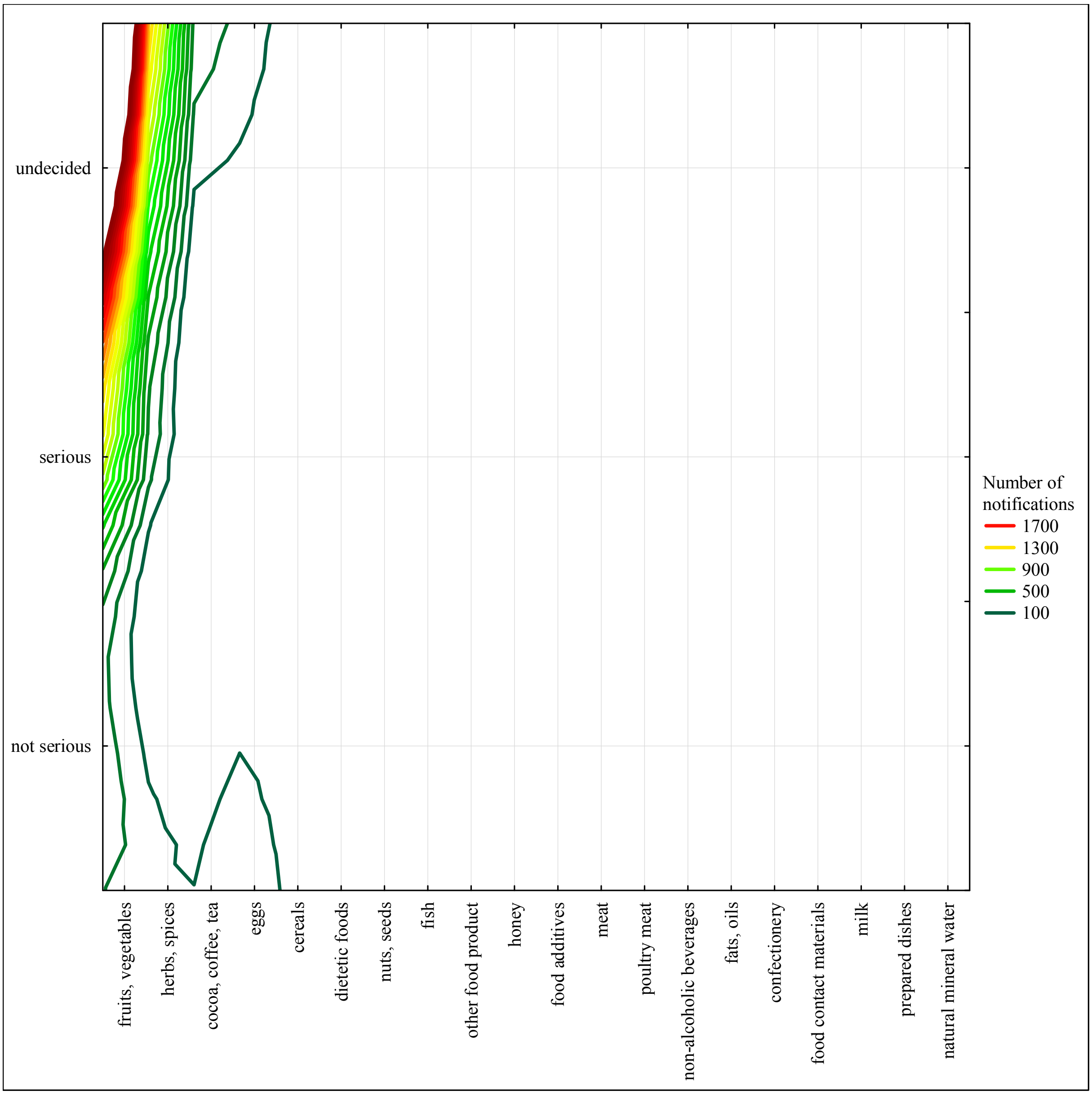

cereals – cereals and bakery products
cocoa, coffee, tea – cocoa and cocoa preparations, coffee and tea
dietetic foods – dietetic foods, food supplements, fortified foods
eggs – eggs and egg products
fats, oils – fats and oils
fish – fish and fish products
food additives – food additives and flavourings
fruits, vegetables – fruits and vegetables
herbs, spices – herbs and spices
honey – honey and royal jelly
meat – meat and meat products (other than poultry)
milk – milk and milk products
nuts, seeds – nuts, nut products and seeds
other food product – other food product / mixed
poultry meat – poultry meat and poultry meat products
prepared dishes – prepared dishes and snacks

FIGURE S32 Similarities between product category and risk decision within notifications on pesticide residues.

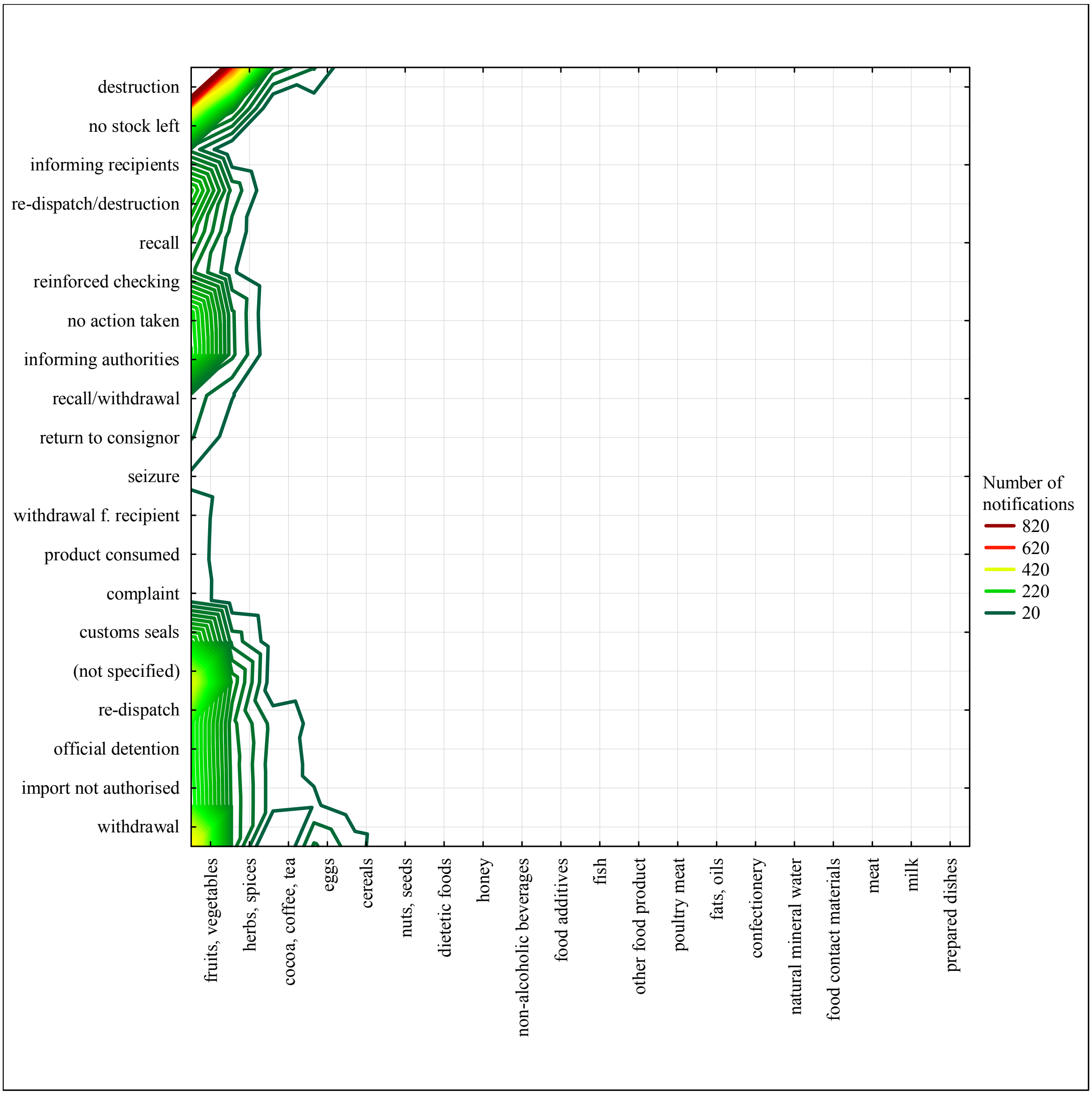

cereals – cereals and bakery products
cocoa, coffee, tea – cocoa and cocoa preparations, coffee and tea
dietetic foods – dietetic foods, food supplements, fortified foods
eggs – eggs and egg products
fats, oils – fats and oils
fish – fish and fish products
food additives – food additives and flavourings
fruits, vegetables – fruits and vegetables
herbs, spices – herbs and spices
honey – honey and royal jelly
meat – meat and meat products (other than poultry)
milk – milk and milk products
nuts, seeds – nuts, nut products and seeds
other food product – other food product / mixed
poultry meat – poultry meat and poultry meat products
prepared dishes – prepared dishes and snacks

customs seals – placed under customs seals
product consumed – product already consumed
recall – recall from consumers
recall/withdrawal – product recall or withdrawal
re-dispatch/destruction – re-dispatch or destruction
withdrawal – withdrawal from the market
withdrawal f. recipient – withdrawal from recipient(s)

FIGURE S33 Similarities between product category and action taken within notifications on pesticide residues.

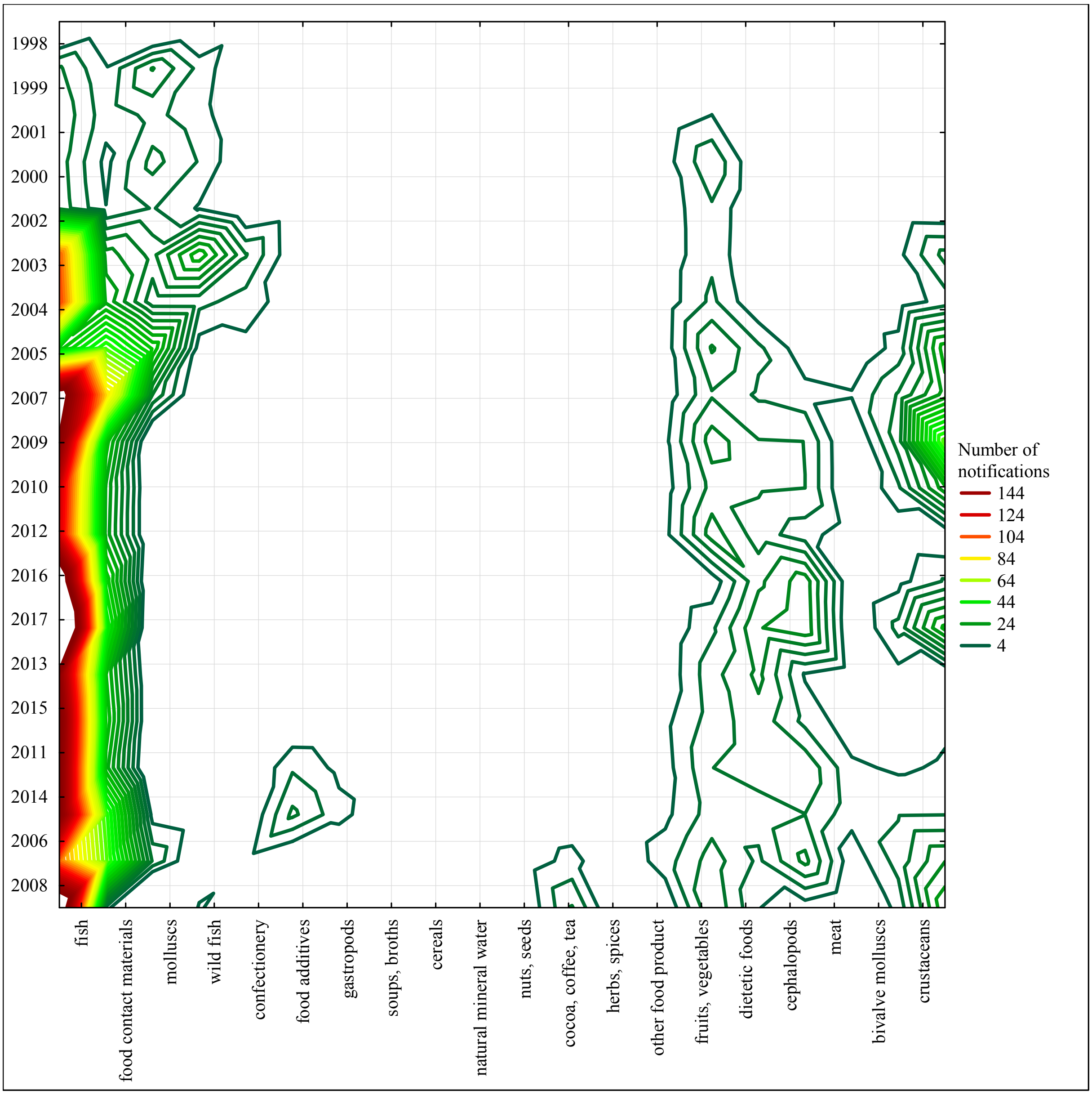

bivalve molluscs – bivalve molluscs and products thereof
cephalopods – cephalopods and products thereof
cereals – cereals and bakery products
cocoa, coffee, tea – cocoa and cocoa preparations, coffee and tea
crustaceans – crustaceans and products thereof
dietetic foods – dietetic foods, food supplements, fortified foods
fish – fish and fish products
food additives – food additives and flavourings
fruits, vegetables – fruits and vegetables
herbs, spices – herbs and spices
meat – meat and meat products (other than poultry)
molluscs – molluscs and products thereof - (obsolete)
nuts, seeds – nuts, nut products and seeds
other food product – other food product / mixed
soups, broths – soups, broths, sauces and condiments
wild fish – wild caught fish and products thereof (other than crustaceans and molluscs) - (obsolete)

FIGURE S34 Similarities between product category and year within notifications on heavy metals.

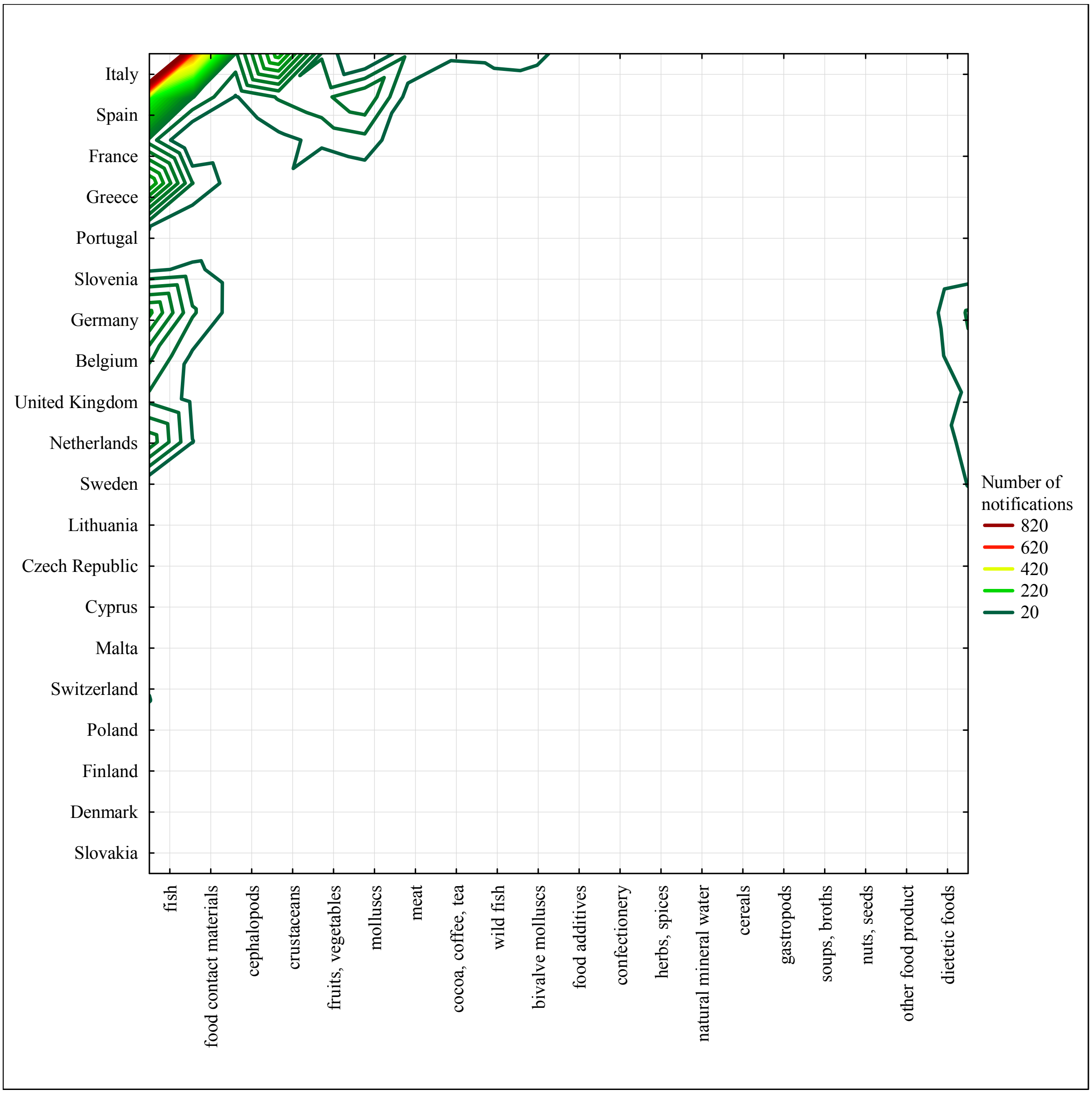

bivalve molluscs – bivalve molluscs and products thereof
cephalopods – cephalopods and products thereof
cereals – cereals and bakery products
cocoa, coffee, tea – cocoa and cocoa preparations, coffee and tea
crustaceans – crustaceans and products thereof
dietetic foods – dietetic foods, food supplements, fortified foods
fish – fish and fish products
food additives – food additives and flavourings
fruits, vegetables – fruits and vegetables
herbs, spices – herbs and spices
meat – meat and meat products (other than poultry)
molluscs – molluscs and products thereof - (obsolete)
nuts, seeds – nuts, nut products and seeds
other food product – other food product / mixed
soups, broths – soups, broths, sauces and condiments
wild fish – wild caught fish and products thereof (other than crustaceans and molluscs) - (obsolete)

FIGURE S35 Similarities between product category and notifying country within notifications on heavy metals.

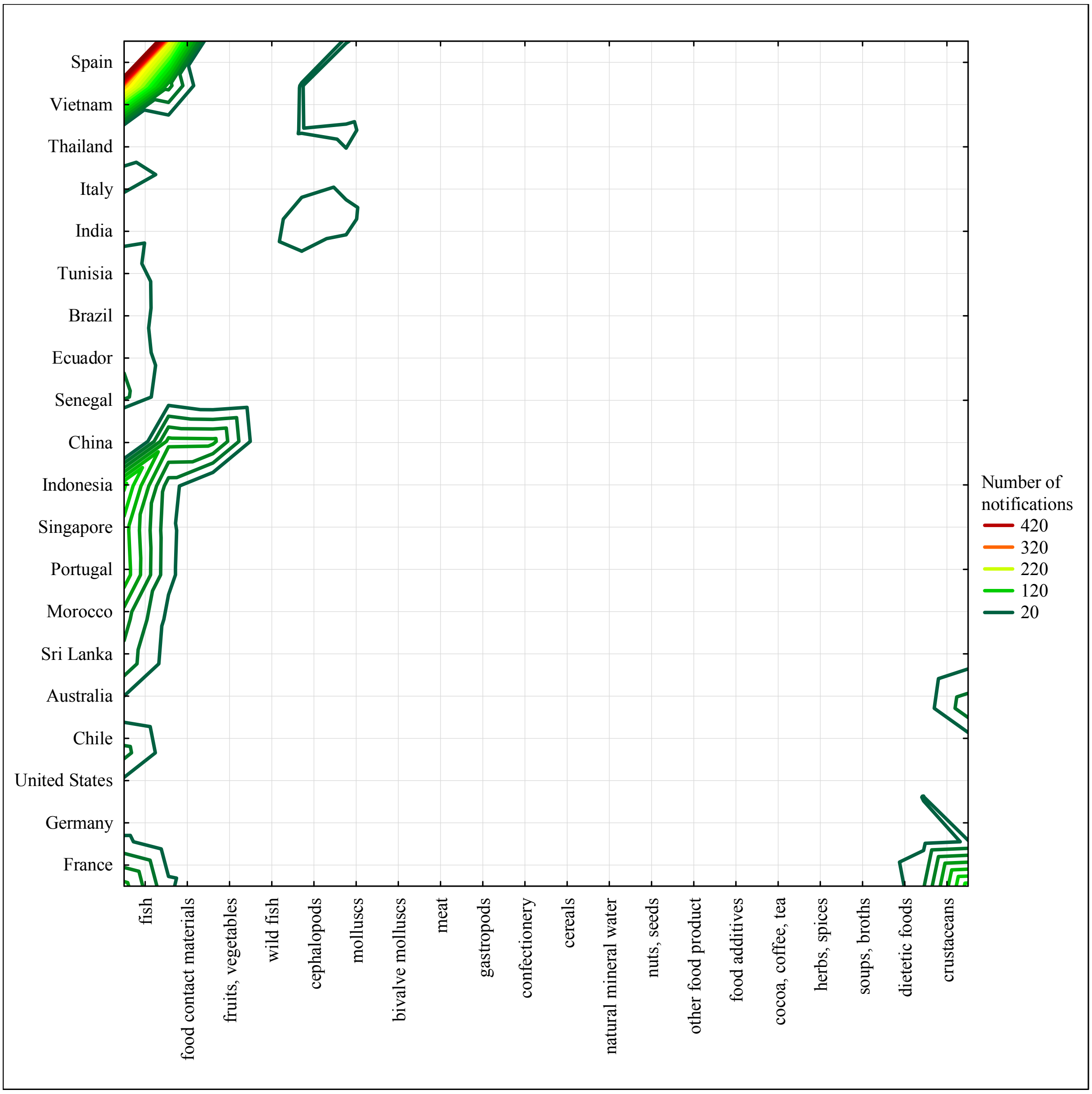

bivalve molluscs – bivalve molluscs and products thereof
cephalopods – cephalopods and products thereof
cereals – cereals and bakery products
cocoa, coffee, tea – cocoa and cocoa preparations, coffee and tea
crustaceans – crustaceans and products thereof
dietetic foods – dietetic foods, food supplements, fortified foods
fish – fish and fish products
food additives – food additives and flavourings
fruits, vegetables – fruits and vegetables
herbs, spices – herbs and spices
meat – meat and meat products (other than poultry)
molluscs – molluscs and products thereof - (obsolete)
nuts, seeds – nuts, nut products and seeds
other food product – other food product / mixed
soups, broths – soups, broths, sauces and condiments
wild fish – wild caught fish and products thereof (other than crustaceans and molluscs) - (obsolete)

FIGURE S36 Similarities between product category and origin country within notifications on heavy metals.

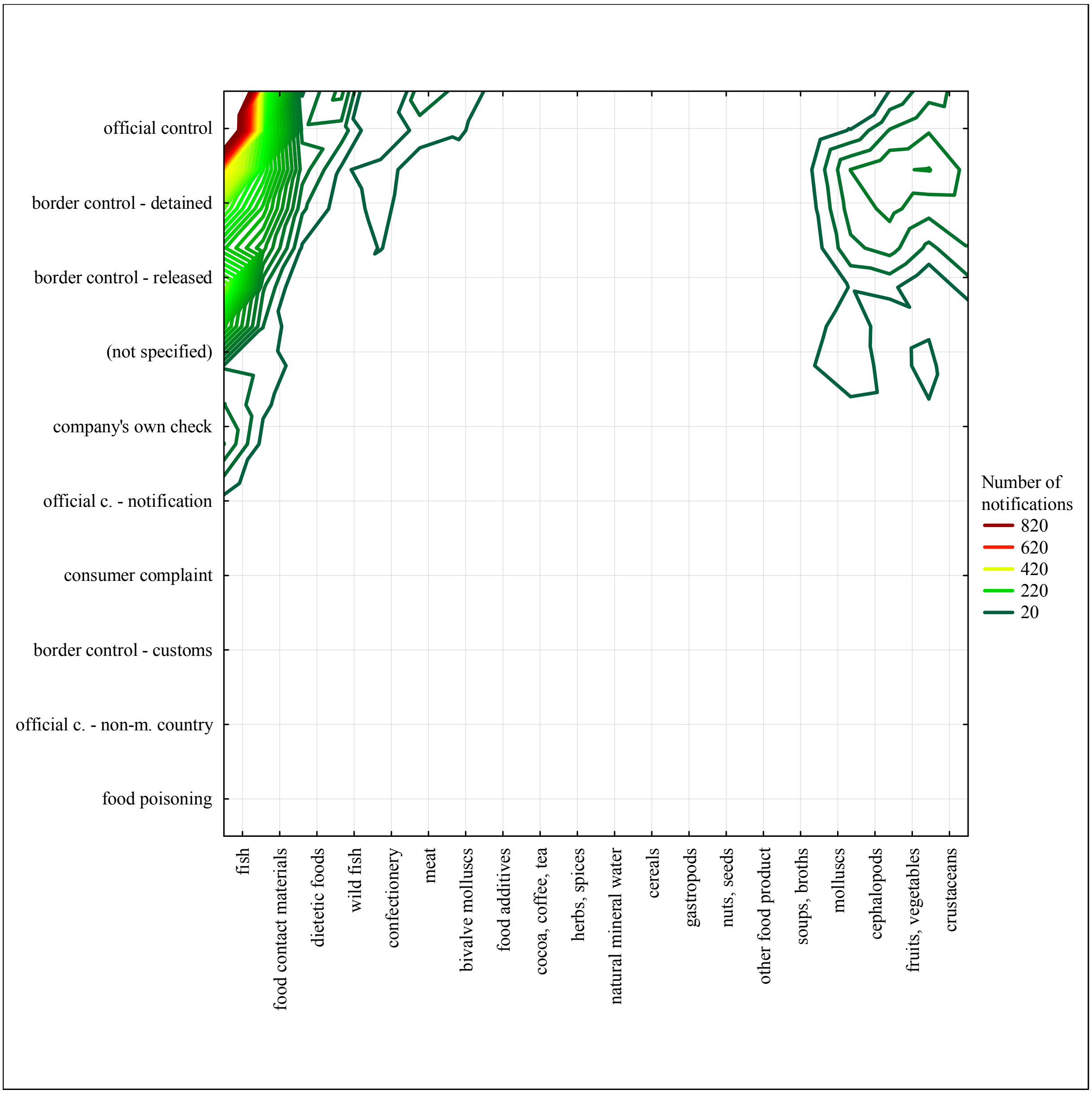

bivalve molluscs – bivalve molluscs and products thereof
cephalopods – cephalopods and products thereof
cereals – cereals and bakery products
cocoa, coffee, tea – cocoa and cocoa preparations, coffee and tea
crustaceans – crustaceans and products thereof
dietetic foods – dietetic foods, food supplements, fortified foods
fish – fish and fish products
food additives – food additives and flavourings
fruits, vegetables – fruits and vegetables
herbs, spices – herbs and spices
meat – meat and meat products (other than poultry)
molluscs – molluscs and products thereof - (obsolete)
nuts, seeds – nuts, nut products and seeds
other food product – other food product / mixed
soups, broths – soups, broths, sauces and condiments
wild fish – wild caught fish and products thereof (other than crustaceans and molluscs) - (obsolete)

border control - customs – border control - consignment under customs
border control - detained – border control - consignment detained
border control - released – border control - consignment released
official c. - notification – official control following RASFF notification
official c. – non-m. country – official control in non-member country
official control – official control on the market

FIGURE S37 Similarities between product category and notification basis within notifications on heavy metals.

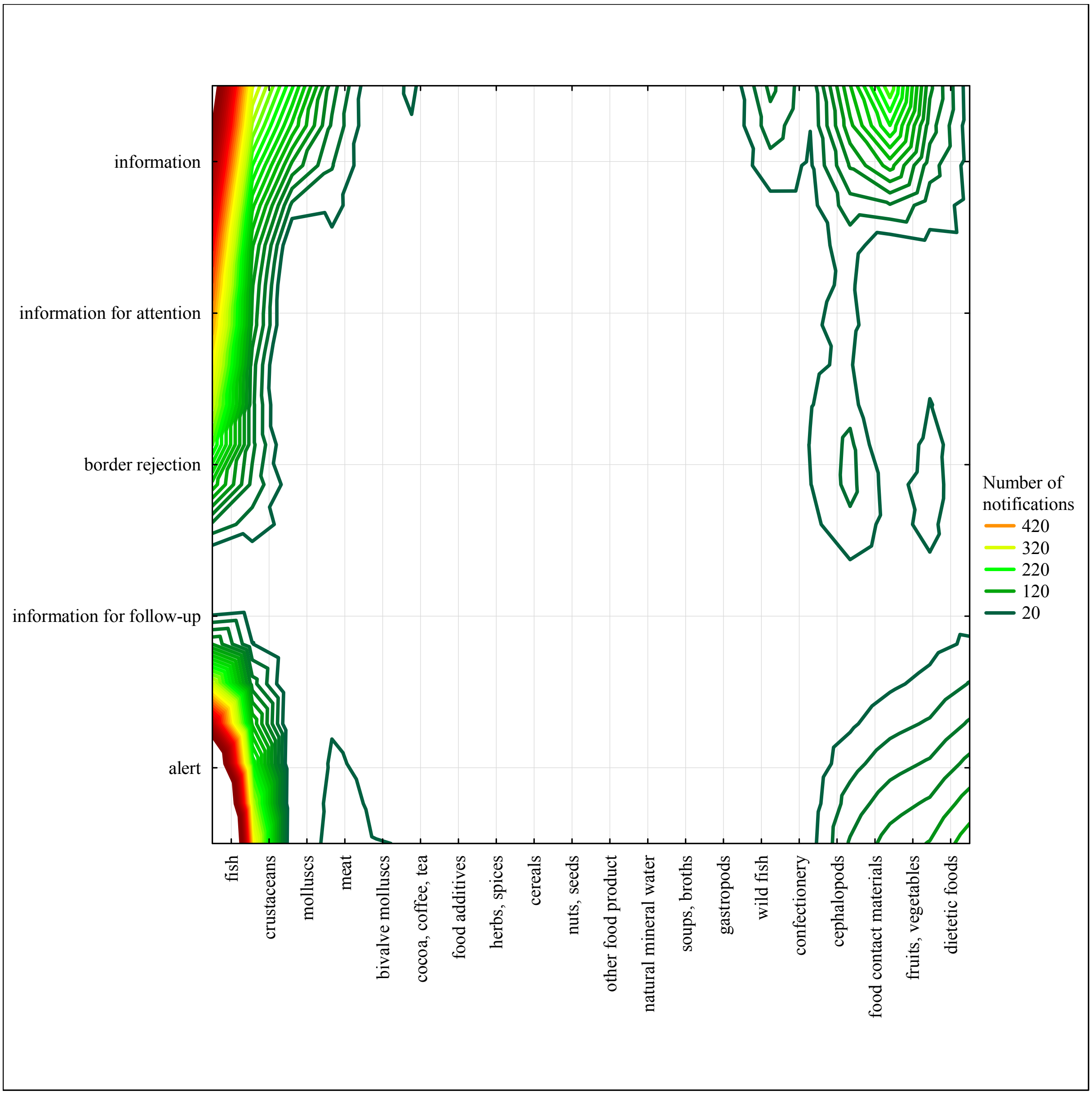

bivalve molluscs – bivalve molluscs and products thereof
cephalopods – cephalopods and products thereof
cereals – cereals and bakery products
cocoa, coffee, tea – cocoa and cocoa preparations, coffee and tea
crustaceans – crustaceans and products thereof
dietetic foods – dietetic foods, food supplements, fortified foods
fish – fish and fish products
food additives – food additives and flavourings
fruits, vegetables – fruits and vegetables
herbs, spices – herbs and spices
meat – meat and meat products (other than poultry)
molluscs – molluscs and products thereof - (obsolete)
nuts, seeds – nuts, nut products and seeds
other food product – other food product / mixed
soups, broths – soups, broths, sauces and condiments
wild fish – wild caught fish and products thereof (other than crustaceans and molluscs) - (obsolete)

FIGURE S38 Similarities between product category and notification type within notifications on heavy metals.

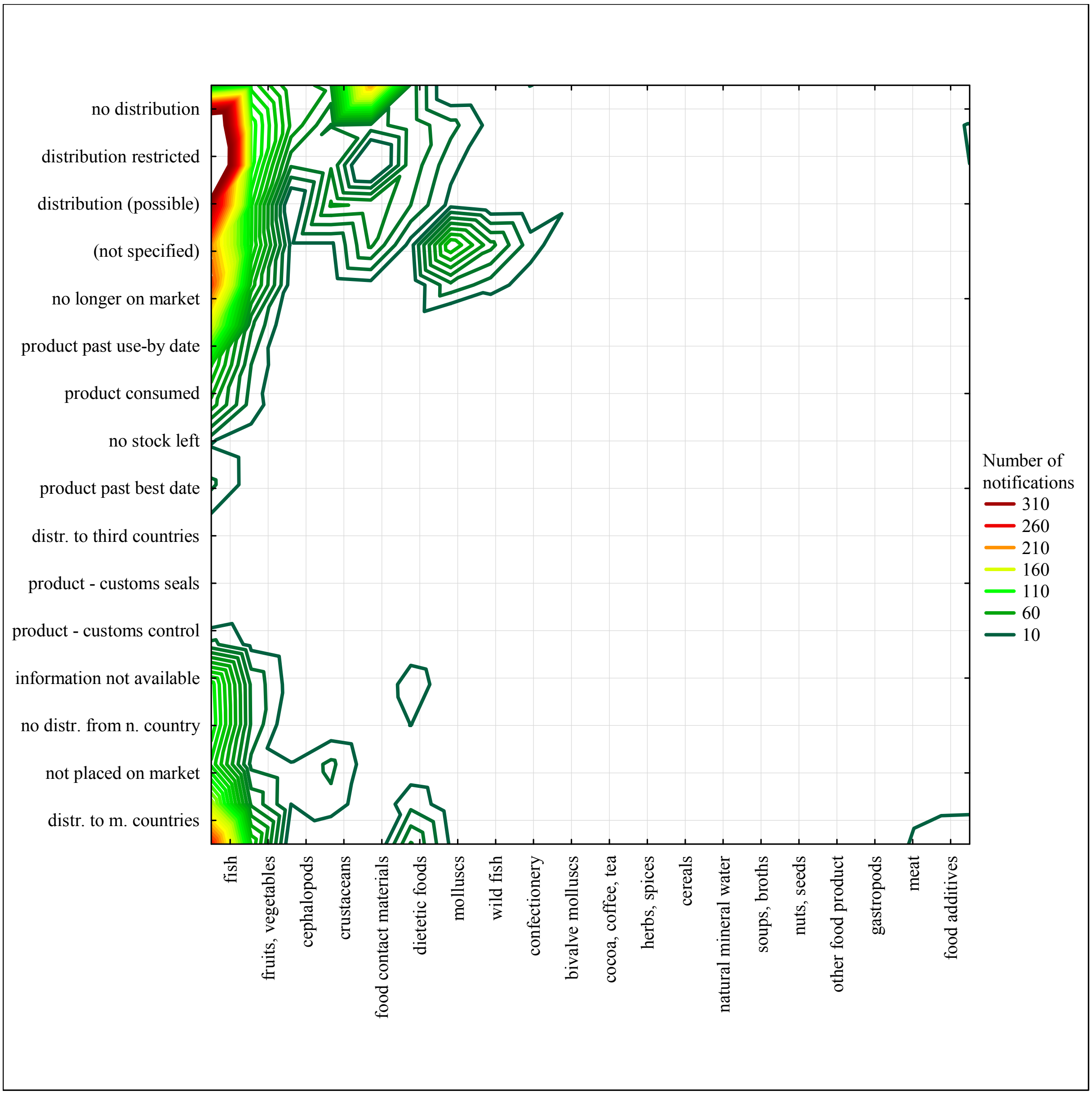

bivalve molluscs – bivalve molluscs and products thereof
cephalopods – cephalopods and products thereof
cereals – cereals and bakery products
cocoa, coffee, tea – cocoa and cocoa preparations, coffee and tea
crustaceans – crustaceans and products thereof
dietetic foods – dietetic foods, food supplements, fortified foods
fish – fish and fish products
food additives – food additives and flavourings
fruits, vegetables – fruits and vegetables
herbs, spices – herbs and spices
meat – meat and meat products (other than poultry)
molluscs – molluscs and products thereof - (obsolete)
nuts, seeds – nuts, nut products and seeds
other food product – other food product / mixed
soups, broths – soups, broths, sauces and condiments
wild fish – wild caught fish and products thereof (other than crustaceans and molluscs) - (obsolete)

distr. to m. countries – distribution to other member countries
distr. to third countries – distribution to third countries
distribution (possible) – distribution on the market (possible)
distribution restricted – distribution restricted to notifying country
information not available – information on distribution not (yet) available
no distr. from n. country – no distribution from notifying country
no longer on market – product (presumably) no longer on the market
not placed on market – product not (yet) placed on the market
product - customs control – product under customs control
product - customs seals – product allowed to travel to destination under customs seals
product consumed – product already consumed
product past best date – product past best before date

FIGURE S39 Similarities between product category and distribution status within notifications on heavy metals.

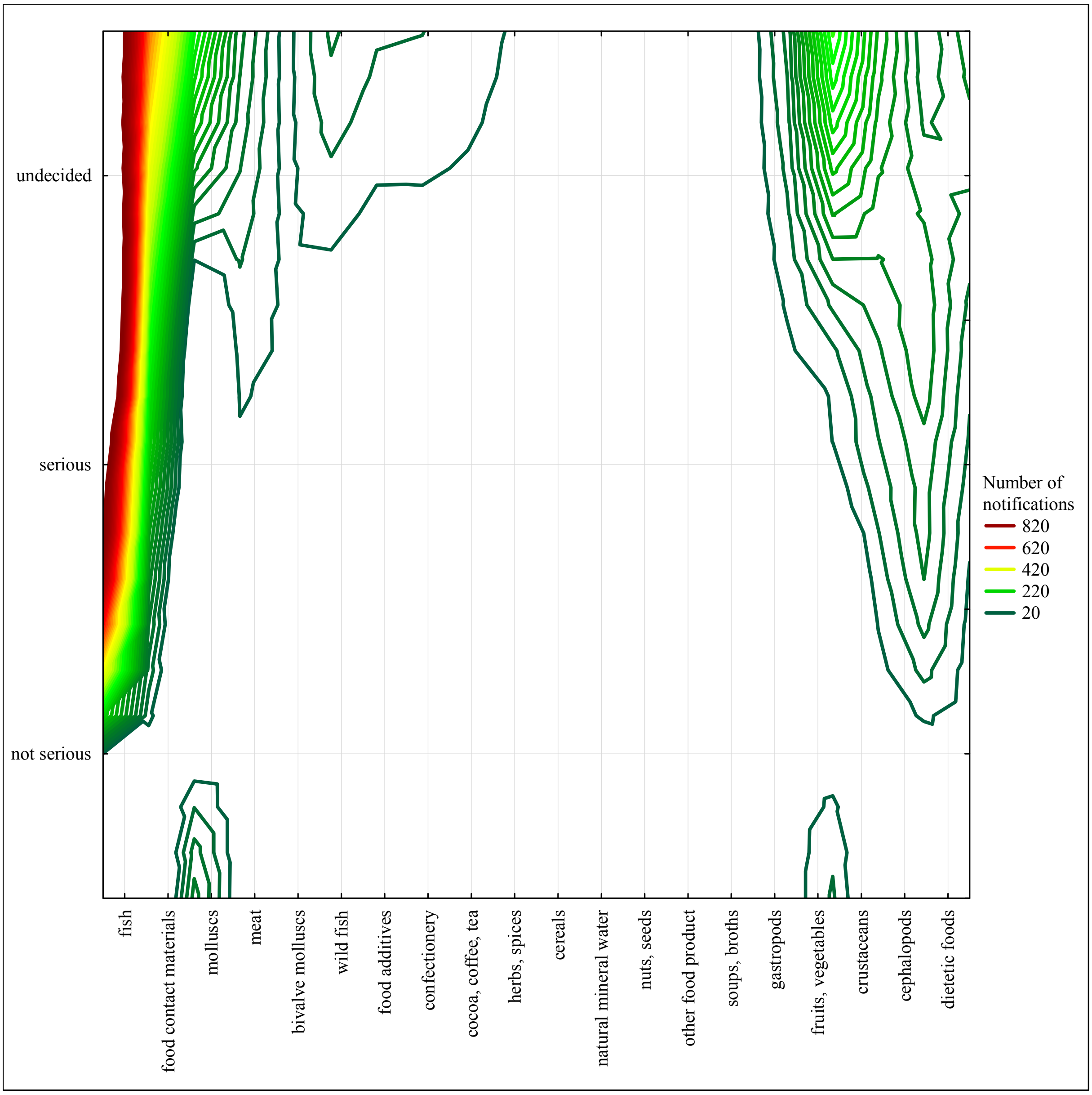

bivalve molluscs – bivalve molluscs and products thereof
cephalopods – cephalopods and products thereof
cereals – cereals and bakery products
cocoa, coffee, tea – cocoa and cocoa preparations, coffee and tea
crustaceans – crustaceans and products thereof
dietetic foods – dietetic foods, food supplements, fortified foods
fish – fish and fish products
food additives – food additives and flavourings
fruits, vegetables – fruits and vegetables
herbs, spices – herbs and spices
meat – meat and meat products (other than poultry)
molluscs – molluscs and products thereof - (obsolete)
nuts, seeds – nuts, nut products and seeds
other food product – other food product / mixed
soups, broths – soups, broths, sauces and condiments
wild fish – wild caught fish and products thereof (other than crustaceans and molluscs) - (obsolete)

FIGURE S40 Similarities between product category and risk decision within notifications on heavy metals.

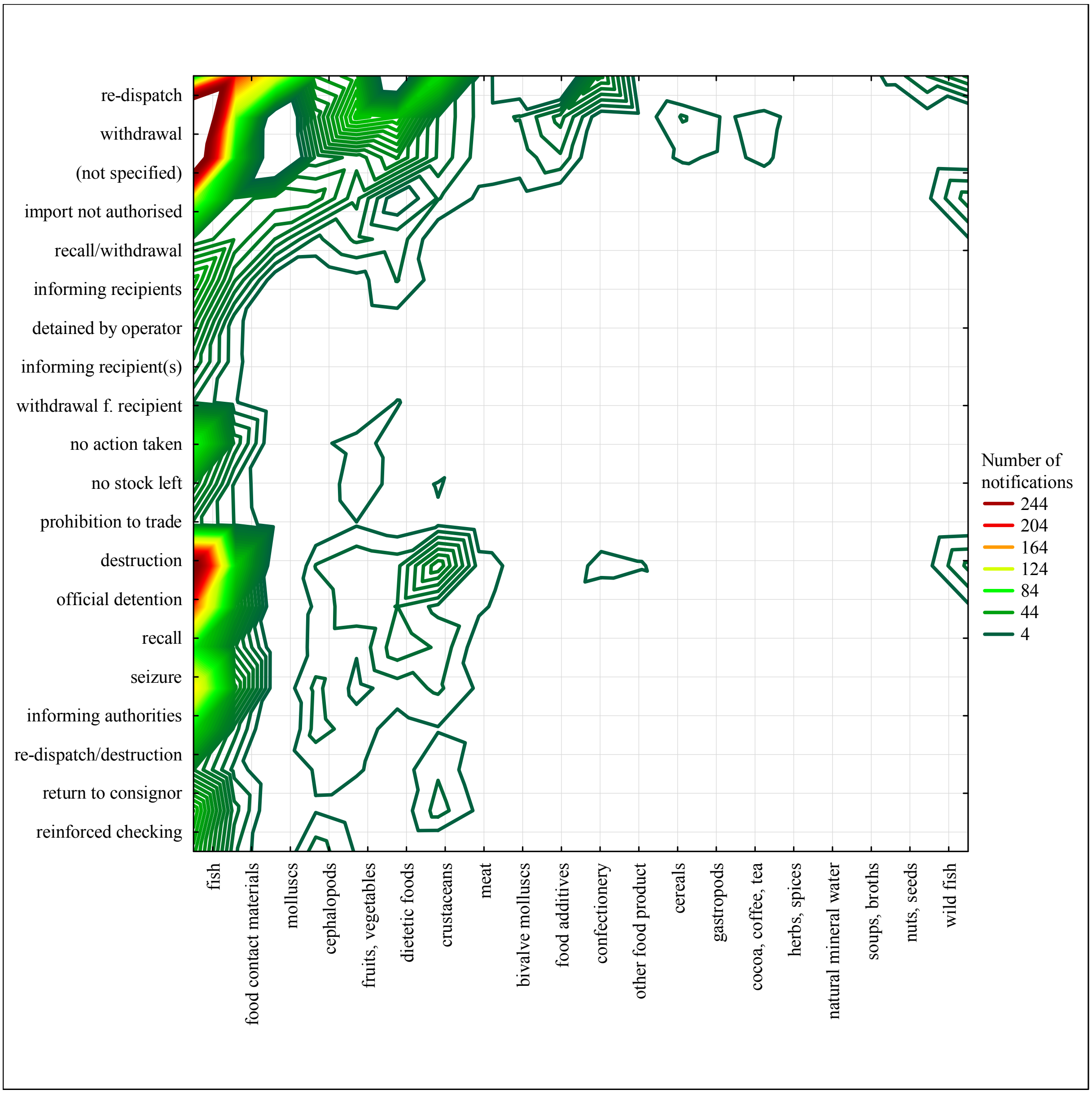

bivalve molluscs – bivalve molluscs and products thereof
cephalopods – cephalopods and products thereof
cereals – cereals and bakery products
cocoa, coffee, tea – cocoa and cocoa preparations, coffee and tea
crustaceans – crustaceans and products thereof
dietetic foods – dietetic foods, food supplements, fortified foods
fish – fish and fish products
food additives – food additives and flavourings
fruits, vegetables – fruits and vegetables
herbs, spices – herbs and spices
meat – meat and meat products (other than poultry)
molluscs – molluscs and products thereof - (obsolete)
nuts, seeds – nuts, nut products and seeds
other food product – other food product / mixed
soups, broths – soups, broths, sauces and condiments
wild fish – wild caught fish and products thereof (other than crustaceans and molluscs) - (obsolete)

prohibition to trade – prohibition to trade - sales ban
recall – recall from consumers
recall/withdrawal – product recall or withdrawal
re-dispatch/destruction – re-dispatch or destruction
withdrawal – withdrawal from the market
withdrawal f. recipient – withdrawal from recipient(s)

FIGURE S41 Similarities between product category and action taken within notifications on heavy metals.

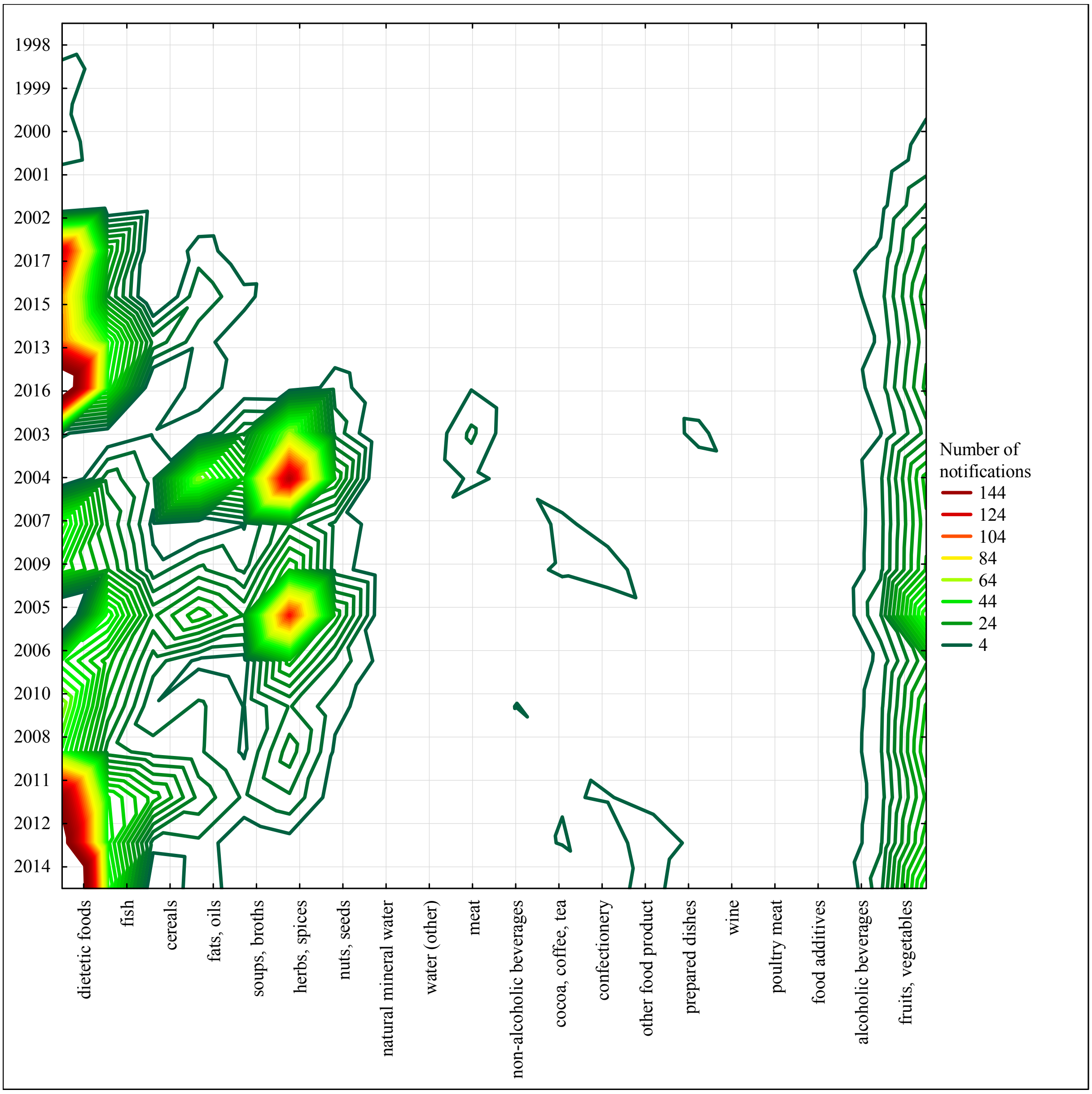

cereals – cereals and bakery products
cocoa, coffee, tea – cocoa and cocoa preparations, coffee and tea
dietetic foods – dietetic foods, food supplements, fortified foods
fats, oils – fats and oils
fish – fish and fish products
food additives – food additives and flavourings
fruits, vegetables – fruits and vegetables
herbs, spices – herbs and spices
meat – meat and meat products (other than poultry)
nuts, seeds – nuts, nut products and seeds
other food product – other food product / mixed
poultry meat – poultry meat and poultry meat products
prepared dishes – prepared dishes and snacks
soups, broths – soups, broths, sauces and condiments
water (other) – water for human consumption (other)

FIGURE S42 Similarities between product category and year within notifications on composition.

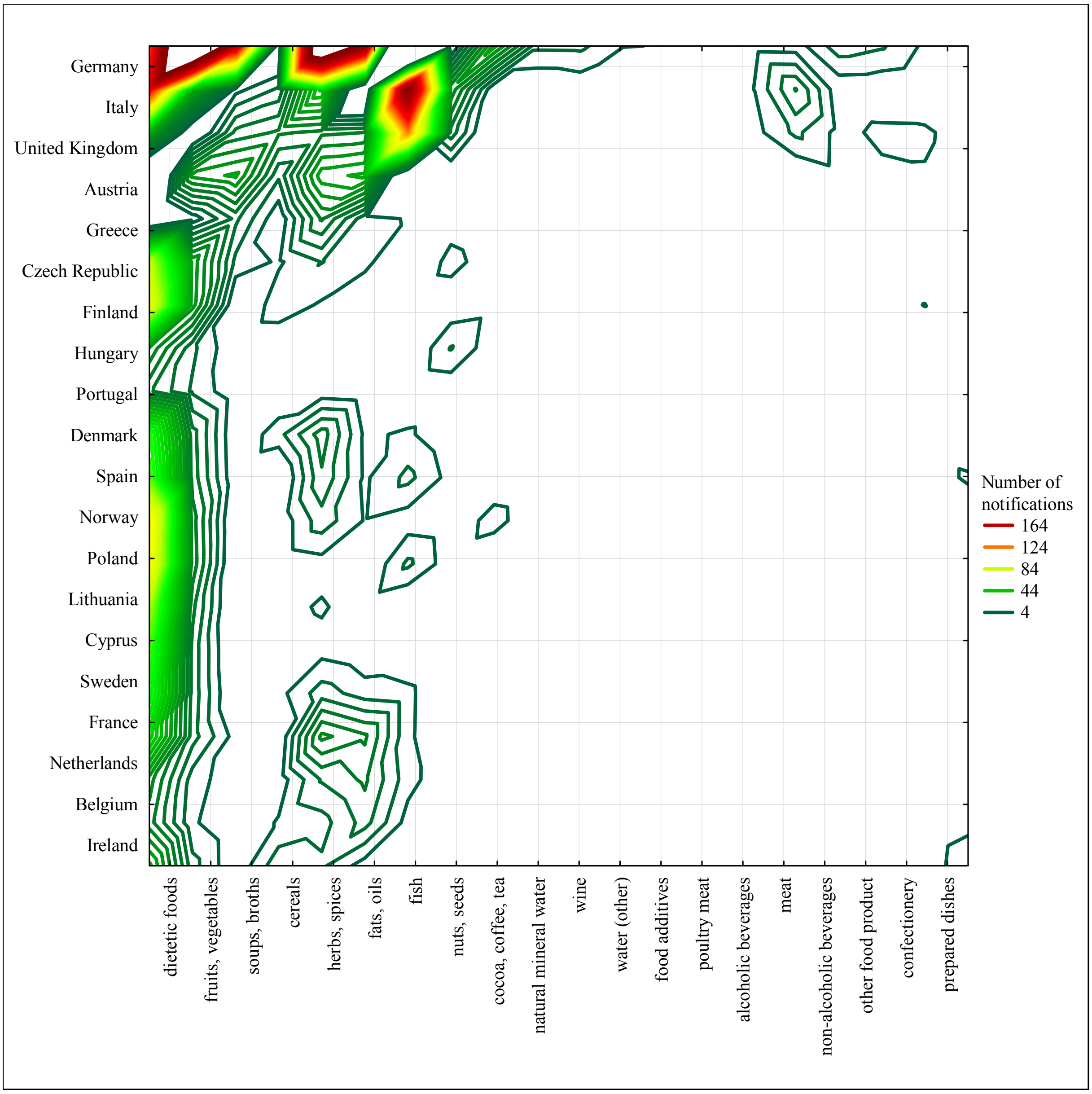

cereals – cereals and bakery products
cocoa, coffee, tea – cocoa and cocoa preparations, coffee and tea
dietetic foods – dietetic foods, food supplements, fortified foods
fats, oils – fats and oils
fish – fish and fish products
food additives – food additives and flavourings
fruits, vegetables – fruits and vegetables
herbs, spices – herbs and spices
meat – meat and meat products (other than poultry)
nuts, seeds – nuts, nut products and seeds
other food product – other food product / mixed
poultry meat – poultry meat and poultry meat products
prepared dishes – prepared dishes and snacks
soups, broths – soups, broths, sauces and condiments
water (other) – water for human consumption (other)

FIGURE S43 Similarities between product category and notifying country within notifications on composition.

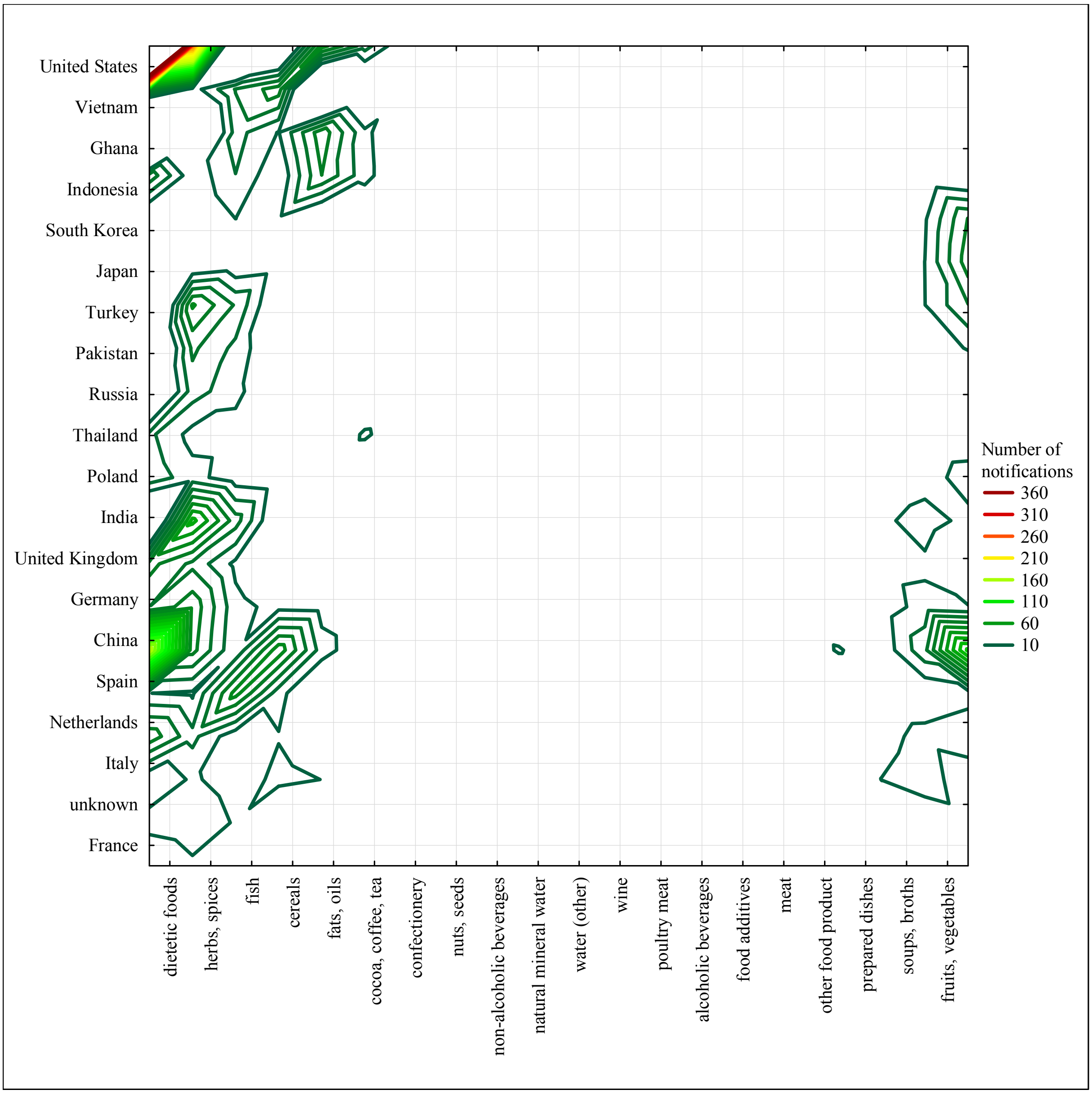

cereals – cereals and bakery products
cocoa, coffee, tea – cocoa and cocoa preparations, coffee and tea
dietetic foods – dietetic foods, food supplements, fortified foods
fats, oils – fats and oils
fish – fish and fish products
food additives – food additives and flavourings
fruits, vegetables – fruits and vegetables
herbs, spices – herbs and spices
meat – meat and meat products (other than poultry)
nuts, seeds – nuts, nut products and seeds
other food product – other food product / mixed
poultry meat – poultry meat and poultry meat products
prepared dishes – prepared dishes and snacks
soups, broths – soups, broths, sauces and condiments
water (other) – water for human consumption (other)

FIGURE S44 Similarities between product category and origin country within notifications on composition.

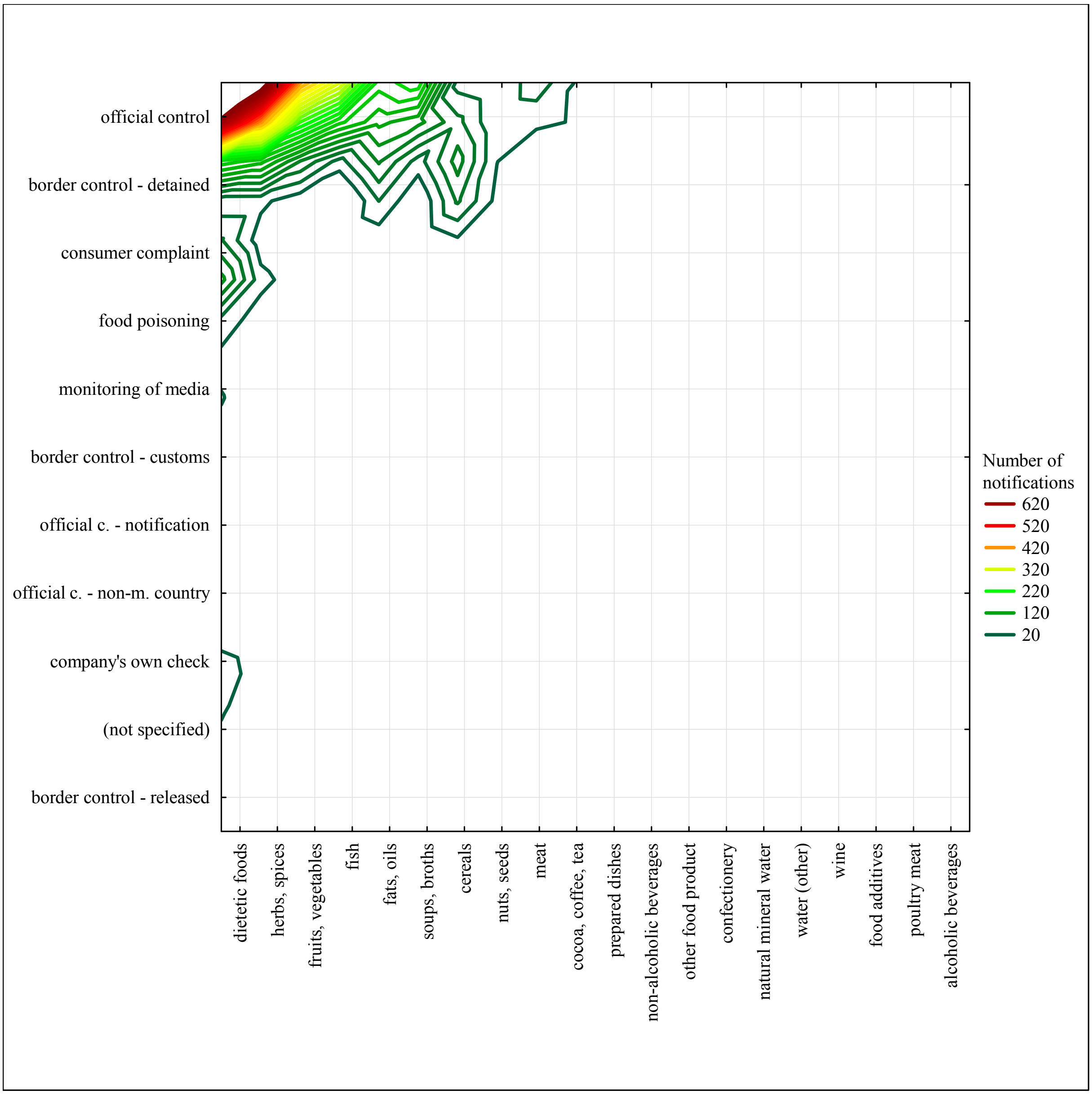

cereals – cereals and bakery products
cocoa, coffee, tea – cocoa and cocoa preparations, coffee and tea
dietetic foods – dietetic foods, food supplements, fortified foods
fats, oils – fats and oils
fish – fish and fish products
food additives – food additives and flavourings
fruits, vegetables – fruits and vegetables
herbs, spices – herbs and spices
meat – meat and meat products (other than poultry)
nuts, seeds – nuts, nut products and seeds
other food product – other food product / mixed
poultry meat – poultry meat and poultry meat products
prepared dishes – prepared dishes and snacks
soups, broths – soups, broths, sauces and condiments
water (other) – water for human consumption (other)

border control - customs – border control - consignment under customs
border control - detained – border control - consignment detained
border control - released – border control - consignment released
official c. - non-m. country – official control in non-member country
official c. - notification – official control following RASFF notification
official control – official control on the market

FIGURE S45 Similarities between product category and notification basis within notifications on composition.

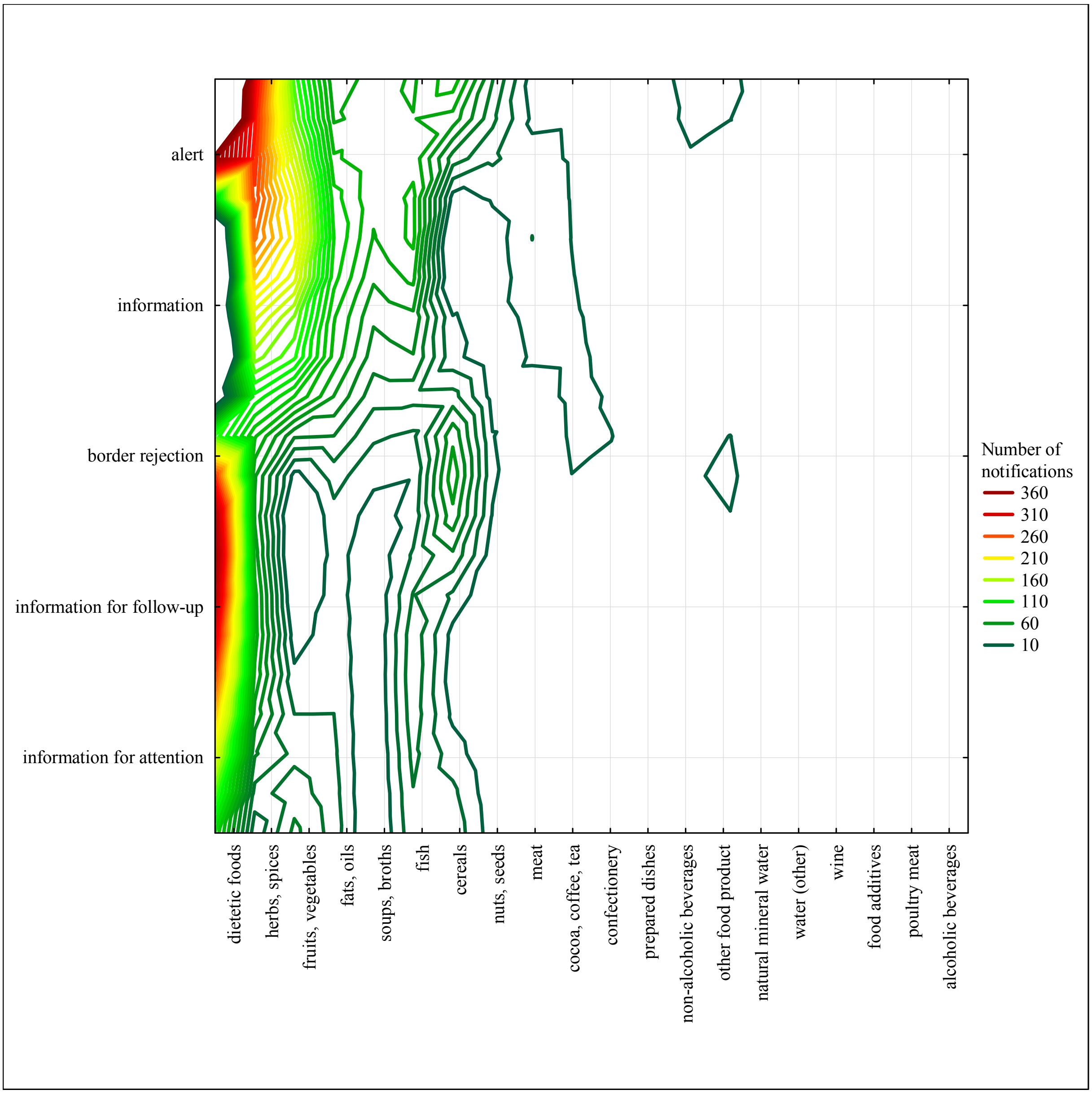

cereals – cereals and bakery products
cocoa, coffee, tea – cocoa and cocoa preparations, coffee and tea
dietetic foods – dietetic foods, food supplements, fortified foods
fats, oils – fats and oils
fish – fish and fish products
food additives – food additives and flavourings
fruits, vegetables – fruits and vegetables
herbs, spices – herbs and spices
meat – meat and meat products (other than poultry)
nuts, seeds – nuts, nut products and seeds
other food product – other food product / mixed
poultry meat – poultry meat and poultry meat products
prepared dishes – prepared dishes and snacks
soups, broths – soups, broths, sauces and condiments
water (other) – water for human consumption (other)

FIGURE S46 Similarities between product category and notification type within notifications on composition.

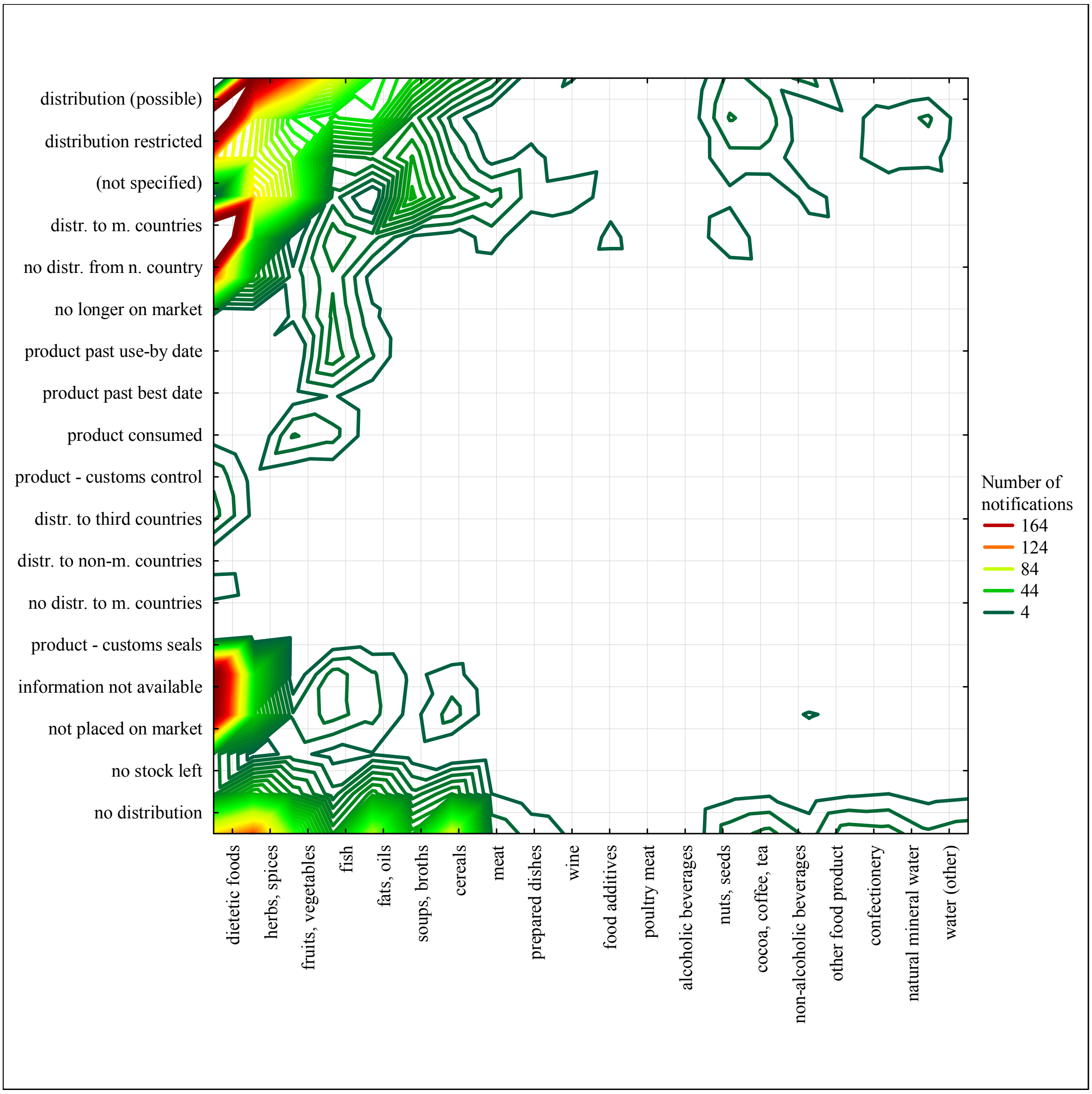

cereals – cereals and bakery products
cocoa, coffee, tea – cocoa and cocoa preparations, coffee and tea
dietetic foods – dietetic foods, food supplements, fortified foods
fats, oils – fats and oils
fish – fish and fish products
food additives – food additives and flavourings
fruits, vegetables – fruits and vegetables
herbs, spices – herbs and spices
meat – meat and meat products (other than poultry)
nuts, seeds – nuts, nut products and seeds
other food product – other food product / mixed
poultry meat – poultry meat and poultry meat products
prepared dishes – prepared dishes and snacks
soups, broths – soups, broths, sauces and condiments
water (other) – water for human consumption (other)

distr. to m. countries – distribution to other member countries
distr. to non-m. countries – distribution to non-member countries
distr. to third countries – distribution to third countries
distribution (possible) – distribution on the market (possible)
distribution restricted – distribution restricted to notifying country
information not available – information on distribution not (yet) available
no distr. from n. country – no distribution from notifying country
no distr. to m. countries – no distribution to other member countries
no longer on market – product (presumably) no longer on the market
not placed on market – product not (yet) placed on the market
product - customs control – product under customs control
product - customs seals – product allowed to travel to destination under customs seals
product consumed – product already consumed
product past best date – product past best before date

FIGURE S47 Similarities between product category and distribution status within notifications on composition.

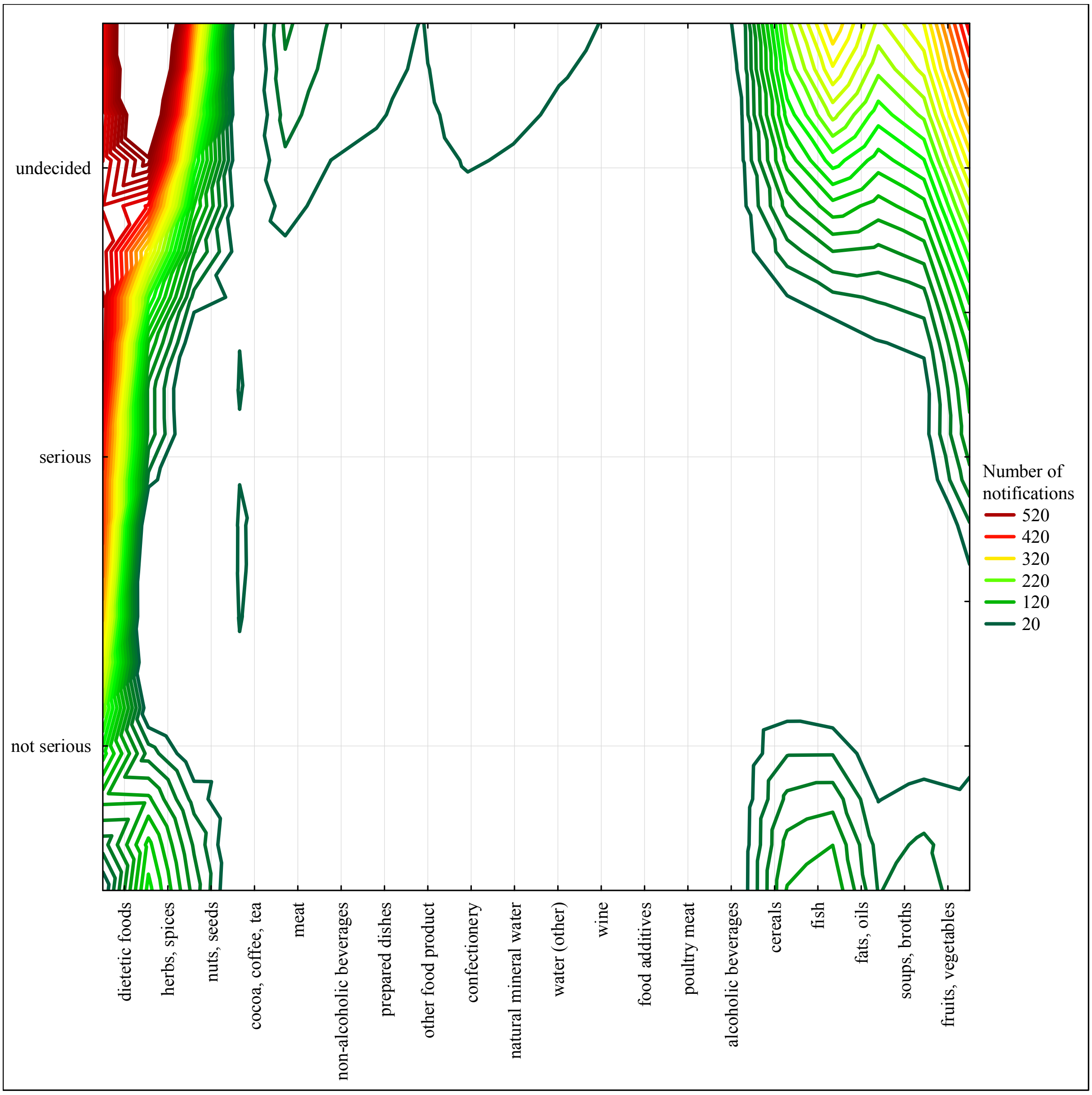

cereals – cereals and bakery products
cocoa, coffee, tea – cocoa and cocoa preparations, coffee and tea
dietetic foods – dietetic foods, food supplements, fortified foods
fats, oils – fats and oils
fish – fish and fish products
food additives – food additives and flavourings
fruits, vegetables – fruits and vegetables
herbs, spices – herbs and spices
meat – meat and meat products (other than poultry)
nuts, seeds – nuts, nut products and seeds
other food product – other food product / mixed
poultry meat – poultry meat and poultry meat products
prepared dishes – prepared dishes and snacks
soups, broths – soups, broths, sauces and condiments
water (other) – water for human consumption (other)

FIGURE S48 Similarities between product category and risk decision within notifications on composition.

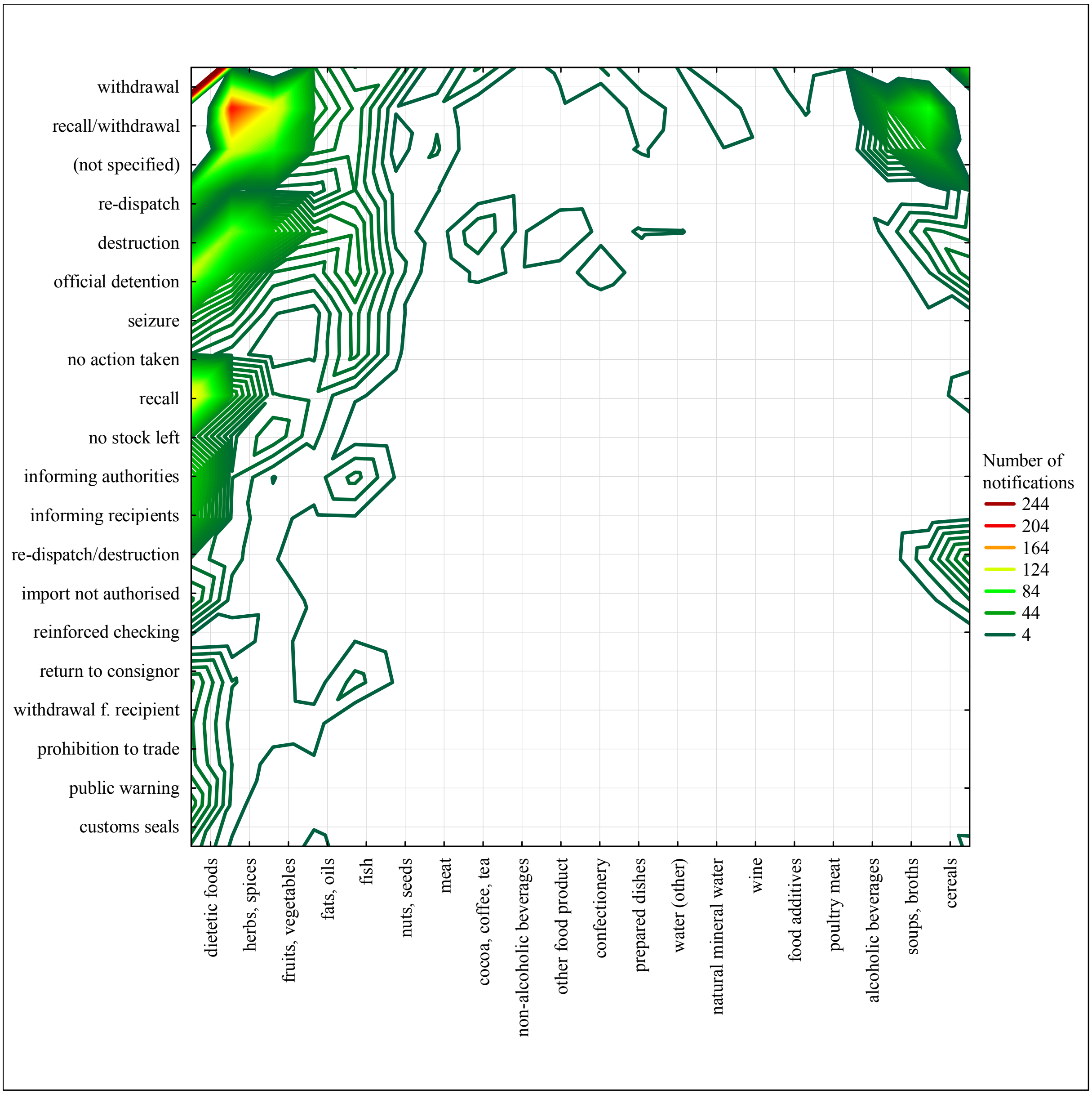

cereals – cereals and bakery products
cocoa, coffee, tea – cocoa and cocoa preparations, coffee and tea
dietetic foods – dietetic foods, food supplements, fortified foods
fats, oils – fats and oils
fish – fish and fish products
food additives – food additives and flavourings
fruits, vegetables – fruits and vegetables
herbs, spices – herbs and spices
meat – meat and meat products (other than poultry)
nuts, seeds – nuts, nut products and seeds
other food product – other food product / mixed
poultry meat – poultry meat and poultry meat products
prepared dishes – prepared dishes and snacks
soups, broths – soups, broths, sauces and condiments
water (other) – water for human consumption (other)

customs seals – placed under customs seals
prohibition to trade – prohibition to trade - sales ban
public warning – public warning - press release
recall – recall from consumers
recall/withdrawal – product recall or withdrawal
re-dispatch/destruction – re-dispatch or destruction
withdrawal – withdrawal from the market
withdrawal f. recipient – withdrawal from recipient(s)

FIGURE S49 Similarities between product category and action taken within notifications on composition.

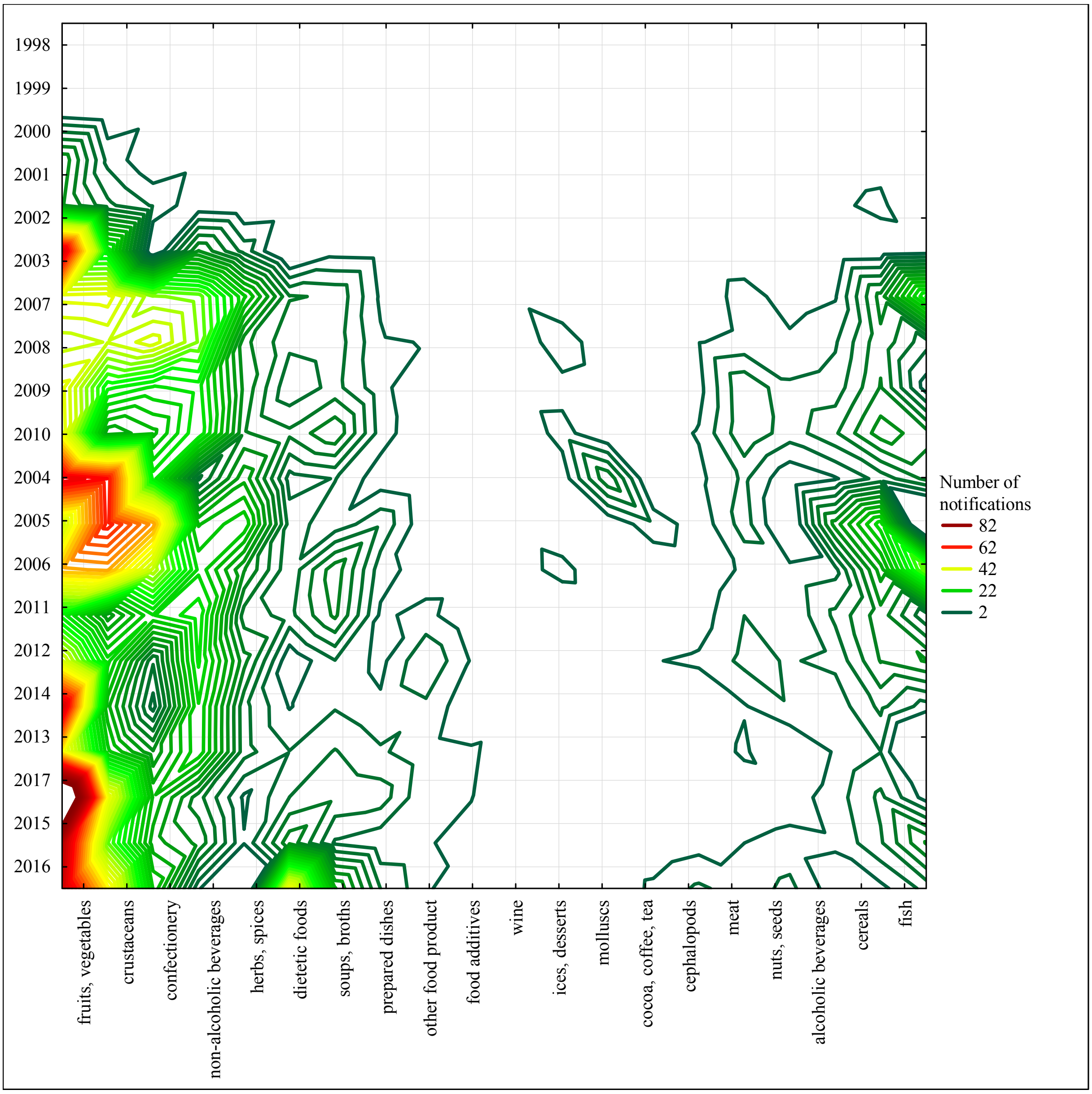

cephalopods – cephalopods and products thereof
cereals – cereals and bakery products
cocoa, coffee, tea – cocoa and cocoa preparations, coffee and tea
crustaceans – crustaceans and products thereof
dietetic foods – dietetic foods, food supplements, fortified foods
fish – fish and fish products
food additives – food additives and flavourings
fruits, vegetables – fruits and vegetables
herbs, spices – herbs and spices
ices, desserts – ices and desserts
meat – meat and meat products (other than poultry)
molluscs – molluscs and products thereof - (obsolete)
nuts, seeds – nuts, nut products and seeds
other food product – other food product / mixed
prepared dishes – prepared dishes and snacks
soups, broths – soups, broths, sauces and condiments

FIGURE S50 Similarities between product category and year within notifications on food additives and flavourings.

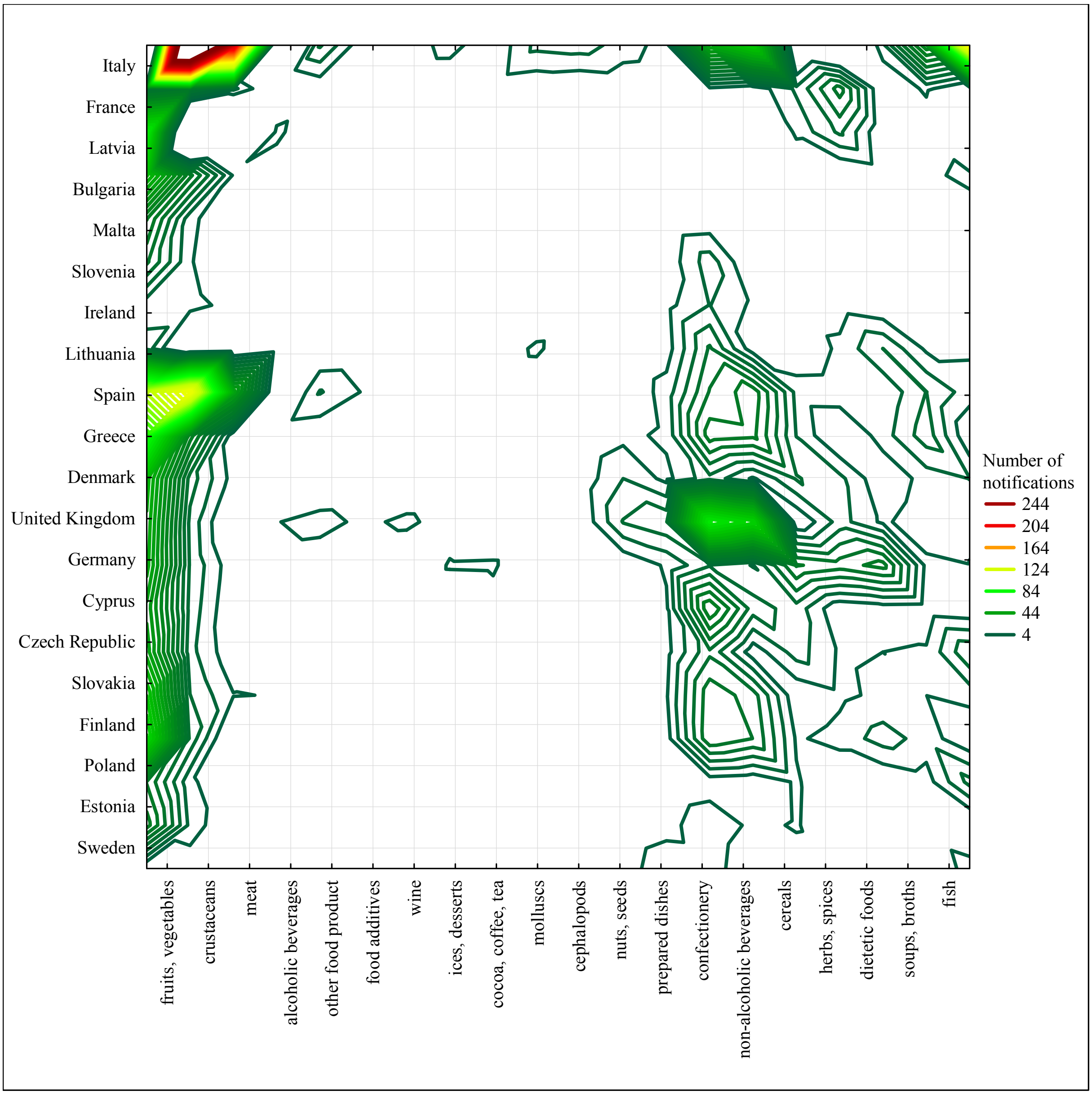

cephalopods – cephalopods and products thereof
cereals – cereals and bakery products
cocoa, coffee, tea – cocoa and cocoa preparations, coffee and tea
crustaceans – crustaceans and products thereof
dietetic foods – dietetic foods, food supplements, fortified foods
fish – fish and fish products
food additives – food additives and flavourings
fruits, vegetables – fruits and vegetables
herbs, spices – herbs and spices
ices, desserts – ices and desserts
meat – meat and meat products (other than poultry)
molluscs – molluscs and products thereof - (obsolete)
nuts, seeds – nuts, nut products and seeds
other food product – other food product / mixed
prepared dishes – prepared dishes and snacks
soups, broths – soups, broths, sauces and condiments

FIGURE S51 Similarities between product category and notifying country within notifications on food additives and flavourings.

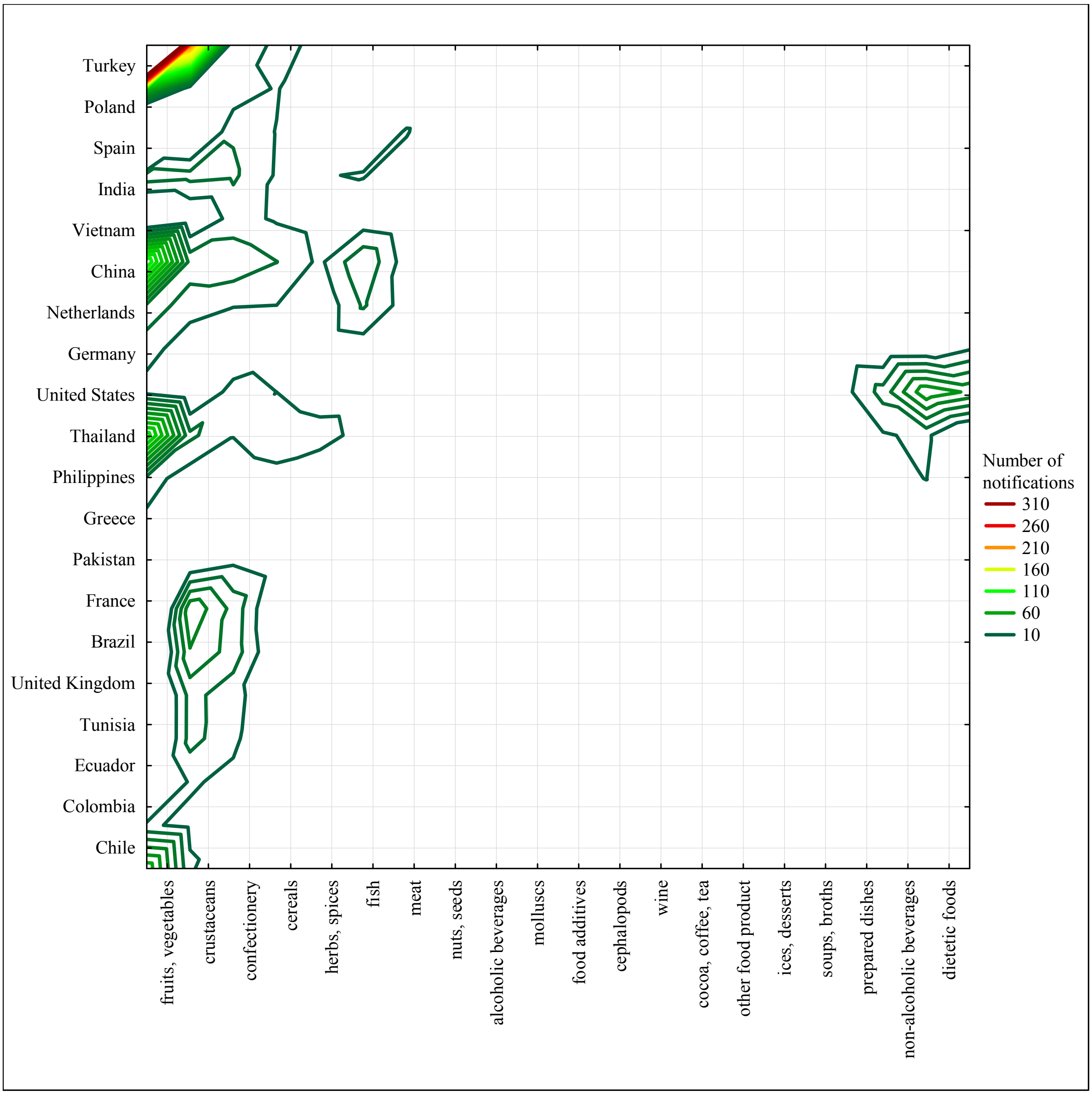

cephalopods – cephalopods and products thereof
cereals – cereals and bakery products
cocoa, coffee, tea – cocoa and cocoa preparations, coffee and tea
crustaceans – crustaceans and products thereof
dietetic foods – dietetic foods, food supplements, fortified foods
fish – fish and fish products
food additives – food additives and flavourings
fruits, vegetables – fruits and vegetables
herbs, spices – herbs and spices
ices, desserts – ices and desserts
meat – meat and meat products (other than poultry)
molluscs – molluscs and products thereof - (obsolete)
nuts, seeds – nuts, nut products and seeds
other food product – other food product / mixed
prepared dishes – prepared dishes and snacks
soups, broths – soups, broths, sauces and condiments

FIGURE S52 Similarities between product category and origin country within notifications on food additives and flavourings.

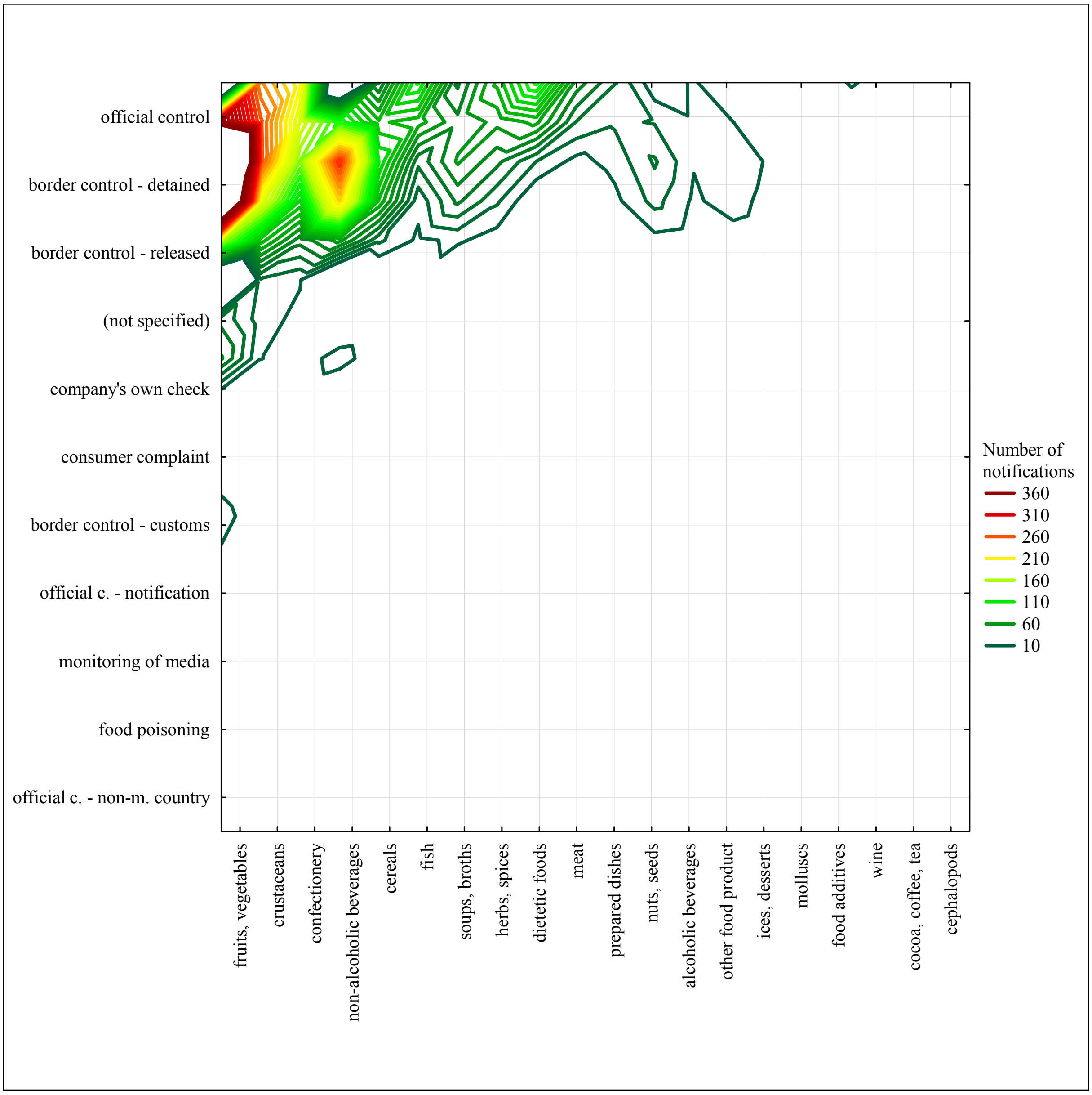

cephalopods – cephalopods and products thereof
cereals – cereals and bakery products
cocoa, coffee, tea – cocoa and cocoa preparations, coffee and tea
crustaceans – crustaceans and products thereof
dietetic foods – dietetic foods, food supplements, fortified foods
fish – fish and fish products
food additives – food additives and flavourings
fruits, vegetables – fruits and vegetables
herbs, spices – herbs and spices
ices, desserts – ices and desserts
meat – meat and meat products (other than poultry)
molluscs – molluscs and products thereof - (obsolete)
nuts, seeds – nuts, nut products and seeds
other food product – other food product / mixed
prepared dishes – prepared dishes and snacks
soups, broths – soups, broths, sauces and condiments

border control - detained – border control - consignment detained
border control - released – border control - consignment released
border control - customs – border control - consignment under customs
official c. - non-m. country – official control in non-member country
official c. - notification – official control following RASFF notification
official control – official control on the market

FIGURE S53 Similarities between product category and notification basis within notifications on food additives and flavourings.

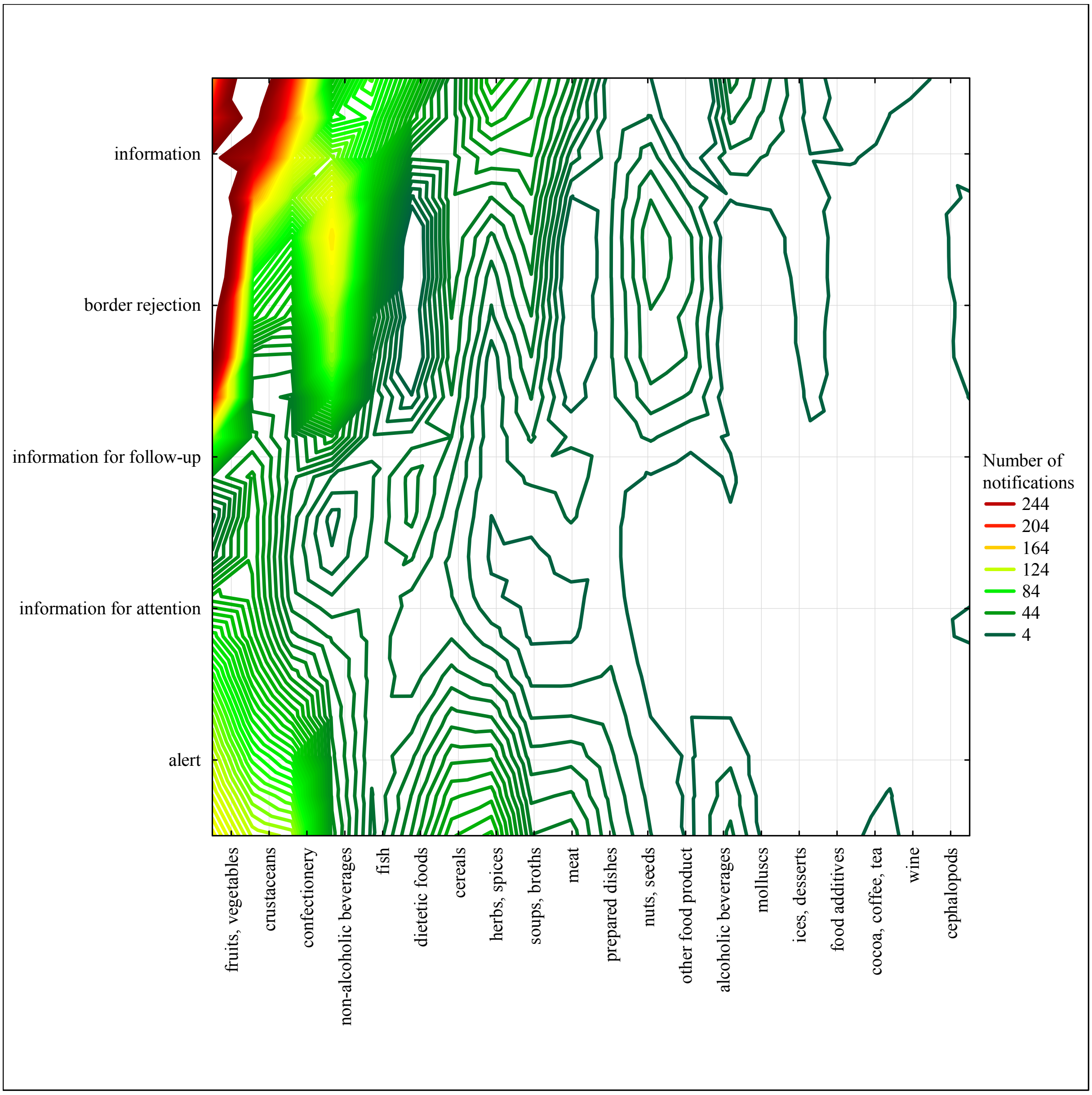

cephalopods – cephalopods and products thereof
cereals – cereals and bakery products
cocoa, coffee, tea – cocoa and cocoa preparations, coffee and tea
crustaceans – crustaceans and products thereof
dietetic foods – dietetic foods, food supplements, fortified foods
fish – fish and fish products
food additives – food additives and flavourings
fruits, vegetables – fruits and vegetables
herbs, spices – herbs and spices
ices, desserts – ices and desserts
meat – meat and meat products (other than poultry)
molluscs – molluscs and products thereof - (obsolete)
nuts, seeds – nuts, nut products and seeds
other food product – other food product / mixed
prepared dishes – prepared dishes and snacks
soups, broths – soups, broths, sauces and condiments

FIGURE S54 Similarities between product category and notification type within notifications on food additives and flavourings.

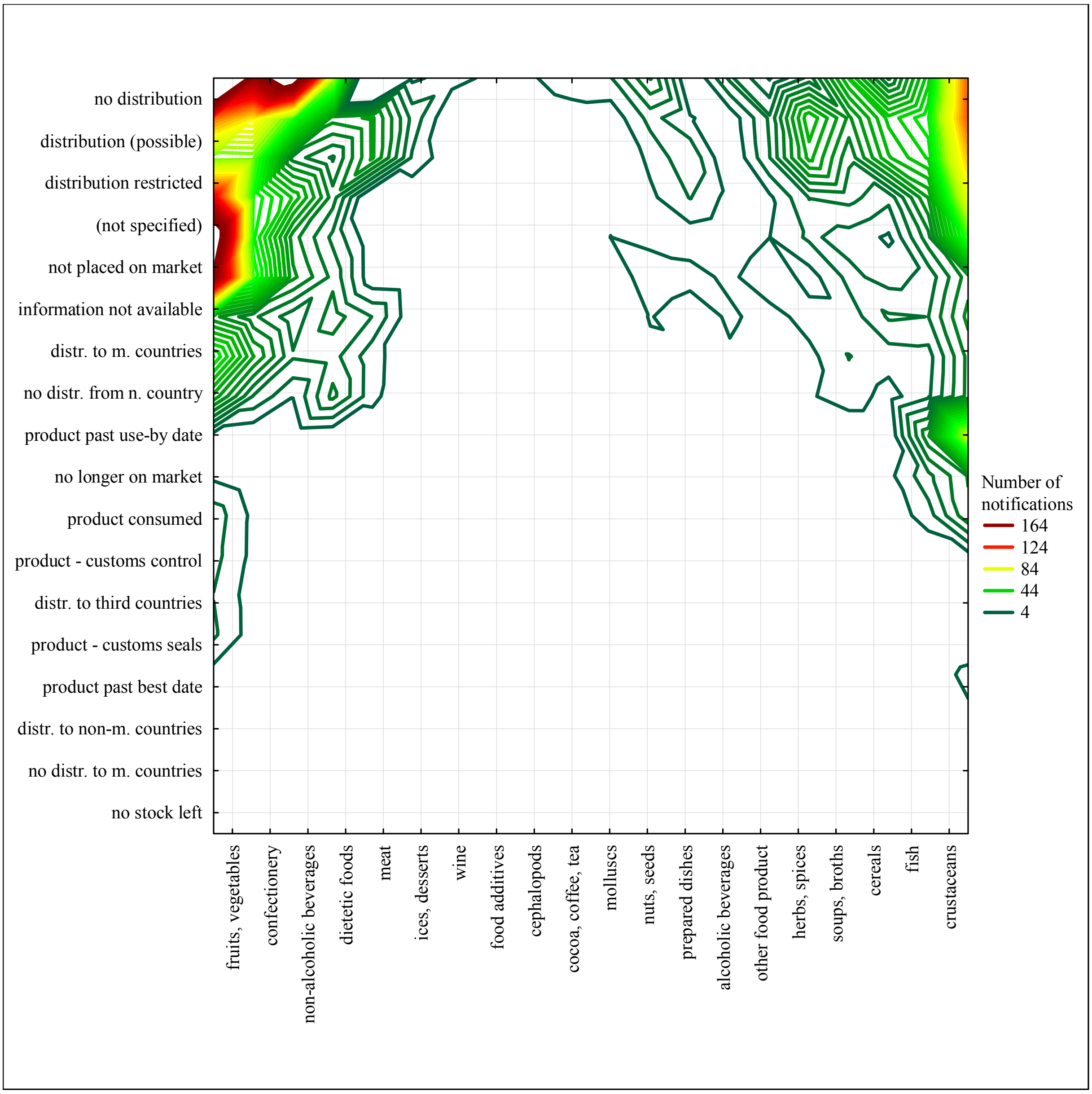

cephalopods – cephalopods and products thereof
cereals – cereals and bakery products
cocoa, coffee, tea – cocoa and cocoa preparations, coffee and tea
crustaceans – crustaceans and products thereof
dietetic foods – dietetic foods, food supplements, fortified foods
fish – fish and fish products
food additives – food additives and flavourings
fruits, vegetables – fruits and vegetables
herbs, spices – herbs and spices
ices, desserts – ices and desserts
meat – meat and meat products (other than poultry)
molluscs – molluscs and products thereof - (obsolete)
nuts, seeds – nuts, nut products and seeds
other food product – other food product / mixed
prepared dishes – prepared dishes and snacks
soups, broths – soups, broths, sauces and condiments

distr. to m. countries – distribution to other member countries
distr. to non-m. countries – distribution to non-member countries
distr. to third countries – distribution to third countries
distribution (possible) – distribution on the market (possible)
distribution restricted – distribution restricted to notifying country
information not available – information on distribution not (yet) available
no distr. from n. country – no distribution from notifying country
no distr. to m. countries – no distribution to other member countries
no longer on market – product (presumably) no longer on the market
not placed on market – product not (yet) placed on the market
product - customs control – product under customs control
product - customs seals – product allowed to travel to destination under customs seals
product consumed – product already consumed
product past best date – product past best before date

FIGURE S55 Similarities between product category and distribution status within notifications on food additives and flavourings.

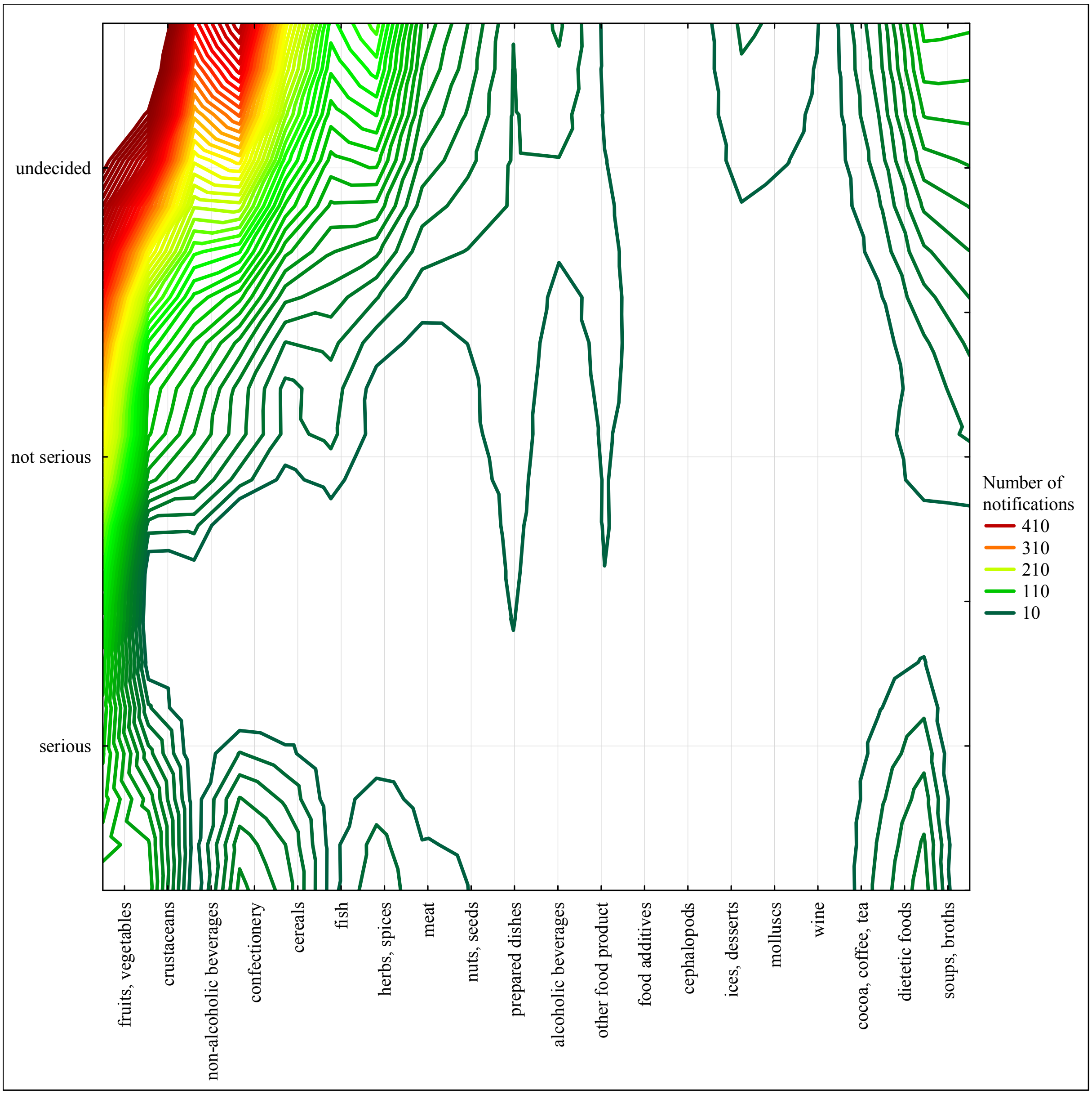

cephalopods – cephalopods and products thereof
cereals – cereals and bakery products
cocoa, coffee, tea – cocoa and cocoa preparations, coffee and tea
crustaceans – crustaceans and products thereof
dietetic foods – dietetic foods, food supplements, fortified foods
fish – fish and fish products
food additives – food additives and flavourings
fruits, vegetables – fruits and vegetables
herbs, spices – herbs and spices
ices, desserts – ices and desserts
meat – meat and meat products (other than poultry)
molluscs – molluscs and products thereof - (obsolete)
nuts, seeds – nuts, nut products and seeds
other food product – other food product / mixed
prepared dishes – prepared dishes and snacks
soups, broths – soups, broths, sauces and condiments

FIGURE S56 Similarities between product category and risk decision within notifications on food additives and flavourings.

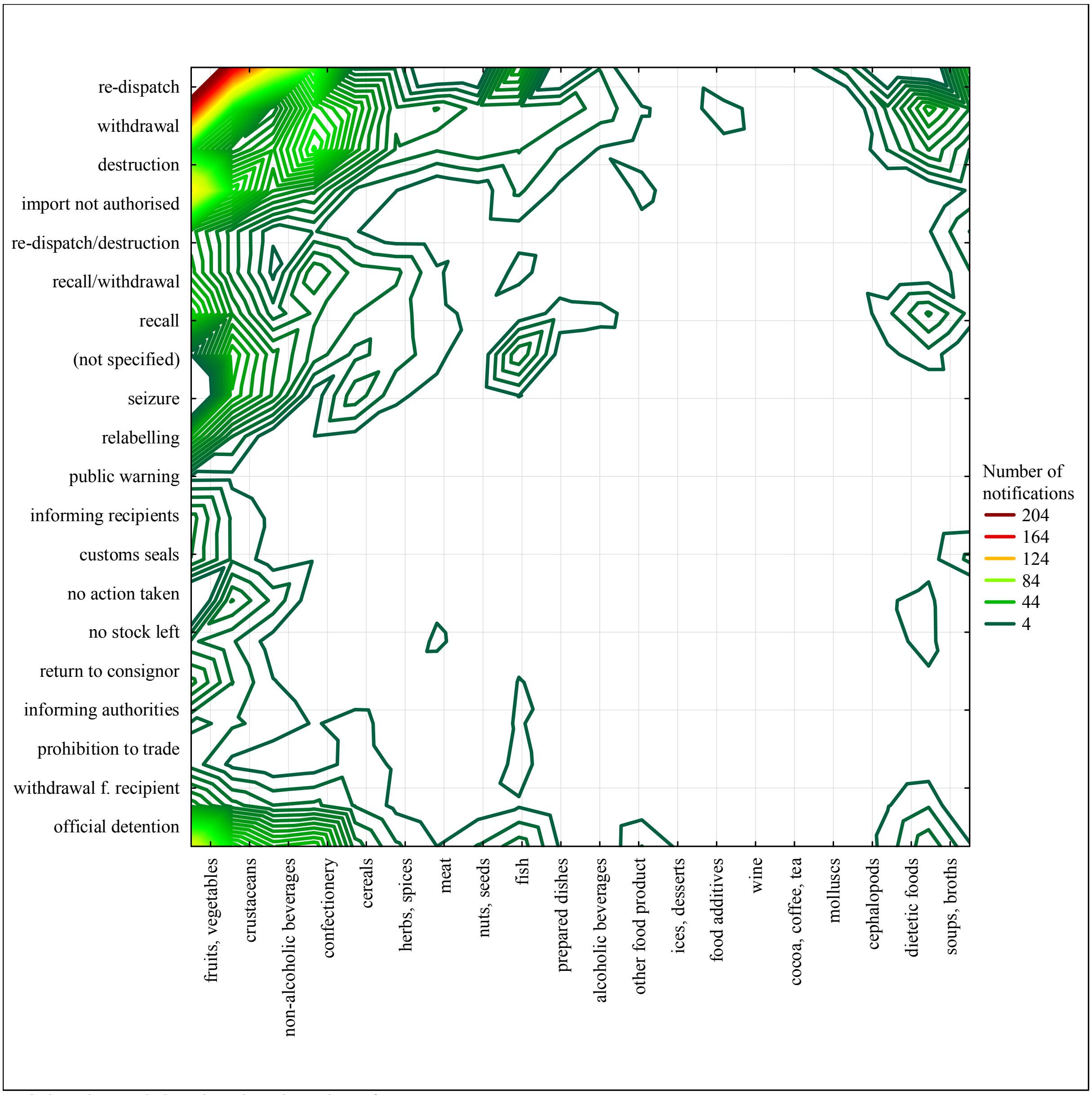

cephalopods – cephalopods and products thereof
cereals – cereals and bakery products
cocoa, coffee, tea – cocoa and cocoa preparations, coffee and tea
crustaceans – crustaceans and products thereof
dietetic foods – dietetic foods, food supplements, fortified foods
fish – fish and fish products
food additives – food additives and flavourings
fruits, vegetables – fruits and vegetables
herbs, spices – herbs and spices
ices, desserts – ices and desserts
meat – meat and meat products (other than poultry)
molluscs – molluscs and products thereof - (obsolete)
nuts, seeds – nuts, nut products and seeds
other food product – other food product / mixed
prepared dishes – prepared dishes and snacks
soups, broths – soups, broths, sauces and condiments

customs seals – placed under customs seals
prohibition to trade – prohibition to trade - sales ban
public warning – public warning - press release
recall – recall from consumers
recall/withdrawal – product recall or withdrawal
re-dispatch/destruction – re-dispatch or destruction
withdrawal – withdrawal from the market
withdrawal f. recipient – withdrawal from recipient(s)

FIGURE S57 Similarities between product category and action taken within notifications on food additives and flavourings.

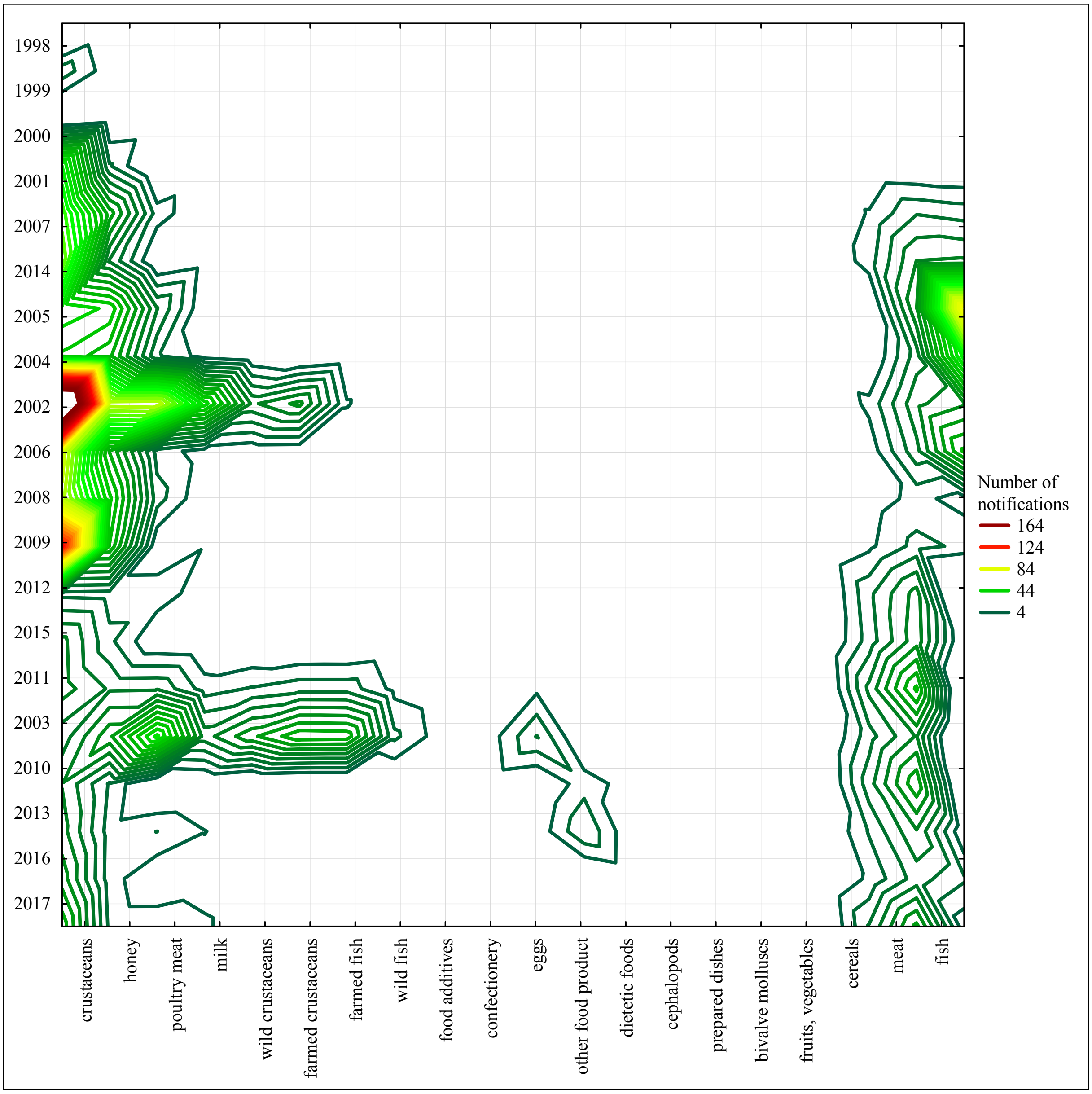

bivalve molluscs – bivalve molluscs and products thereof
cephalopods – cephalopods and products thereof
cereals – cereals and bakery products
crustaceans – crustaceans and products thereof
dietetic foods – dietetic foods, food supplements, fortified foods
eggs – eggs and egg products
farmed crustaceans – farmed crustaceans and products thereof - (obsolete)
farmed fish – farmed fish and products thereof (other than crustaceans and molluscs) - (obsolete)
fish – fish and fish products
food additives – food additives and flavourings
fruits, vegetables – fruits and vegetables
honey – honey and royal jelly
meat – meat and meat products (other than poultry)
milk – milk and milk products
other food product – other food product / mixed
poultry meat – poultry meat and poultry meat products
prepared dishes – prepared dishes and snacks
wild fish – wild caught fish and products thereof (other than crustaceans and molluscs) - (obsolete)
wild crustaceans – wild caught crustaceans and products thereof - (obsolete)

FIGURE S58 Similarities between product category and year within notifications on residues of veterinary medicinal products.

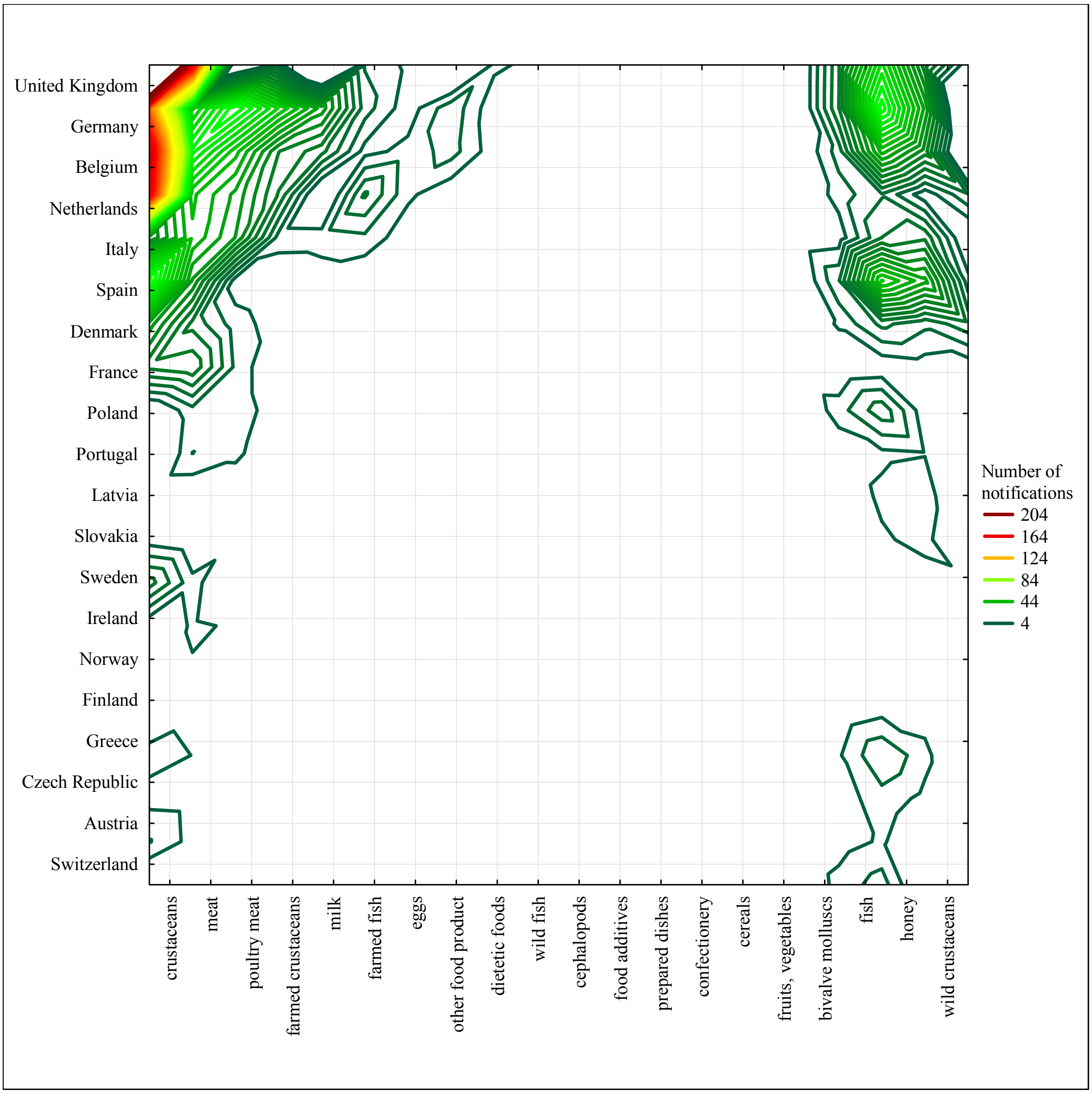

bivalve molluscs – bivalve molluscs and products thereof
cephalopods – cephalopods and products thereof
cereals – cereals and bakery products
crustaceans – crustaceans and products thereof
dietetic foods – dietetic foods, food supplements, fortified foods
eggs – eggs and egg products
farmed crustaceans – farmed crustaceans and products thereof - (obsolete)
farmed fish – farmed fish and products thereof (other than crustaceans and molluscs) - (obsolete)
fish – fish and fish products
food additives – food additives and flavourings
fruits, vegetables – fruits and vegetables
honey – honey and royal jelly
meat – meat and meat products (other than poultry)
milk – milk and milk products
other food product – other food product / mixed
poultry meat – poultry meat and poultry meat products
prepared dishes – prepared dishes and snacks
wild fish – wild caught fish and products thereof (other than crustaceans and molluscs) - (obsolete)
wild crustaceans – wild caught crustaceans and products thereof - (obsolete)

FIGURE S59 Similarities between product category and notifying country within notifications on residues of veterinary medicinal products.

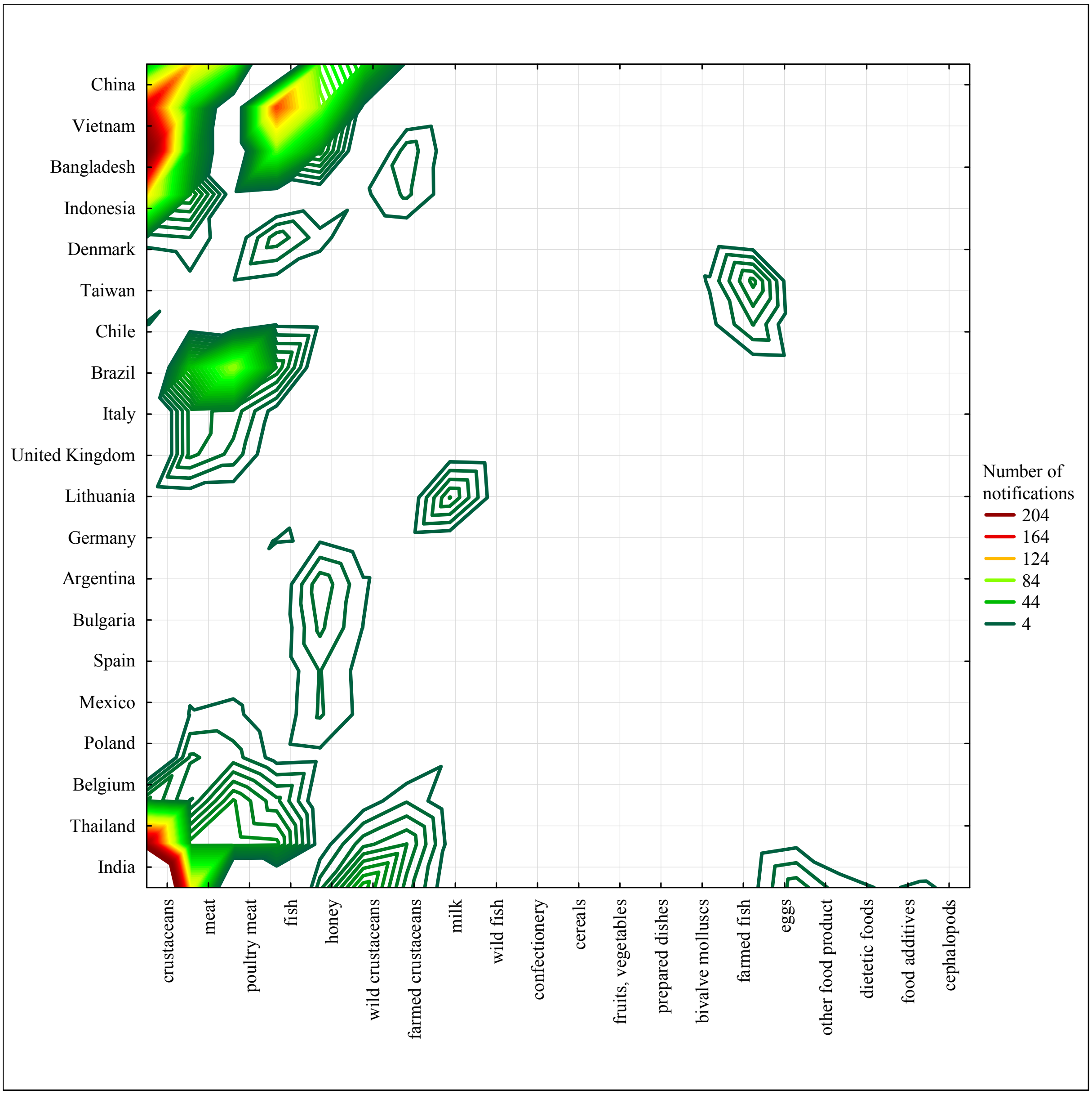

bivalve molluscs – bivalve molluscs and products thereof
cephalopods – cephalopods and products thereof
cereals – cereals and bakery products
crustaceans – crustaceans and products thereof
dietetic foods – dietetic foods, food supplements, fortified foods
eggs – eggs and egg products
farmed crustaceans – farmed crustaceans and products thereof - (obsolete)
farmed fish – farmed fish and products thereof (other than crustaceans and molluscs) - (obsolete)
fish – fish and fish products
food additives – food additives and flavourings
fruits, vegetables – fruits and vegetables
honey – honey and royal jelly
meat – meat and meat products (other than poultry)
milk – milk and milk products
other food product – other food product / mixed
poultry meat – poultry meat and poultry meat products
prepared dishes – prepared dishes and snacks
wild fish – wild caught fish and products thereof (other than crustaceans and molluscs) - (obsolete)
wild crustaceans – wild caught crustaceans and products thereof - (obsolete)

FIGURE S60 Similarities between product category and origin country within notifications on residues of veterinary medicinal products.

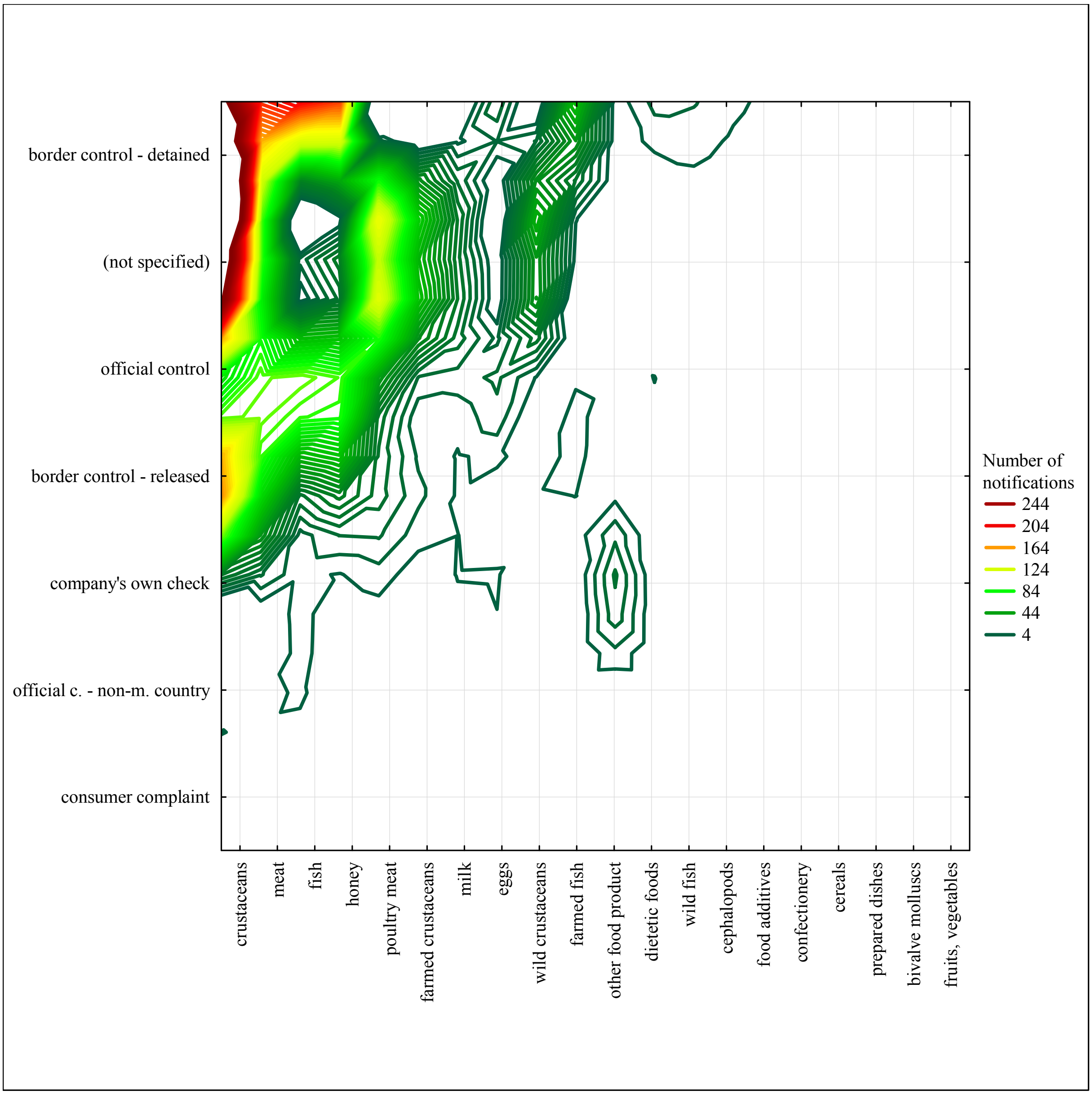

bivalve molluscs – bivalve molluscs and products thereof
cephalopods – cephalopods and products thereof
cereals – cereals and bakery products
crustaceans – crustaceans and products thereof
dietetic foods – dietetic foods, food supplements, fortified foods
eggs – eggs and egg products
farmed crustaceans – farmed crustaceans and products thereof - (obsolete)
farmed fish – farmed fish and products thereof (other than crustaceans and molluscs) - (obsolete)
fish – fish and fish products
food additives – food additives and flavourings
fruits, vegetables – fruits and vegetables
honey – honey and royal jelly
meat – meat and meat products (other than poultry)
milk – milk and milk products
other food product – other food product / mixed
poultry meat – poultry meat and poultry meat products
prepared dishes – prepared dishes and snacks
wild fish – wild caught fish and products thereof (other than crustaceans and molluscs) - (obsolete)
wild crustaceans – wild caught crustaceans and products thereof - (obsolete)

border control - detained – border control - consignment detained
border control - released – border control - consignment released
official c. - non-m. country – official control in non-member country
official control – official control on the market

FIGURE S61 Similarities between product category and notification basis within notifications on residues of veterinary medicinal products.

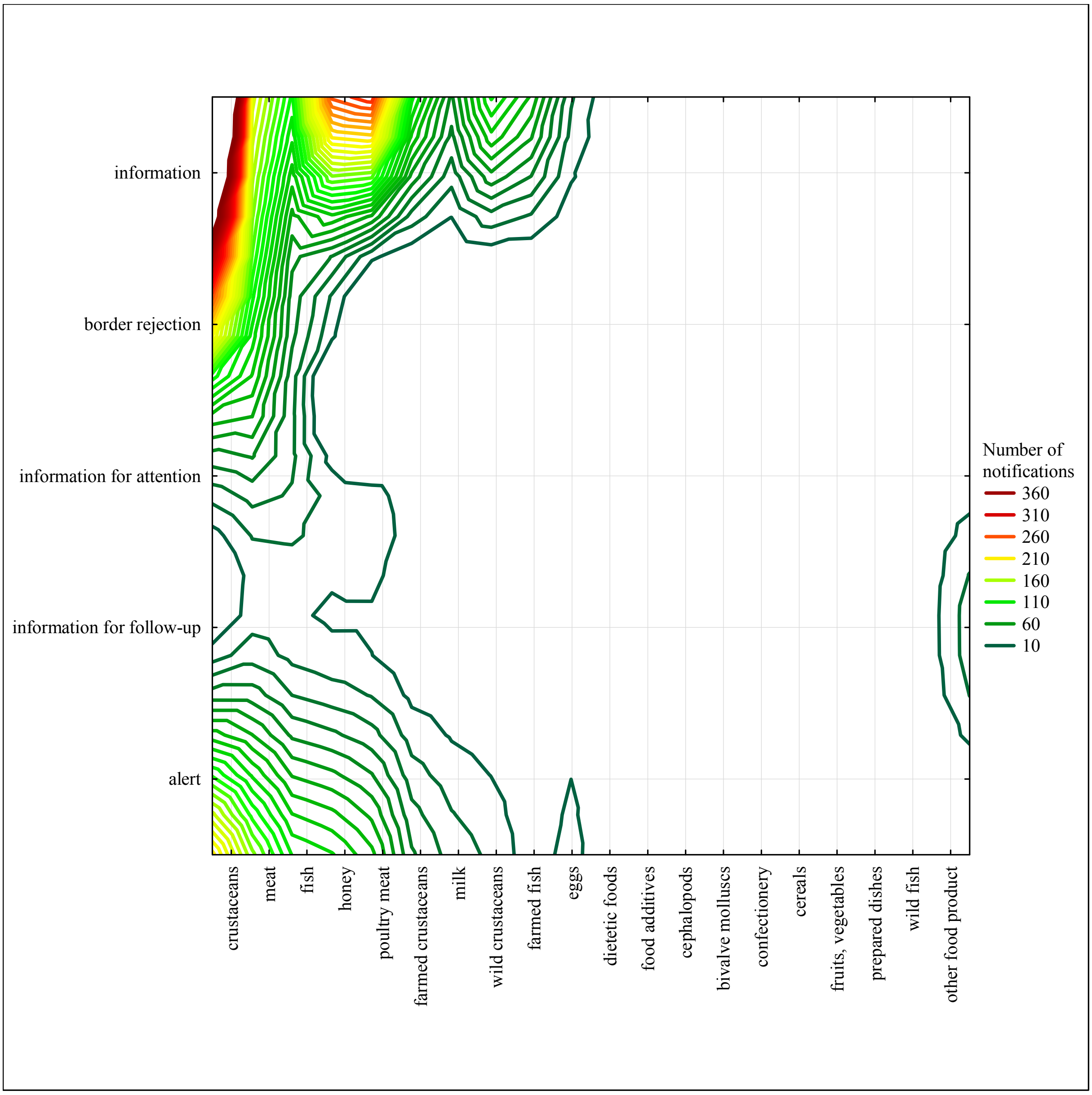

bivalve molluscs – bivalve molluscs and products thereof
cephalopods – cephalopods and products thereof
cereals – cereals and bakery products
crustaceans – crustaceans and products thereof
dietetic foods – dietetic foods, food supplements, fortified foods
eggs – eggs and egg products
farmed crustaceans – farmed crustaceans and products thereof - (obsolete)
farmed fish – farmed fish and products thereof (other than crustaceans and molluscs) - (obsolete)
fish – fish and fish products
food additives – food additives and flavourings
fruits, vegetables – fruits and vegetables
honey – honey and royal jelly
meat – meat and meat products (other than poultry)
milk – milk and milk products
other food product – other food product / mixed
poultry meat – poultry meat and poultry meat products
prepared dishes – prepared dishes and snacks
wild fish – wild caught fish and products thereof (other than crustaceans and molluscs) - (obsolete)
wild crustaceans – wild caught crustaceans and products thereof - (obsolete)

FIGURE S62 Similarities between product category and notification type within notifications on residues of veterinary medicinal products.

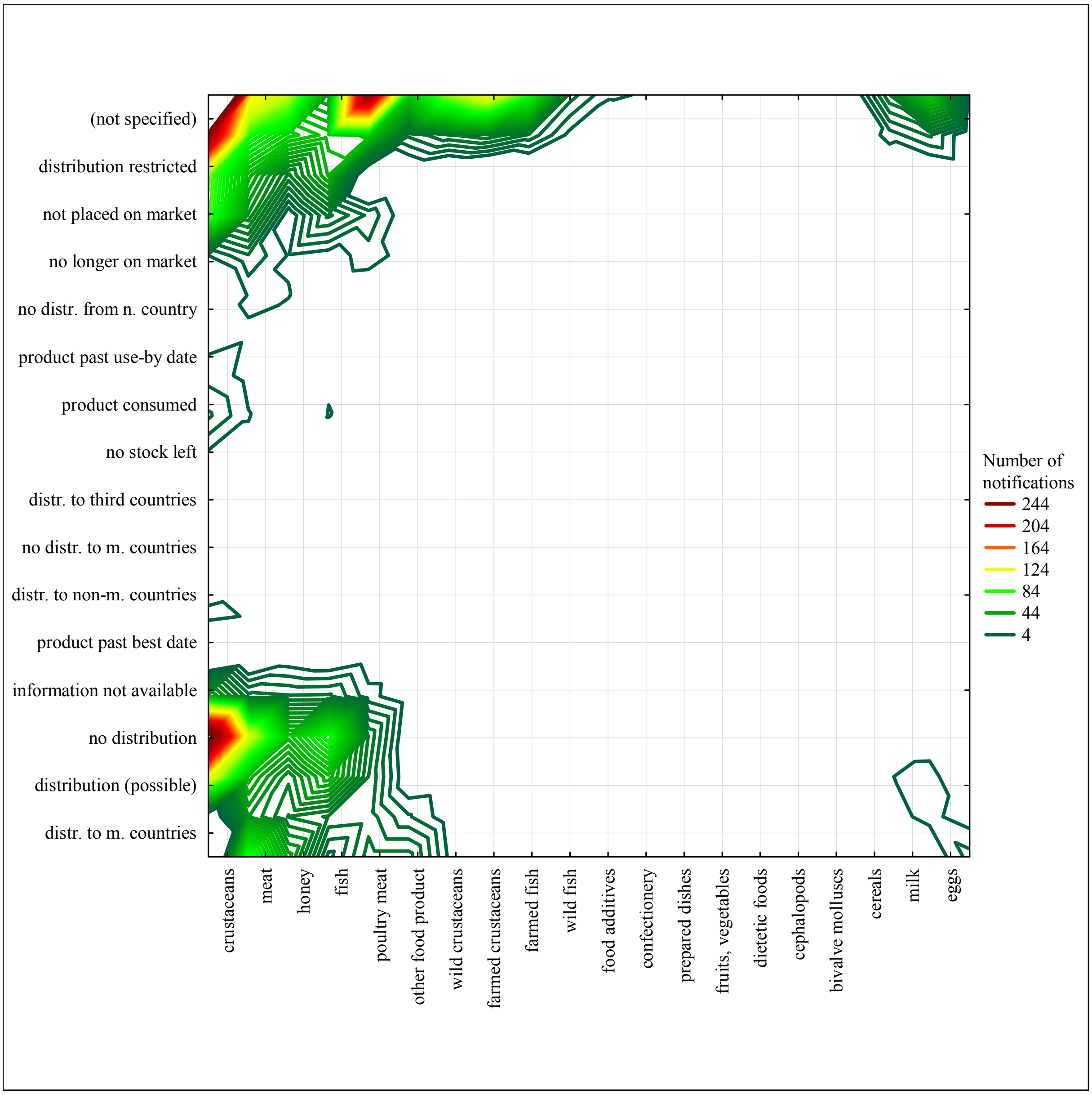

bivalve molluscs – bivalve molluscs and products thereof
cephalopods – cephalopods and products thereof
cereals – cereals and bakery products
crustaceans – crustaceans and products thereof
dietetic foods – dietetic foods, food supplements, fortified foods
eggs – eggs and egg products
farmed crustaceans – farmed crustaceans and products thereof - (obsolete)
farmed fish – farmed fish and products thereof (other than crustaceans and molluscs) - (obsolete)
fish – fish and fish products
food additives – food additives and flavourings
fruits, vegetables – fruits and vegetables
honey – honey and royal jelly
meat – meat and meat products (other than poultry)
milk – milk and milk products
other food product – other food product / mixed
poultry meat – poultry meat and poultry meat products
prepared dishes – prepared dishes and snacks
wild fish – wild caught fish and products thereof (other than crustaceans and molluscs) - (obsolete)
wild crustaceans – wild caught crustaceans and products thereof - (obsolete)

distr. to m. countries – distribution to other member countries
distr. to non-m. countries – distribution to non-member countries
distr. to third countries – distribution to third countries
distribution (possible) – distribution on the market (possible)
distribution restricted – distribution restricted to notifying country
information not available – information on distribution not (yet) available
no distr. from n. country – no distribution from notifying country
no distr. to m. countries – no distribution to other member countries
no longer on market – product (presumably) no longer on the market
not placed on market – product not (yet) placed on the market
product consumed – product already consumed

FIGURE S63 Similarities between product category and distribution status within notifications on residues of veterinary medicinal products.

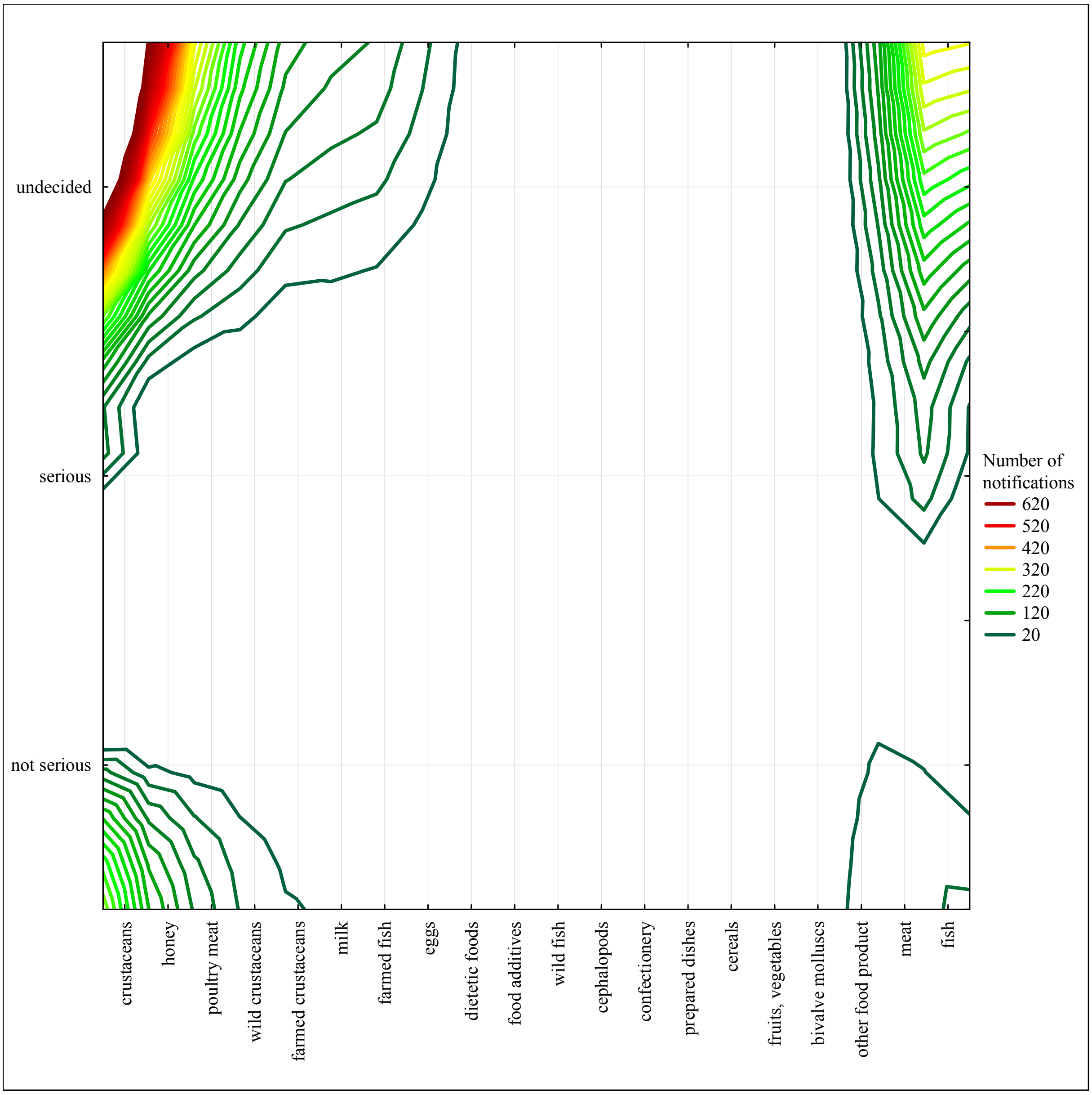

bivalve molluscs – bivalve molluscs and products thereof
cephalopods – cephalopods and products thereof
cereals – cereals and bakery products
crustaceans – crustaceans and products thereof
dietetic foods – dietetic foods, food supplements, fortified foods
eggs – eggs and egg products
farmed crustaceans – farmed crustaceans and products thereof - (obsolete)
farmed fish – farmed fish and products thereof (other than crustaceans and molluscs) - (obsolete)
fish – fish and fish products
food additives – food additives and flavourings
fruits, vegetables – fruits and vegetables
honey – honey and royal jelly
meat – meat and meat products (other than poultry)
milk – milk and milk products
other food product – other food product / mixed
poultry meat – poultry meat and poultry meat products
prepared dishes – prepared dishes and snacks
wild fish – wild caught fish and products thereof (other than crustaceans and molluscs) - (obsolete)
wild crustaceans – wild caught crustaceans and products thereof - (obsolete)

FIGURE S64 Similarities between product category and risk decision within notifications on residues of veterinary medicinal products.

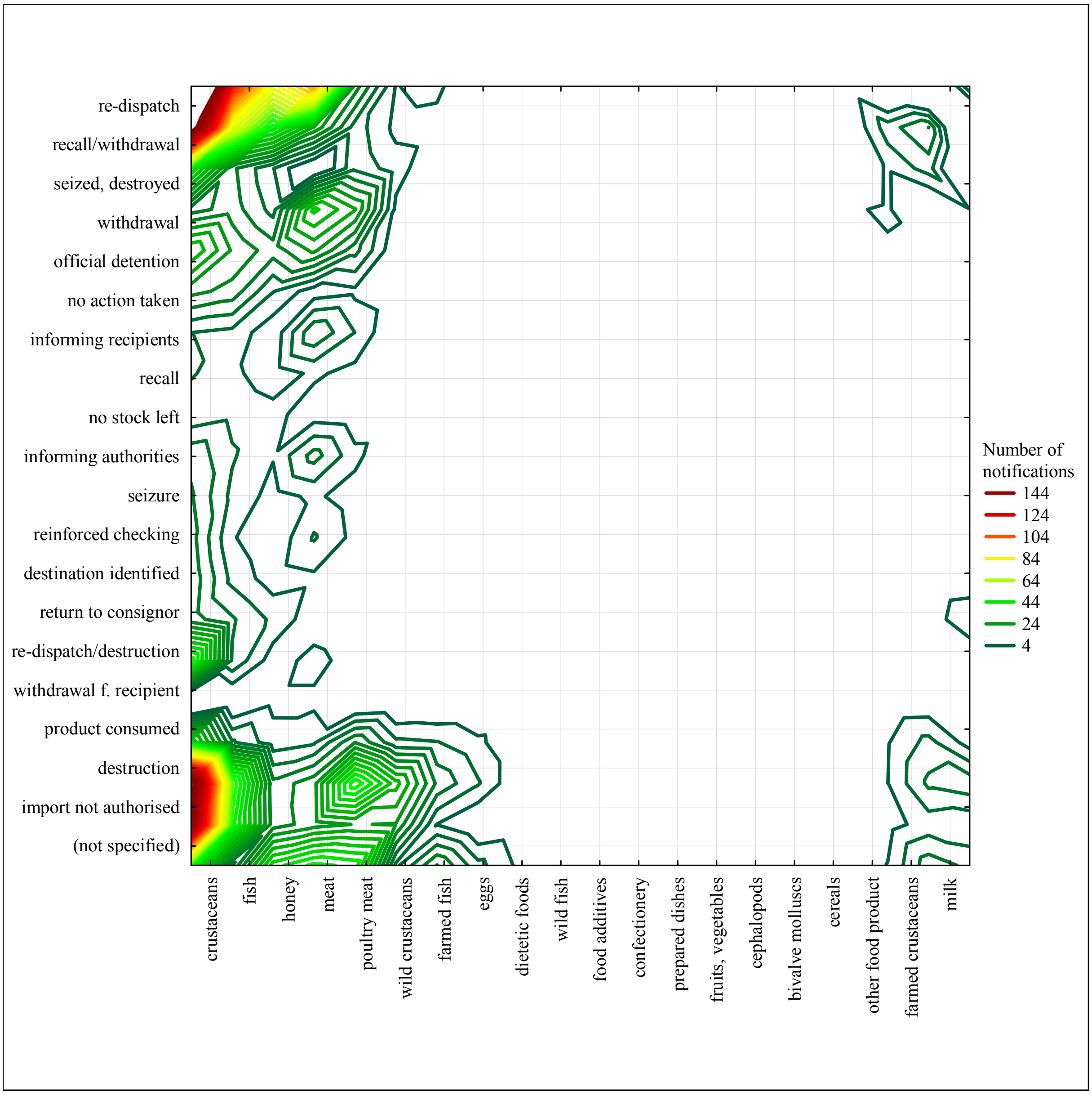

bivalve molluscs – bivalve molluscs and products thereof
cephalopods – cephalopods and products thereof
cereals – cereals and bakery products
crustaceans – crustaceans and products thereof
dietetic foods – dietetic foods, food supplements, fortified foods
eggs – eggs and egg products
farmed crustaceans – farmed crustaceans and products thereof - (obsolete)
farmed fish – farmed fish and products thereof (other than crustaceans and molluscs) - (obsolete)
fish – fish and fish products
food additives – food additives and flavourings
fruits, vegetables – fruits and vegetables
honey – honey and royal jelly
meat – meat and meat products (other than poultry)
milk – milk and milk products
other food product – other food product / mixed
poultry meat – poultry meat and poultry meat products
prepared dishes – prepared dishes and snacks
wild fish – wild caught fish and products thereof (other than crustaceans and molluscs) - (obsolete)
wild crustaceans – wild caught crustaceans and products thereof - (obsolete)

destination identified – destination of the product identified
product consumed – product already consumed
recall/withdrawal – product recall or withdrawal
re-dispatch/destruction – re-dispatch or destruction
seized, destroyed – product seized and will be destroyed
withdrawal – withdrawal from the market
withdrawal f. recipient – withdrawal from recipient(s)

FIGURE S65 Similarities between product category and action taken within notifications on residues of veterinary medicinal products.

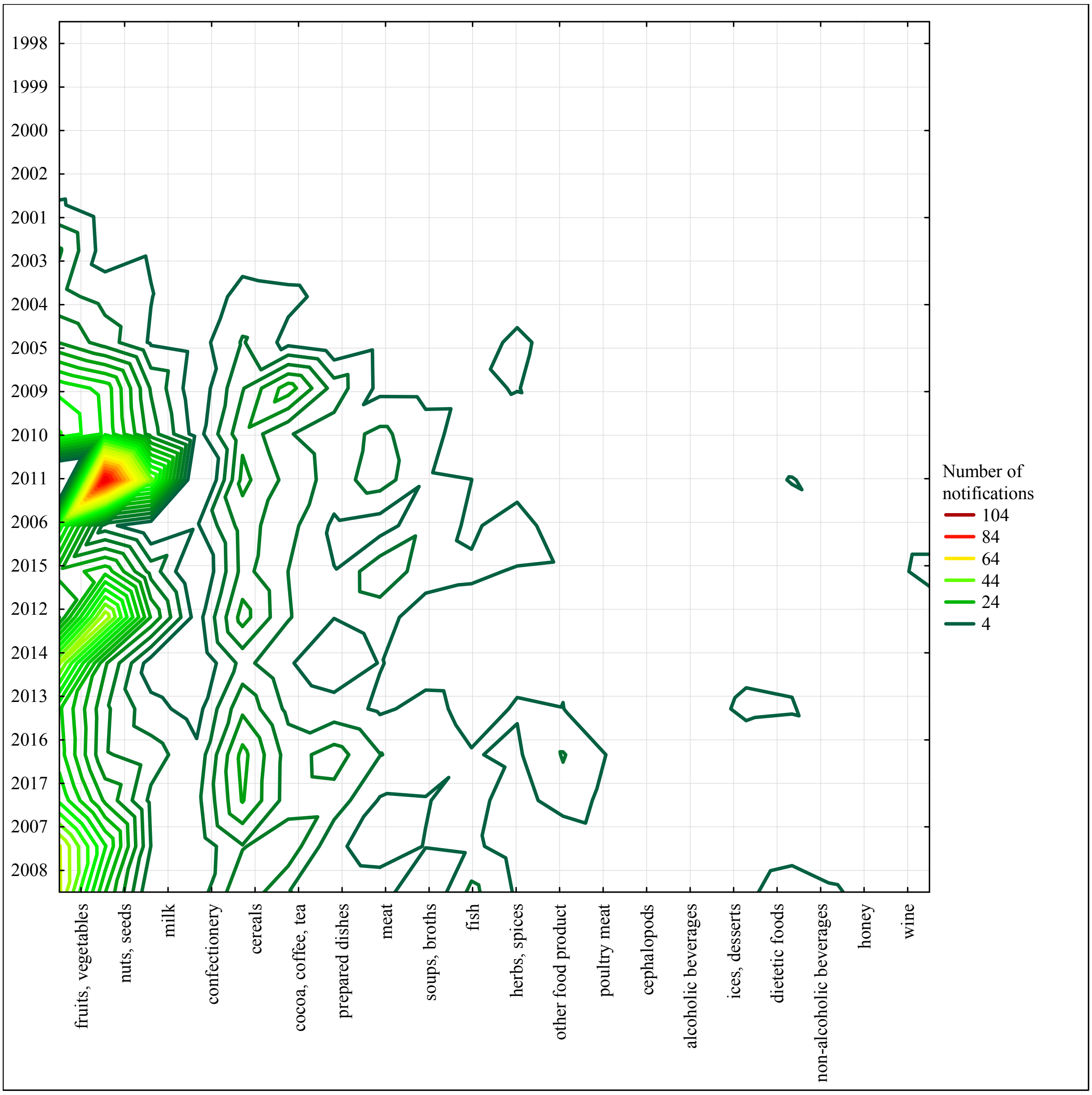

cephalopods – cephalopods and products thereof
cereals – cereals and bakery products
cocoa, coffee, tea – cocoa and cocoa preparations, coffee and tea
dietetic foods – dietetic foods, food supplements, fortified foods
fish – fish and fish products
fruits, vegetables – fruits and vegetables
herbs, spices – herbs and spices
honey – honey and royal jelly
ices, desserts – ices and desserts
meat – meat and meat products (other than poultry)
milk – milk and milk products
nuts, seeds – nuts, nut products and seeds
other food product – other food product / mixed
poultry meat – poultry meat and poultry meat products
prepared dishes – prepared dishes and snacks
soups, broths – soups, broths, sauces and condiments

FIGURE S66 Similarities between product category and year within notifications on foreign bodies.

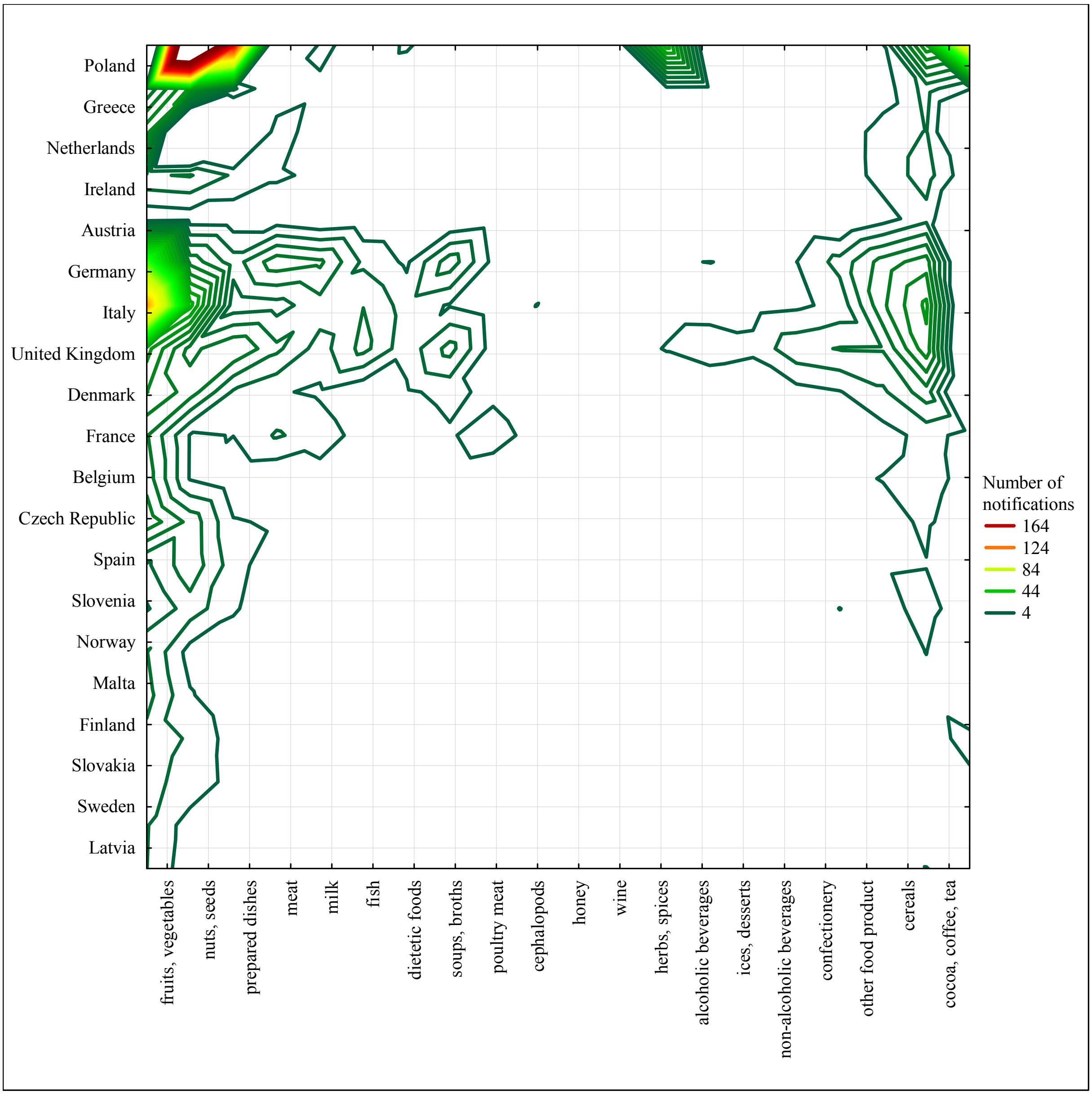

cephalopods – cephalopods and products thereof
cereals – cereals and bakery products
cocoa, coffee, tea – cocoa and cocoa preparations, coffee and tea
dietetic foods – dietetic foods, food supplements, fortified foods
fish – fish and fish products
fruits, vegetables – fruits and vegetables
herbs, spices – herbs and spices
honey – honey and royal jelly
ices, desserts – ices and desserts
meat – meat and meat products (other than poultry)
milk – milk and milk products
nuts, seeds – nuts, nut products and seeds
other food product – other food product / mixed
poultry meat – poultry meat and poultry meat products
prepared dishes – prepared dishes and snacks
soups, broths – soups, broths, sauces and condiments

FIGURE S67 Similarities between product category and notifying country within notifications on foreign bodies.

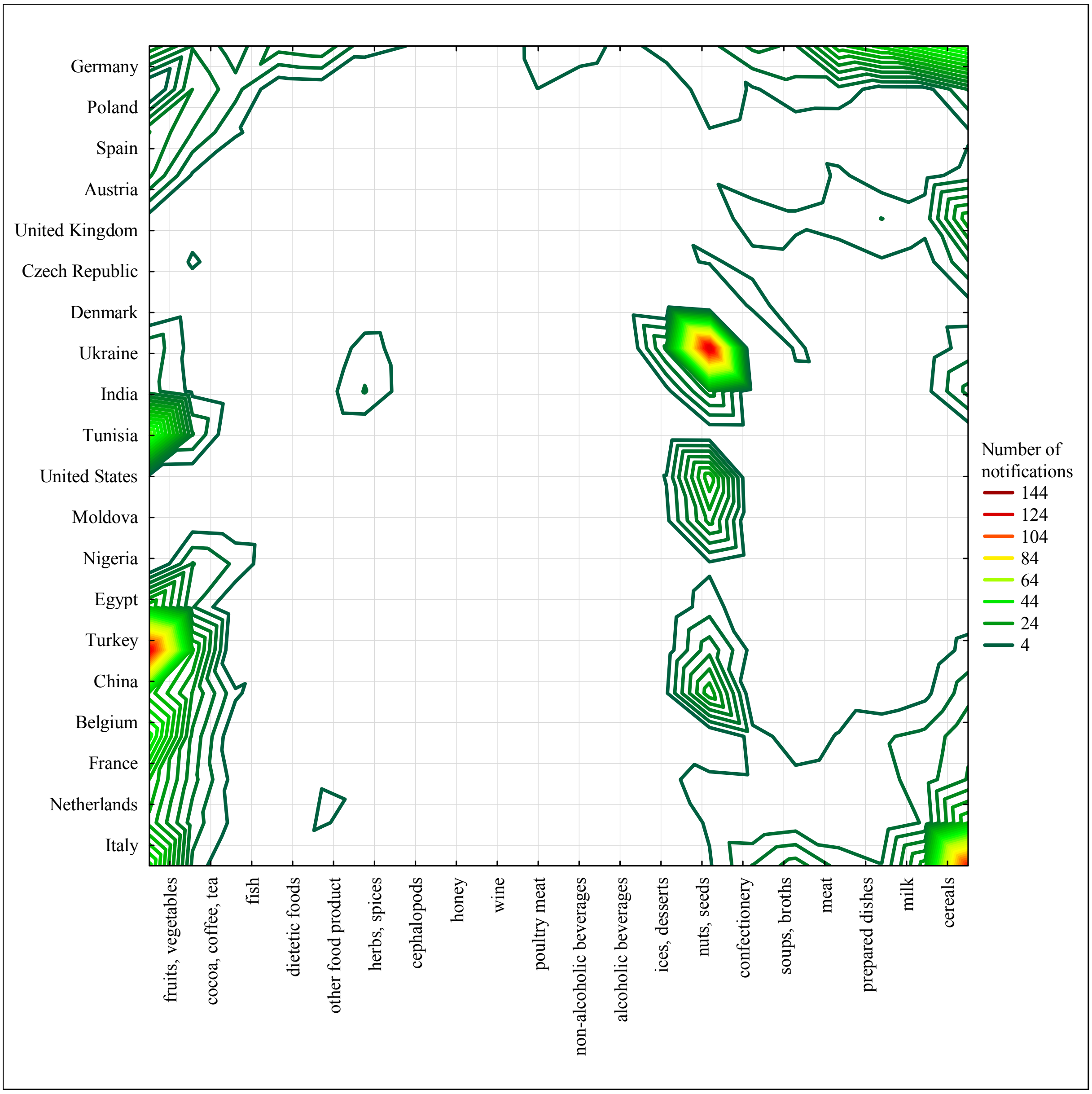

cephalopods – cephalopods and products thereof
cereals – cereals and bakery products
cocoa, coffee, tea – cocoa and cocoa preparations, coffee and tea
dietetic foods – dietetic foods, food supplements, fortified foods
fish – fish and fish products
fruits, vegetables – fruits and vegetables
herbs, spices – herbs and spices
honey – honey and royal jelly
ices, desserts – ices and desserts
meat – meat and meat products (other than poultry)
milk – milk and milk products
nuts, seeds – nuts, nut products and seeds
other food product – other food product / mixed
poultry meat – poultry meat and poultry meat products
prepared dishes – prepared dishes and snacks
soups, broths – soups, broths, sauces and condiments

FIGURE S68 Similarities between product category and origin country within notifications on foreign bodies.

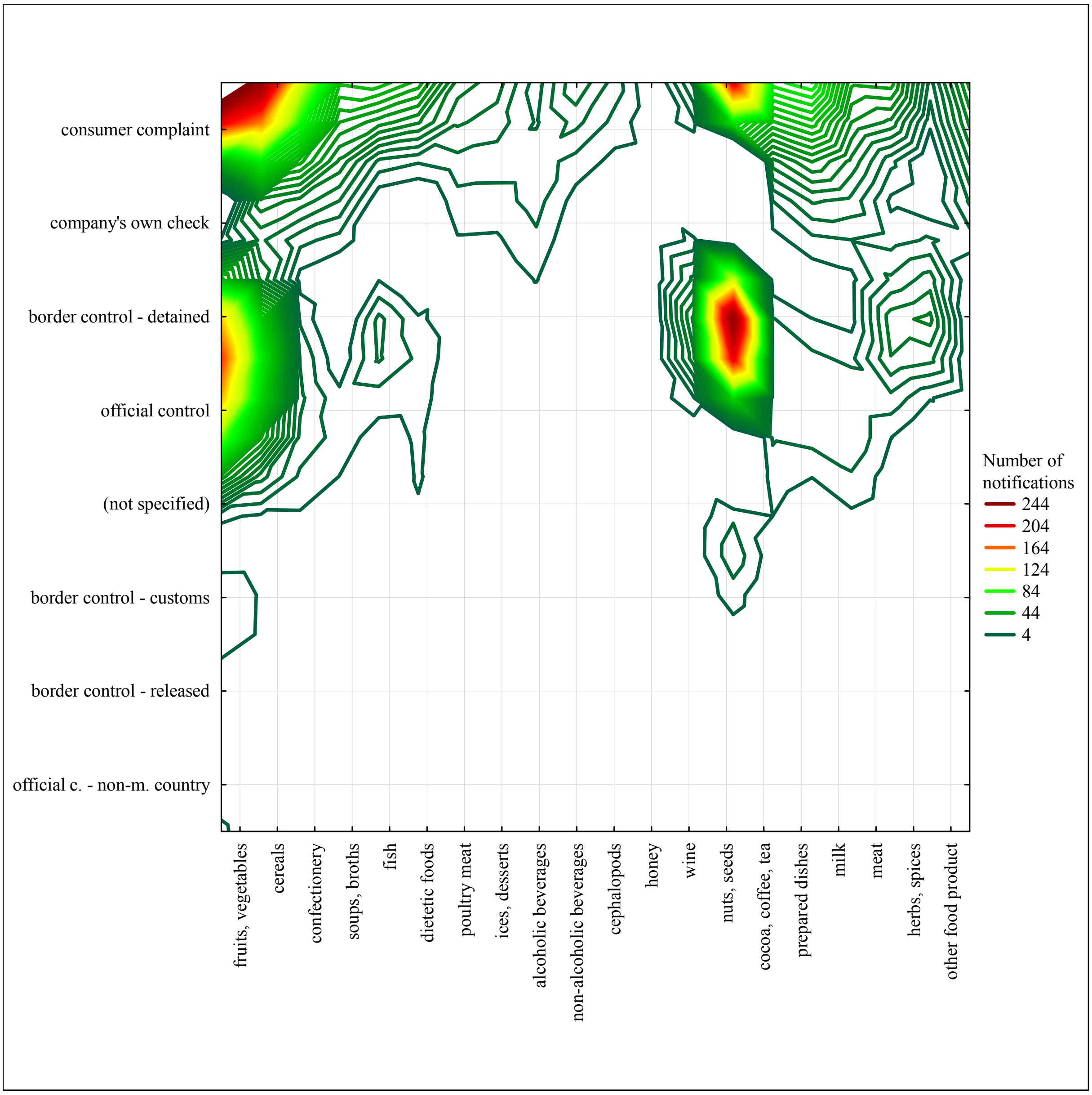

cephalopods – cephalopods and products thereof
cereals – cereals and bakery products
cocoa, coffee, tea – cocoa and cocoa preparations, coffee and tea
dietetic foods – dietetic foods, food supplements, fortified foods
fish – fish and fish products
fruits, vegetables – fruits and vegetables
herbs, spices – herbs and spices
honey – honey and royal jelly
ices, desserts – ices and desserts
meat – meat and meat products (other than poultry)
milk – milk and milk products
nuts, seeds – nuts, nut products and seeds
other food product – other food product / mixed
poultry meat – poultry meat and poultry meat products
prepared dishes – prepared dishes and snacks
soups, broths – soups, broths, sauces and condiments

border control - customs – border control - consignment under customs
border control - detained – border control - consignment detained
border control - released – border control - consignment released
official c. - non-m. country – official control in non-member country
official control – official control on the market

FIGURE S69 Similarities between product category and notification basis within notifications on foreign bodies.

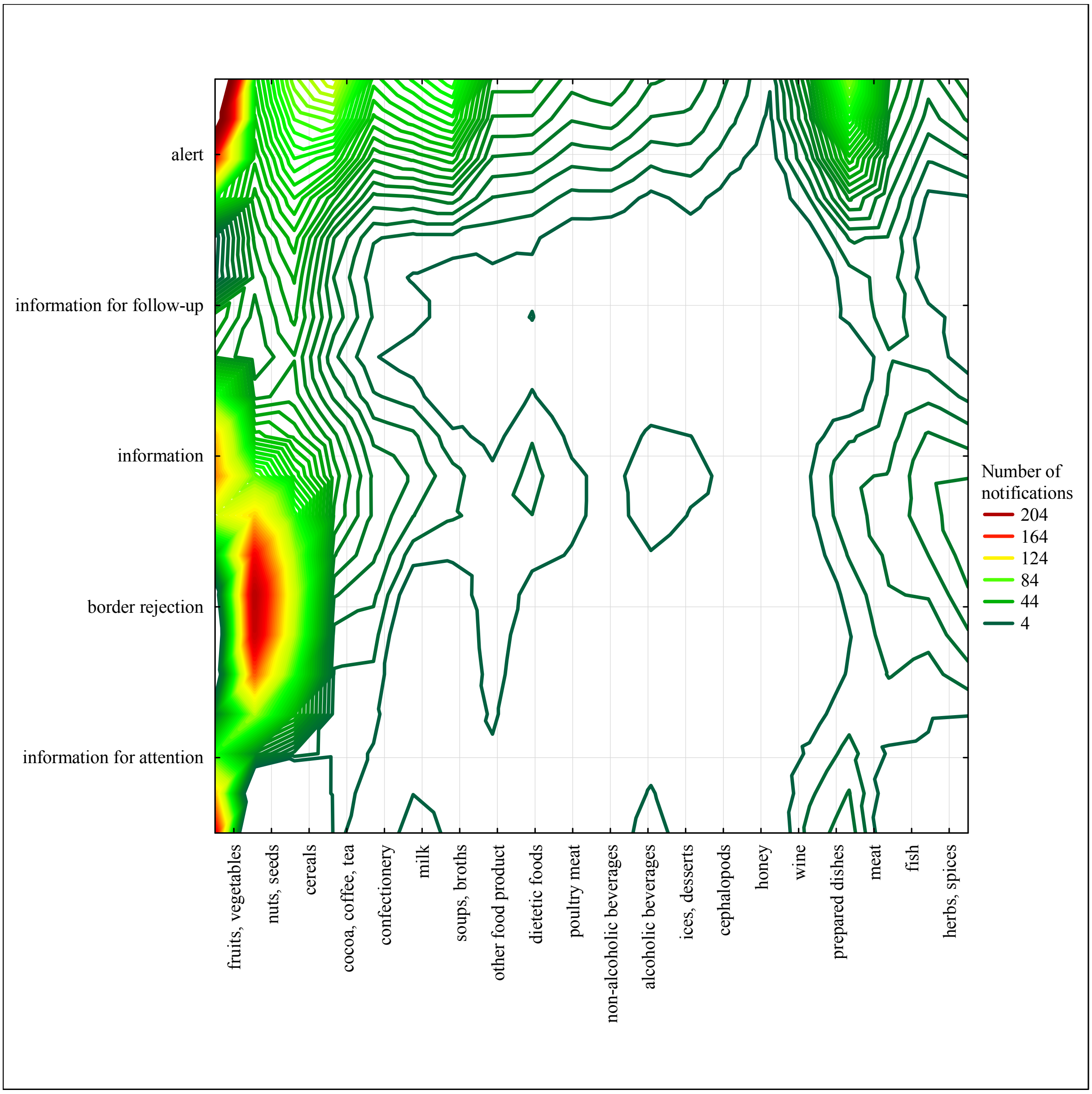

cephalopods – cephalopods and products thereof
cereals – cereals and bakery products
cocoa, coffee, tea – cocoa and cocoa preparations, coffee and tea
dietetic foods – dietetic foods, food supplements, fortified foods
fish – fish and fish products
fruits, vegetables – fruits and vegetables
herbs, spices – herbs and spices
honey – honey and royal jelly
ices, desserts – ices and desserts
meat – meat and meat products (other than poultry)
milk – milk and milk products
nuts, seeds – nuts, nut products and seeds
other food product – other food product / mixed
poultry meat – poultry meat and poultry meat products
prepared dishes – prepared dishes and snacks
soups, broths – soups, broths, sauces and condiments

FIGURE S70 Similarities between product category and notification type within notifications on foreign bodies.

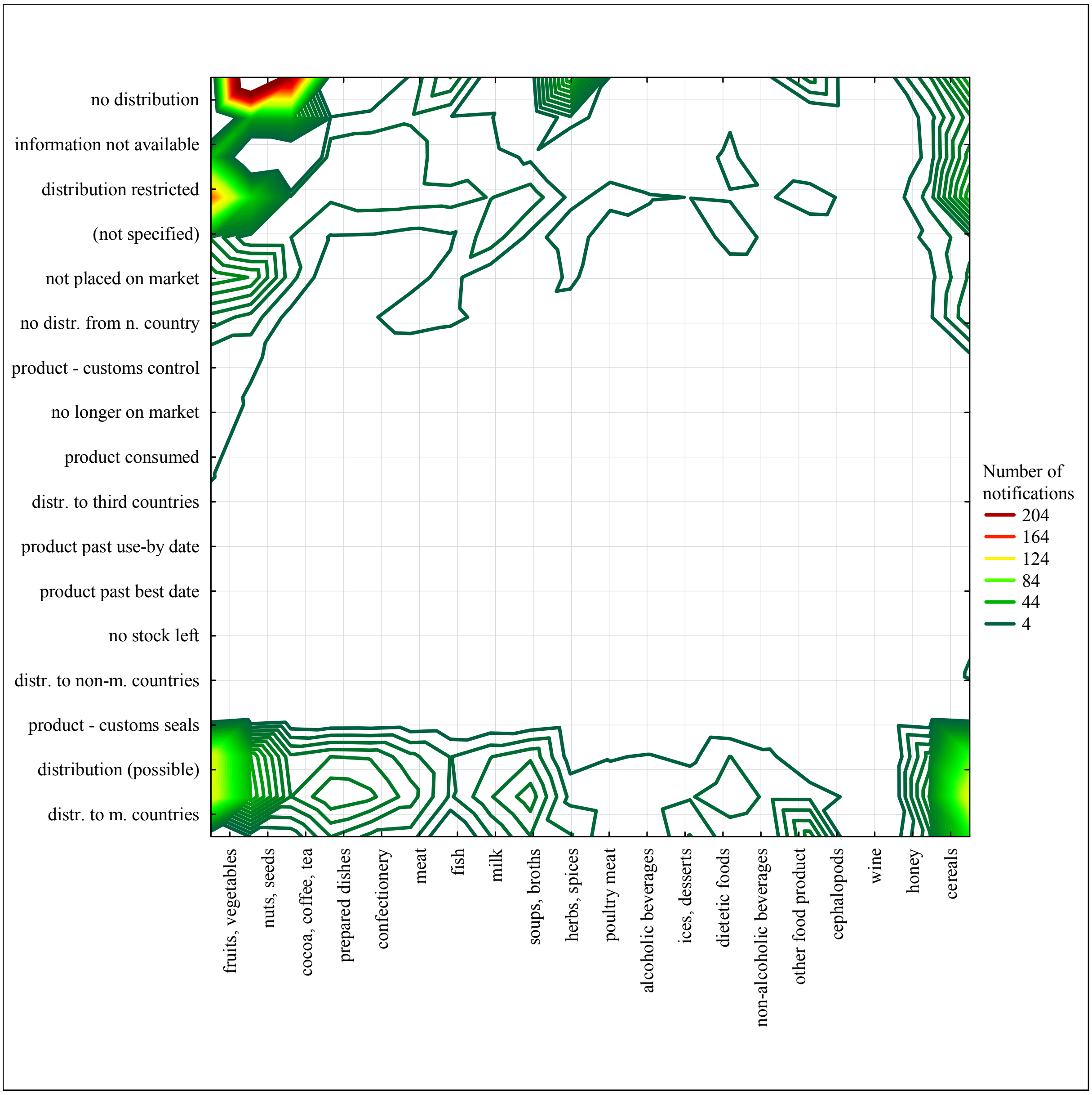

cephalopods – cephalopods and products thereof
cereals – cereals and bakery products
cocoa, coffee, tea – cocoa and cocoa preparations, coffee and tea
dietetic foods – dietetic foods, food supplements, fortified foods
fish – fish and fish products
fruits, vegetables – fruits and vegetables
herbs, spices – herbs and spices
honey – honey and royal jelly
ices, desserts – ices and desserts
meat – meat and meat products (other than poultry)
milk – milk and milk products
nuts, seeds – nuts, nut products and seeds
other food product – other food product / mixed
poultry meat – poultry meat and poultry meat products
prepared dishes – prepared dishes and snacks
soups, broths – soups, broths, sauces and condiments

distr. to m. countries – distribution to other member countries
distr. to non-m. countries – distribution to non-member countries
distr. to third countries – distribution to third countries
distribution (possible) – distribution on the market (possible)
distribution restricted – distribution restricted to notifying country
information not available – information on distribution not (yet) available
no distr. from n. country – no distribution from notifying country
no longer on market – product (presumably) no longer on the market
not placed on market – product not (yet) placed on the market
product - customs control – product under customs control
product - customs seals – product allowed to travel to destination under customs seals
product consumed – product already consumed
product past best date – product past best before date

FIGURE S71 Similarities between product category and distribution status within notifications on foreign bodies.

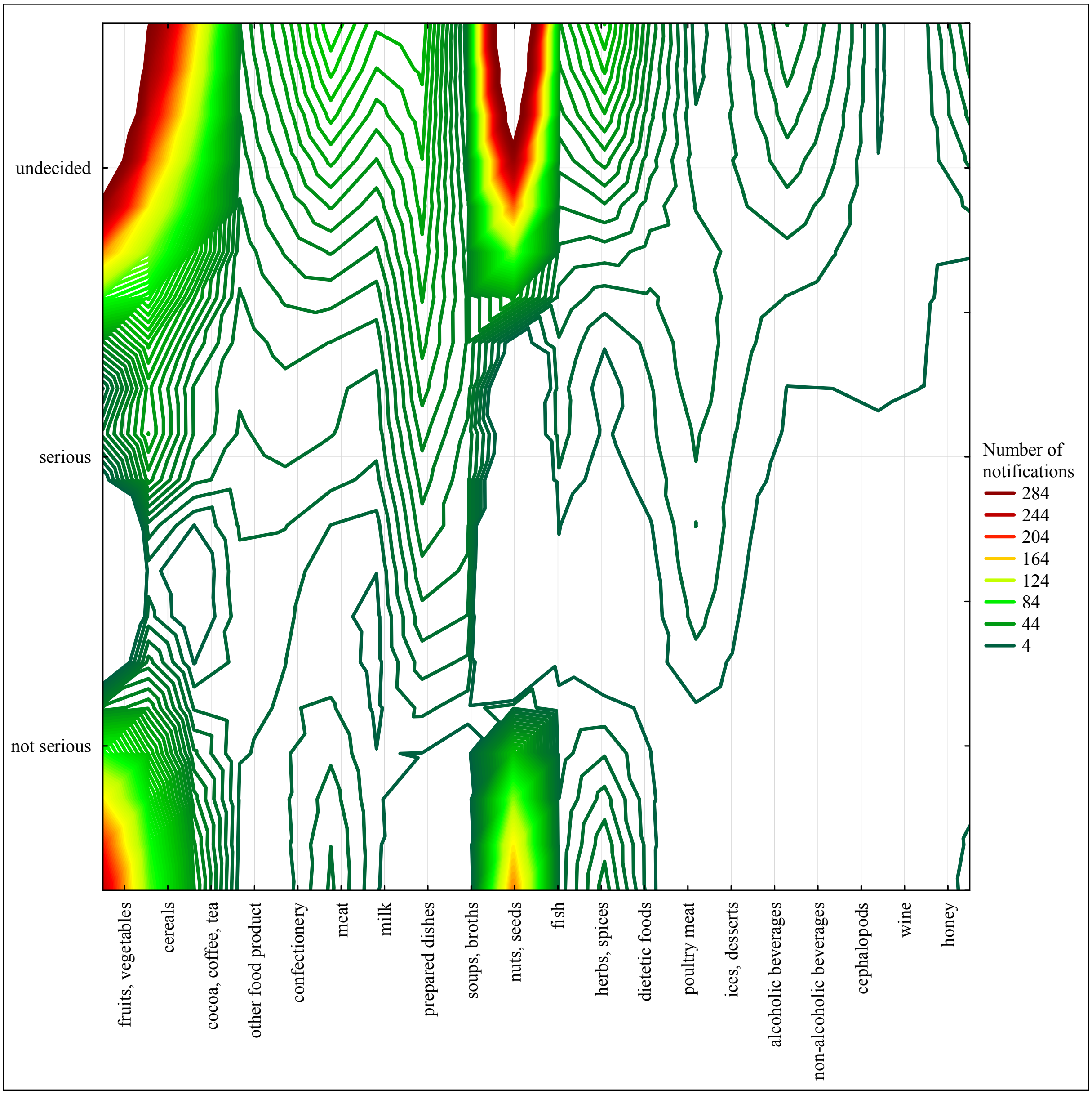

cephalopods – cephalopods and products thereof
cereals – cereals and bakery products
cocoa, coffee, tea – cocoa and cocoa preparations, coffee and tea
dietetic foods – dietetic foods, food supplements, fortified foods
fish – fish and fish products
fruits, vegetables – fruits and vegetables
herbs, spices – herbs and spices
honey – honey and royal jelly
ices, desserts – ices and desserts
meat – meat and meat products (other than poultry)
milk – milk and milk products
nuts, seeds – nuts, nut products and seeds
other food product – other food product / mixed
poultry meat – poultry meat and poultry meat products
prepared dishes – prepared dishes and snacks
soups, broths – soups, broths, sauces and condiments

FIGURE S72 Similarities between product category and risk decision within notifications on foreign bodies.

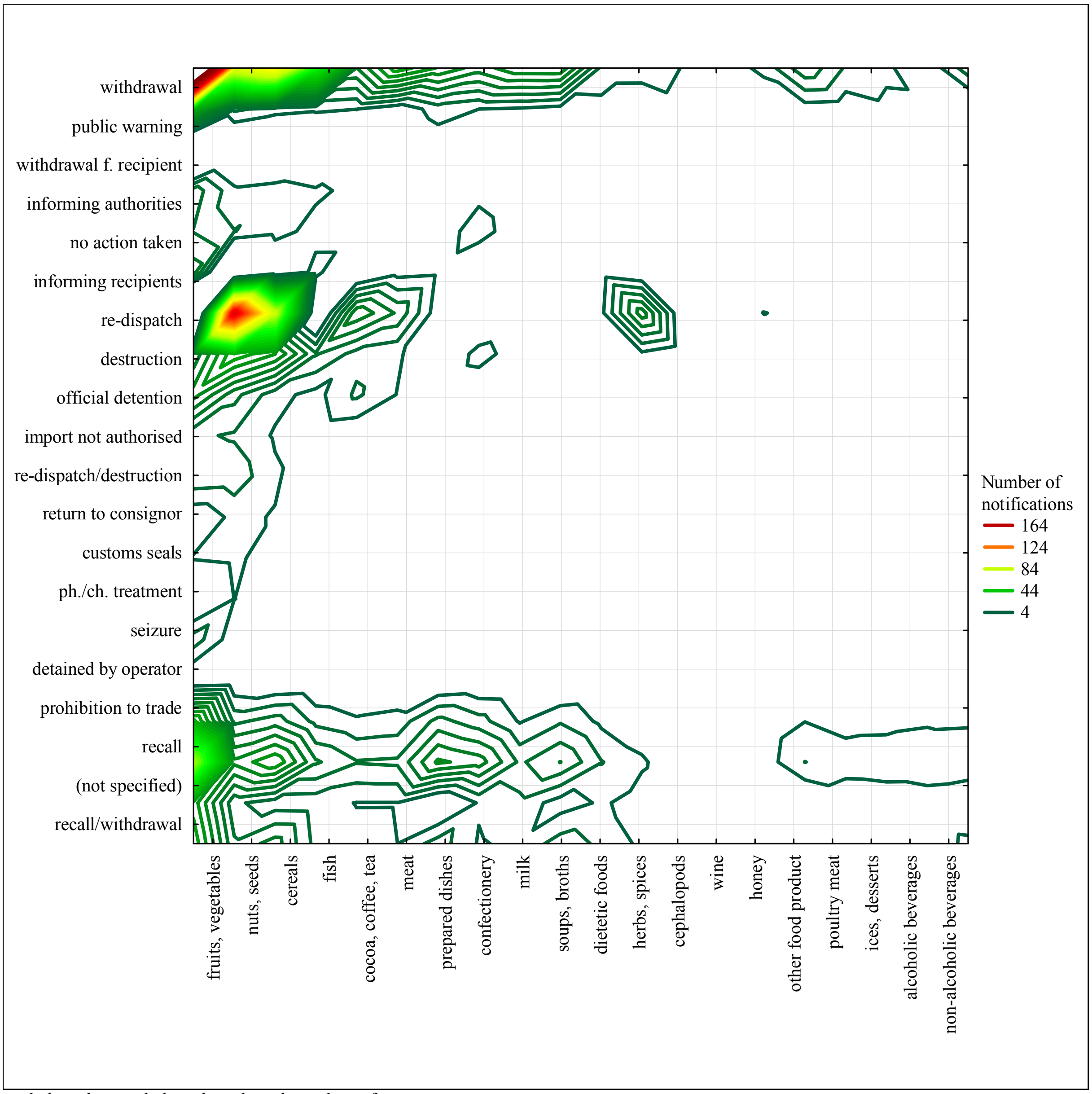

cephalopods – cephalopods and products thereof
cereals – cereals and bakery products
cocoa, coffee, tea – cocoa and cocoa preparations, coffee and tea
dietetic foods – dietetic foods, food supplements, fortified foods
fish – fish and fish products
fruits, vegetables – fruits and vegetables
herbs, spices – herbs and spices
honey – honey and royal jelly
ices, desserts – ices and desserts
meat – meat and meat products (other than poultry)
milk – milk and milk products
nuts, seeds – nuts, nut products and seeds
other food product – other food product / mixed
poultry meat – poultry meat and poultry meat products
prepared dishes – prepared dishes and snacks
soups, broths – soups, broths, sauces and condiments

customs seals – placed under customs seals
ph./ch. treatment – physical/chemical treatment
prohibition to trade – prohibition to trade - sales ban
public warning – public warning - press release
recall – recall from consumers
recall/withdrawal – product recall or withdrawal
re-dispatch/destruction – re-dispatch or destruction
withdrawal – withdrawal from the market
withdrawal f. recipient – withdrawal from recipient(s)

FIGURE S73 Similarities between product category and action taken within notifications on foreign bodies.
